# Statin use is associated with lower risk of dementia in stroke patients: a community-based cohort study with inverse probability weighted marginal structural model analysis

**DOI:** 10.1007/s10654-022-00856-7

**Published:** 2022-03-19

**Authors:** Zhirong Yang, Sengwee Toh, Xiaojuan Li, Duncan Edwards, Carol Brayne, Jonathan Mant

**Affiliations:** 1 Primary Care Unit, Department of Public Health and Primary Care, School of Clinical Medicine, University of Cambridge, Cambridge, UK; 2 Department of Population Medicine, Harvard Medical School &, Harvard Pilgrim Health Care Institute, Boston, MA USA; 3 Cambridge Public Health, School of Clinical Medicine, University of Cambridge, Cambridge, UK

**Keywords:** Statins, Stroke, Dementia, Cohort study

## Abstract

Current evidence is inconclusive on cognitive benefits or harms of statins among stroke patients, who have high risk of dementia. This observational cohort study investigated the association between statin use and post-stroke dementia using data from the Clinical Practice Research Datalink. Patients without prior dementia who had an incident stroke but received no statins in the preceding year were followed for up to 10 years. We used inverse probability weighted marginal structural models to estimate observational analogues of intention-to-treat (ITT, statin initiation vs. no initiation) and per-protocol (PP, sustained statin use vs. no use) effects on the risk of dementia. To explore potential impact of unmeasured confounding, we examined the risks of coronary heart disease (CHD, positive control outcome), fracture and peptic ulcer (negative control outcomes). In 18,577 statin initiators and 14,613 non-initiators (mean follow-up of 4.2 years), the adjusted hazard ratio (aHR) for dementia was 0.70 (95% confidence interval [CI] 0.64–0.75) in ITT analysis and 0.55 (95% CI 0.50–0.62) in PP analysis. The corresponding aHR_ITT_ and aHR_PP_ were 0.87 (95% CI 0.79–0.95) and 0.70 (95% CI 0.62–0.80) for CHD, 1.03 (95% CI 0.82–1.29) and 1.09 (95% CI 0.77–1.54) for peptic ulcer, and 0.88 (95% CI 0.80–0.96) and 0.86 (95% CI 0.75–0.98) for fracture. Statin initiation after stroke was associated with lower risk of dementia, with a potentially greater benefit in patients who persisted with statins over time. The observed association of statin use with post-stroke dementia may in part be overestimated due to unmeasured confounding shared with the association between statin use and fracture.

**Supplementary Information:**

The online version contains supplementary material available at 10.1007/s10654-022-00856-7.

## Background

While incident dementia affects 10% of patients with first-ever stroke and 30% with recurrent stroke [1], aggressive treatment of cardiovascular risk factors may potentially decrease the risk of post-stroke dementia [2]. Statins are recommended for the secondary prevention of atherosclerotic cardiovascular disease (ASCVD) including ischaemic stroke [3–5]; however, non-persistence with statin therapy remains a major challenge in practice [6, 7]. For intracerebral haemorrhage, there are insufficient data to support or restrict the use of statins in those patients [8].

Observational evidence suggests that statins may also help prevent dementia [9–13], but large-scale randomised controlled trials (RCTs) in people at high risk of cardiovascular events (most without stroke) did not find cognitive benefits (assessed using multiple cognitive tests as secondary outcomes) associated with statin use [14, 15]. Case reports [16–18] and small-scale trials [19, 20] have even suggested that statins may cause reversible cognitive impairment. The recent World Health Organisation (WHO) guideline classifies the current evidence on statin use for the prevention of dementia as low-quality [21].

To investigate whether statins could confer cognitive benefits or harms, stroke survivors would be a suitable population as many are likely to start statins and have multiple risk factors that put them at high risk of dementia [22]. Although some statin trials included cognitive assessment as secondary outcomes and enrolled a small proportion of stroke survivors [14, 15], no RCTs have specifically investigated the effect of statins on post-stroke dementia [23]. The only relevant trial published to date is the Prevention of Decline in Cognition after Stroke Trial (PODCAST), which investigated the effect of an intensive low-density lipoprotein (LDL) cholesterol target (< 1.3 mmol/L) versus guideline target (< 3.0 mmol/L) for preventing cognitive decline in 77 stroke patients [24]. The trial found that the intensive target significantly improved some cognitive test scores in the two-year follow-up, with statistically non-significant protective effect on dementia (odds ratio [OR] 0.18, 95% confidence interval [Cl] 0.01–3.98). A limited number of observational studies suggested that statin use may help prevent post-stroke dementia, but these studies are susceptible to biases due to inclusion of pre-stroke statin users and inappropriate control for confounding and selection bias [23].

It remains uncertain whether statins benefit or harm cognition in patients with stroke. We conducted a retrospective cohort study to examine the association of statin use with the risk of dementia among patients with an incident ischaemic stroke or intracerebral haemorrhage.

## Methods

### Data source

This study used Clinical Practice Research Datalink (CPRD) GOLD and its linked datasets, including hospitalisation data from integrated Hospital Episode Statistics (HES), death data from Office for National Statistics (ONS), and socioeconomic data from Index of Multiple Deprivation (IMD) datasets [25, 26]. The CPRD GOLD provides anonymised data extracted from primary care medical records, with coverage of a representative sample of approximately 7% of the UK population from more than 670 practices [25].

### Study population

We included patients aged 18 years or older with an incident stroke (hereafter referred to as index stroke) recorded in the CPRD between 1 January 2006 and 31 December 2017 (diagnosis codes for stroke are listed in Supplementary material of Appendix 1). We stratified stroke subtype into ischaemic stroke and intracerebral haemorrhage in our analysis. Patients were excluded if they had a diagnosis code indicating dementia any time before or within the first 90 days following the index stroke, had any codes related to statin prescription within one year before the index stroke, or had less than 12-month information recorded before the index stroke. In the main analysis, we also excluded those with any missing values for baseline covariates (i.e., smoking or body mass index [BMI]). For the analysis of each control outcome (described below), we additionally excluded patients who had any diagnosis codes related to the control outcome before the index stroke or within 90 days after the index stroke.

### Exposures

**Fig. 1:**
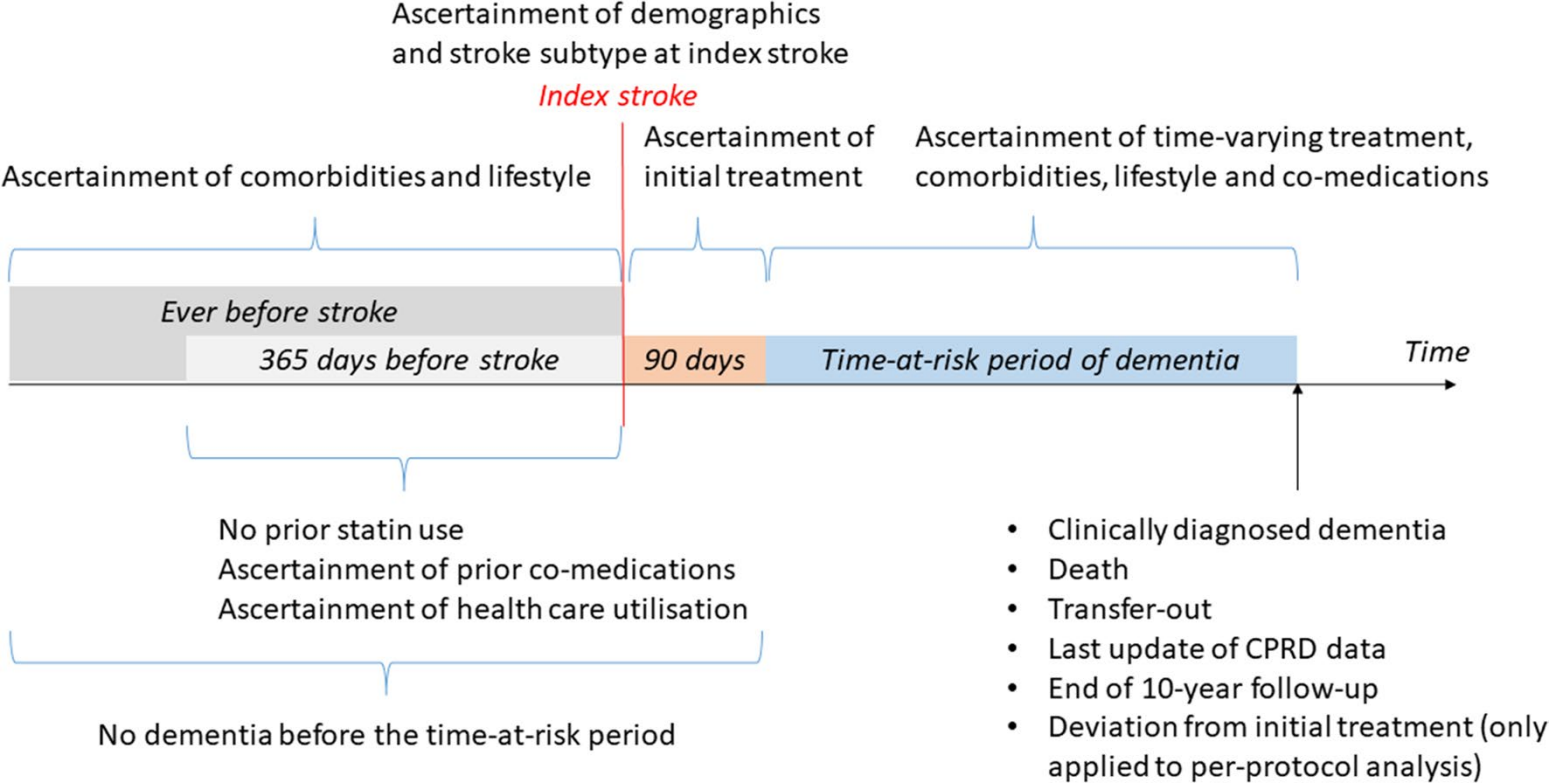
Time frames for ascertainment of exposure, outcome, and covariates. CPRD, Clinical Practice Research Datalink

The exposure of interest was statin initiation, defined as having any statin prescription within the first 90 days following the index stroke (Fig. 1). This time frame represents an early secondary prevention strategy and is also pragmatic to capture the first statin prescription in general practices after hospital stay. This approach also reduced misclassification of statin initiation given the vast majority of people with stroke should have been discharged within this time frame and had visited their general practitioners for follow-up care. The product codes for statins are shown in Supplementary material of Appendix 1. Patients who received no statins within this time period were included in the statin non-initiation group. The exposure status was then treated as a time-varying variable (updated every 30 days, starting from the 91st day following the index stroke). Statin use was considered sustained until no further statin prescription within the expected end of days-supply (30 days of the previous prescription) plus a 90-day grace period.

### Outcomes

The primary outcome of interest was incident post-stroke dementia. We adopted a current consensus definition of post-stroke dementia, which includes any subtype of dementia following stroke [2]. Clinical diagnosis of dementia was determined based on Read codes or International Classification of Diseases, 10th revision (ICD-10) diagnosis codes relating to dementia (Supplementary material of Appendix 1) first recorded in the CPRD, HES, or ONS on or after the 91st day following index stroke (Fig. 1).

To explore the potential impact of unmeasured confounding on the risk of dementia associated with statin use, we used coronary heart disease (CHD) as a positive control outcome and fracture and peptic ulcer as two negative control outcomes. The positive control outcome was expected to be affected by statin use while the negative control outcomes were not. These control outcomes were chosen because a similar set of confounders is shared by the relationship between statin use and each control outcome and that between statin use and dementia, which may or may not have been captured in our study, and high-quality evidence from RCTs has established the impact of statin use on these outcomes. Statin use has been shown to reduce the risk of CHD in patients with cerebrovascular disease in the Stroke Prevention by Aggressive Reduction in Cholesterol Levels (SPARCL) trial and in the Heart Protection Study (HPS) [27, 28]. While observational studies tended to find an inverse association with fracture (likely due to unmeasured confounding and other biases) [29], statins had no effect on the risk of incident fracture in the HPS or in the Justification for the Use of statins in Prevention: an Intervention Trial Evaluating Rosuvastatin (JUPITER) trial [14, 30]. Statins were not associated with any gastrointestinal disorders in SPARCL and HPS or gastrointestinal bleeding in the Study of Heart and Renal Protection (SHARP) trial, which would mainly occur in peptic ulcers [14, 31, 32]. Thus, we expected to observe an inverse association between statin use and CHD but no association of statin use with fracture or peptic ulcer in the absence of residual confounding and other biases in our analysis. These control outcomes were identified using Read codes or ICD-10 diagnosis codes (Supplementary material of Appendix 1) first recorded in the CPRD, HES or ONS on or after the 91st day following index stroke.

### Follow-up

Patients in each group who survived beyond the first 90 days of the index stroke contributed to the time-at-risk period of dementia (Fig. 1). For the observational analogue of intention-to-treat (ITT) effect analysis (described below), patients were followed until the date of incident dementia or the date of censoring (death, transfer-out, the last update of the CPRD data [31 July 2018], or the end of 10-year follow-up), whichever occurred first. The whole follow-up period for each patient was split into discrete 30-day (one-month) intervals. In each interval, information was updated on covariates, outcome, and censoring.

For the observational analogue of per-protocol (PP) effect analysis (described below), patients were additionally artificially censored if they discontinued statin treatment in the statin initiation group or started statin treatment in the non-initiation group. The artificial censoring date was the expected end of days-supply (typically 30 days) of the last prescription plus a 90-day grace period for the statin initiators and was the prescription date of the first statin for the non-initiators.

### Potential confounders

Depending on the ascertainment time window, three sets of covariates were considered in our analysis for confounding adjustment: baseline characteristics, characteristics by the 90th day of index stroke, and time-varying characteristics (Fig. 1). The baseline covariates included demographics (age, gender, and socioeconomic status measured by Index of Multiple Deprivation [IMD]), comorbidities (atrial fibrillation, alcohol use disorders, CHD, diabetes, heart failure, hyperlipidaemia, hypertension, peripheral artery disease, transient ischemic attack, anxiety, asthma, chronic obstructive pulmonary disease, depression, epilepsy, Parkinson’s disease and rheumatoid arthritis), lifestyle factors (smoking and BMI), healthcare utilisation, and co-medications (anticoagulant, antiplatelet, antidiabetic, antihypertensive and non-statin lipid-lowering treatments). The by-90^th^-day and time-varying covariates included comorbidities, lifestyle factors, and co-medications. Definitions of these covariates are detailed in Supplementary material of Appendix 2.

### Statistical analysis

All analyses were conducted using Stata 15 after quality control (Supplementary material of Appendix 3). Baseline characteristics were compared between the two exposure groups using standardised mean difference (with an absolute value greater than 0.1 indicating meaningful imbalance) [33]. Incidence rate of post-stroke dementia was calculated for each group.

We estimated two types of observational effects of statin use on post-stroke dementia: the effect of initiation versus no initiation (observational analogue of ITT effect) and the effect of sustained use versus no use (observational analogue of PP effect). We estimated the hazard ratios (HRs) and their 95% CIs for both effects using inverse probability weighted marginal structural models in a two-stage process. First, we estimated four types of stabilised weights using a logistic regression: baseline treatment weights (to adjust for baseline confounding), baseline selection weights (for baseline selection bias due to the requirement to survive the first 90 days following the index stroke), follow-up weights (for loss to follow-up) and treatment persistence weights (for non-persistence with the initial treatment). In the second stage, we estimated the two observational effects of interest using a weighted pooled logistic regression model, which approximates a time-dependent Cox model [34]. In the ITT analysis, the primary model was conducted with full adjustment for baseline characteristics, baseline selection bias, and loss to follow-up using the product of baseline treatment weight, baseline selection weight, and follow-up weight. In the PP analysis, the primary model additionally accounted for artificial censoring due to deviation from initial treatment during the time-at-risk period using the product of the four weights. The final weights used in the outcome models were truncated at 1st and 99th percentiles to minimise the impact of extreme weights and to improve precision [35]. We estimated the 95% CIs using a robust variance estimator to account for potential correlation introduced by weighting. Details of the two-step estimation approach are described in Supplementary material of Appendix 4.

To explore how the associations of statin initiation and sustained use with dementia were influenced by more adjustment for potential biases, we fit two nested models: the first model only adjusted for baseline confounding using the baseline treatment weights and the second model additionally adjusted for baseline selection bias using the product of baseline treatment weights and baseline selection weights.

To explore the potential impact of unmeasured confounding on the association between statin use and dementia, we repeated the main analysis of ITT and PP effects for the three control outcomes, with all types of weights re-estimated.

We conducted subgroup analysis to explore whether the fully adjusted estimates for dementia differed by age group (18–64, 65–74, 75–84, and ≥ 85 years), gender, stroke subtype (ischaemic and haemorrhagic), and baseline cardiovascular risk factors including atrial fibrillation, heart failure, CHD, peripheral artery disease, transient ischaemic attack, diabetes, hyperlipidaemia, and hypertension. We tested the subgroup difference by including an interaction term to the primary full adjustment models, in which a Bonferroni correction was applied to the significance level that divided 0.05 by 11 subgroups examined (i.e., 0.0045).

Several sensitivity analyses were conducted to examine the robustness of the main results: (1) using original weights, truncation of weights at 0.5th and 99.5th percentiles, or truncation at 0.1 and 10; (2) instead of excluding patients with missing baseline covariates, imputing the missing baseline BMI with 5th or 95th percentiles and assuming missing smoking status as never smoking; (3) separating unspecified stroke from ischaemic stroke subtype; (4) excluding patients without linkage to HES; (5) further excluding any patients with dementia occurring between months 4 and 6 following the index stroke; (6) varying the time window for statin initiation assessment to 1 or 6 months after index stroke, with the time-at-risk period of dementia starting from the 2nd or 7th month of stroke, respectively; and (7) varying the grace period to 1 or 5 months for defining statin discontinuation in the PP analysis. (8) As our negative control outcome analysis showed a significant association between statin use and fracture, we conducted a post-hoc exploratory analysis by including fracture in our weight models, and also performed an indirect adjustment for unmeasured confounding by assuming that the potential associations between the unmeasured confounders and dementia and fracture would be similar in magnitude [36]. For sensitivity analyses (2) to (8), we re-estimated all types of weights considering the study population, statin initiation definition, covariates, or treatment persistence definition were different from those in the main analysis.

## Results

### Patient inclusion and baseline characteristics

**Fig. 2:**
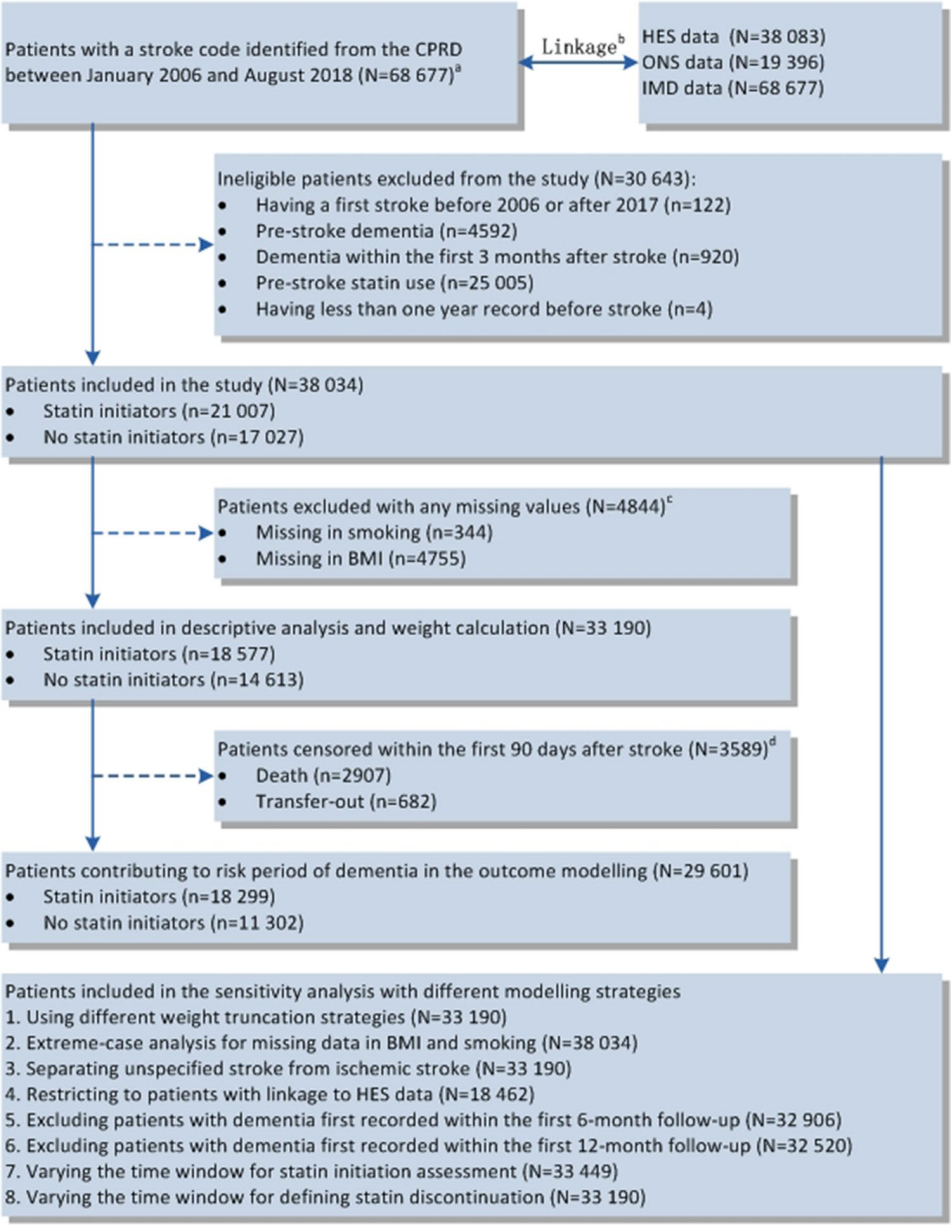
Flow chart of patient inclusion. **a** All the 68,677 patients identified in the CPRD were considered potentially eligible, regardless of whether they could be linked to other data sources. **b** Only those who had consented to participate in the CPRD linkage scheme had HES data (covering about 58% of all UK CPRD practices). Only those who died had linked ONS data. 38,616 patients had patient-level IMD and the other 30,061 patients had practice-level IMD. **c** The number was not exclusive, with missing smoking data in 344 patients and missing BMI in 4755 patients. **d** To control potential selection bias, censoring was accounted for using inverse probability weighting in the adjustment models. CPRD, Clinical Practice Research Datalink; HES, Hospital Eisode Statistics; IMD, Index of Multiple Deprivation; ONS, Office for National Statistics

Of 68,677 patients with an incident stroke recorded in the CPRD, 33,190 (mean age, 71.9 years, with about 96.0% of patients aged 45 years or older; female, 51.9%) were included in the study (Fig. 2). Among these eligible patients, 18,577 patients (56.0%) received a statin during the first 90 days after the index stroke (Table 1). Statin initiators were more likely to be younger, males, current smokers, suffer an ischaemic stroke, and have a higher BMI. They tended to have fewer CHD, atrial fibrillation, diabetes, heart failure, hypertension, hearing loss, and cardiovascular related co-medications. Patients excluded from the main analysis due to missing data at baseline (n = 4844) tended to have fewer comorbidities and receive less pre-stroke treatment (Supplementary material of Appendix 5). Within the first 90 days following stroke, 133 patients died and 145 transferred out of their practices in the statin group, while 2774 died and 537 transferred out in the no statin group. Patients who survived during this period tended to have fewer comorbidities and receive less pre-stroke treatment (Supplementary material of Appendix 5).

**Table 1:** Baseline characteristics of the two treatment groups

Characteristics* (number [%])	Total (N = 33,190)	Statin initiation (N = 18,577)	No statin initiation (N = 14,613)	SMD**
*Demographics*				
Age at stroke, median (IQR)	74 (62–83)	72 (61–81)	78 (65–86)	0.27
Female	17,231 (51.9)	9084 (48.9)	8147 (55.8)	0.14
IMD Group 1 (least deprived)	7174 (21.6)	4047 (21.8)	3127 (21.4)	0.04
Group 2	6263 (18.9)	3471 (18.7)	2792 (19.1)	
Group 3	7238 (21.8)	3963 (21.3)	3275 (22.4)	
Group 4	6644 (20.0)	3809 (20.5)	2835 (19.4)	
Group 5	5871 (17.7)	3287 (17.7)	2584 (17.7)	
*Stroke subtype*				
Ischaemic^#^	30,113 (90.7)	17,906 (96.4)	12,207 (83.5)	0.44
Haemorrhagic	3077 (9.3)	671 (3.6)	2406 (16.6)	
Cardiovascular factors***				
AF	6056 (18.2)	2657 (14.3)	3399 (23.3)	0.23
Alcohol use disorders	1651 (5.0)	871 (4.7)	780 (5.3)	0.03
CHD	3777 (11.4)	1527 (8.2)	2250 (15.4)	0.22
Diabetes	3069 (9.2)	1419 (7.6)	1650 (11.3)	0.13
Heart failure	2023 (6.1)	648 (3.5)	1375 (9.4)	0.24
Hyperlipidaemia	4945 (14.9)	2797 (15.1)	2148 (14.7)	0.01
Hypertension	16,871 (50.8)	8988 (48.4)	7883 (53.9)	0.11
PAD	1099 (3.3)	489 (2.6)	610 (4.2)	0.09
TIA	2851 (8.6)	1611 (8.7)	1240 (8.5)	0.01
Smoking current	6807 (20.5)	4311 (23.2)	2496 (17.0)	0.16
Former	10,104 (30.4)	5258 (28.3)	4846 (33.2)	
Never	16,279 (49.1)	9008 (48.5)	7271 (49.8)	
BMI, median (IQR)	26.1 (23.1–29.4)	26.4 (23.4–29.7)	25.6 (22.6–29.1)	0.14
Other comorbidities***				
Anxiety	6420 (19.3)	3518 (18.9)	2902 (19.9)	0.02
Rheumatoid arthritis	2178 (6.6)	1060 (5.7)	1118 (7.7)	0.08
Asthma	4328 (13.0)	2368 (12.7)	1960 (13.4)	0.02
COPD	2976 (9.0)	1550 (8.3)	1426 (9.8)	0.05
Depression	8679 (26.1)	4724 (25.4)	3955 (27.1)	0.04
Epilepsy	1000 (3.0)	458 (2.5)	542 (3.7)	0.07
Hearing loss	7205 (21.7)	3626 (19.5)	3579 (24.5)	0.12
Parkinson’s disease	406 (1.2)	144 (0.8)	262 (1.8)	0.09
Healthcare utilisation***				
Number of consultations, median (IQR)	28 (16–45)	25 (14–39)	33 (19–51)	0.35
Co-medications***				
Anticoagulant drugs	1977 (6.0)	695 (3.7)	1282 (8.8)	0.21
Antiplatelet drugs	7644 (23.0)	3617 (19.5)	4027 (27.6)	0.19
Antidiabetic drugs	1948 (5.9)	889 (4.8)	1059 (7.2)	0.10
Antihypertensive drugs	17,272 (52.0)	8954 (48.2)	8318 (56.9)	0.18
Other lipid-lowering drugs	712 (2.1)	219 (1.2)	493 (3.4)	0.15

*AF* atrial fibrillation, *BMI* body mass index, *CHD* coronary heart disease, *COPD* chronic obstructive pulmonary disease, *IMD* Index of Multiple Deprivation, *PAD* peripheral artery disease, *SMD* standardised mean difference, *SD* standard deviation, *TIA* transient ischaemic attack

*Data are expressed as counts (percentages), except for age, BMI and number of consultations

**SMD is expressed as an absolute value (greater than 0.1 indicating meaningful imbalance)

***Cardiovascular factors and other comorbidities were defined up until the index stroke; health care utilisation and co-medications were defined within one years before the index stroke

^#^A total of 14,352 (43.2%) patients had an unspecified stroke subtype, with 8054 (43.4%) and 6298 (43.1%) in statin and no statin group, respectively

### Incidence of post-stroke dementia

Among the 29,601 patients who survived through the first 90 days after the index stroke, 3176 patients (10.7%) were diagnosed with dementia over a mean follow-up of 4.8 years (5.2 years for statin initiators and 4.2 years for statin non-initiators). The incidence of post-stroke dementia was 18.0 per 1000 person-years and 30.6 per 1000 person-years, respectively (Table 2 and Supplementary material of Appendix 6).

**Table 2:** Effect estimates from ITT and PP analysis for post-stroke dementia

	ITT analysis		PP analysis^#^	
	Statin initiation	No statin initiation	Statin sustained use	No statin use
Number of survivors at day 90	18,299	11,302	18,299	11,302
Number of PSD	1727	1449	953	1071
Person-years	96,007	47,402	56,697	30,631
Unadjusted rate, /10^3^ person-years	18.0	30.6	16.9	35.0
cHR (95% CI)*	0.58 (0.54–0.62)	Reference	0.47 (0.43–0.52)	Reference
aHR (95% CI)** Model 1^a^	0.72 (0.67–0.77)	Reference	0.61 (0.56–0.67)	Reference
Model 2^b^	0.71 (0.66–0.76)	Reference	0.60 (0.54–0.66)	Reference
Model 3^c^	0.70 (0.64–0.75)	Reference	0.55 (0.50–0.62)	Reference

*aHR* adjusted hazard ratio, *cHR* crude hazard ratio, *CI* confidence interval, *ITT* intention-to-treat, *PP* per-protocol, *PSD* post-stroke dementia

*Of 33,190 eligible patients, 29,601 patients who survived at month 3 were included in the outcome model

**33,190 eligible patients contributed to weight calculation. Of whom, 29,601 patients who survived at month 3 were included in the outcome model

^#^Deviation from initial treatment during follow-up was artificially censored in all the models

^a^Model 1: adjusted for baseline characteristics and months of follow-up

^b^Model 2: adjusted for baseline selection plus Model 1

^c^Model 3: adjusted for loss to follow-up plus Model 2. For PP analysis, artificial censoring due to deviation from initial treatment during time-at-risk period was additionally accounted for

### Association of statin use with post-stroke dementia

Compared with the crude model, adjustment for baseline characteristics (Model 1) led to a large numerical difference in effect estimate (Table 2). Progressive adjustment for baseline selection and loss to follow-up (Models 2 and 3) did not lead to further large changes. In the fully adjusted model (Model 3), statin initiation was associated with a 30% (95% CI 25%-36%, *P* < 0.001) reduction in the risk of dementia compared with no statin initiation (aHR_ITT_ 0.70, 95% CI 0.64–0.75, *P* < 0.001) (Table 2). When non-persistence was further accounted for, sustained statin use was associated with a 45% (95% CI 38–50%, *P* < 0.001) reduction in the risk of dementia (aHR_PP_ 0.55, 95% CI 0.50–0.62, *P* < 0.001) compared to no statin use. The estimated stabilised weights were statistically well distributed, with a mean of 1 and no extremely large weights after truncation at 1st and 99th percentiles (Supplementary material of Appendix 7), suggesting no gross misspecification of the weight models.

### Association of statin use with control outcomes

The parallel analyses for CHD (positive control outcome) and peptic ulcer (negative control outcome) suggested that unmeasured confounders which may bias the association of statin use with these two outcomes were not likely to distort the inverse association between statin use and dementia (Table 3). In the full adjustment models, statin initiation was associated with decreased risk of CHD (aHR_ITT_ 0.87, 95% CI 0.79–0.95; aHR_PP_ 0.70, 95% CI 0.62–0.80). There was no evidence of an association between statin use and peptic ulcer (aHR_ITT_ 1.03, 95% CI 0.82–1.29; aHR_PP_ 1.09, 95% CI 0.77–1.54) but evidence of a lower risk of fracture with statin use (aHR_ITT_ 0.88, 95% CI 0.80–0.96; aHR_PP_ 0.86, 95% CI 0.75–0.98). Weights after truncation at 1st and 99th percentiles used in the models were statistically well distributed, with means close to one and no extremely large weights (Supplementary material of Appendix 7).

**Table 3:** Effect estimates from ITT and PP analysis for control outcomes

	CHD*	Fracture*	Peptic ulcer*
*ITT analysis*			
cHR (95% CI)	0.81 (0.75–0.89)	0.78 (0.72–0.85)	0.97 (0.79–1.19)
aHR (95% CI) Model 1^a^	0.89 (0.81–0.97)	0.90 (0.82–0.99)	1.05 (0.84–1.31)
Model 2^b^	0.87 (0.80–0.95)	0.89 (0.81–0.97)	1.03 (0.83–1.29)
Model 3^c^	0.87 (0.79–0.95)	0.88 (0.80–0.96)	1.03 (0.82–1.29)
*PP analysis*^*#*^			
cHR (95% CI)	0.68 (0.61–0.76)	0.78 (0.69–0.87)	1.07 (0.82–1.39)
aHR (95% CI) Model 1^a^	0.76 (0.68–0.85)	0.92 (0.81–1.04)	1.19 (0.89–1.58)
Model 2^b^	0.74 (0.66–0.84)	0.90 (0.80–1.02)	1.15 (0.87–1.53)
Model 3^c^	0.70 (0.62–0.80)	0.86 (0.75–0.98)	1.09 (0.77–1.54)

*aHR* adjusted hazard ratio, *CHD* coronary heart disease, *cHR* crude hazard ratio, *CI* confidence interval, *ITT* intention-to-treat, *PP* per-protocol

*CHD: Of 28,946 eligible patients (contributing to weight calculation), 26,098 patients who survived at month 3 were included in the outcome model

Fracture: Of 22,850 eligible patients (contributing to weight calculation), 20,545 patients who survived at month 3 were included in the outcome model

Peptic ulcer: Of 31,042 eligible patients (contributing to weight calculation), 27,647 patients who survived at month 3 were included in the outcome model

^#^Deviation from initial treatment during follow-up was artificially censored in all the models

^a^Model 1: adjusted for baseline characteristics and months of follow-up

^b^Model 2: adjusted for baseline selection plus Model 1

^c^Model 3: adjusted for loss to follow-up plus Model 2. For PP analysis, artificial censoring due to deviation from initial treatment during time-at-risk period was additionally accounted for

### Subgroup analysis

**Fig. 3:**
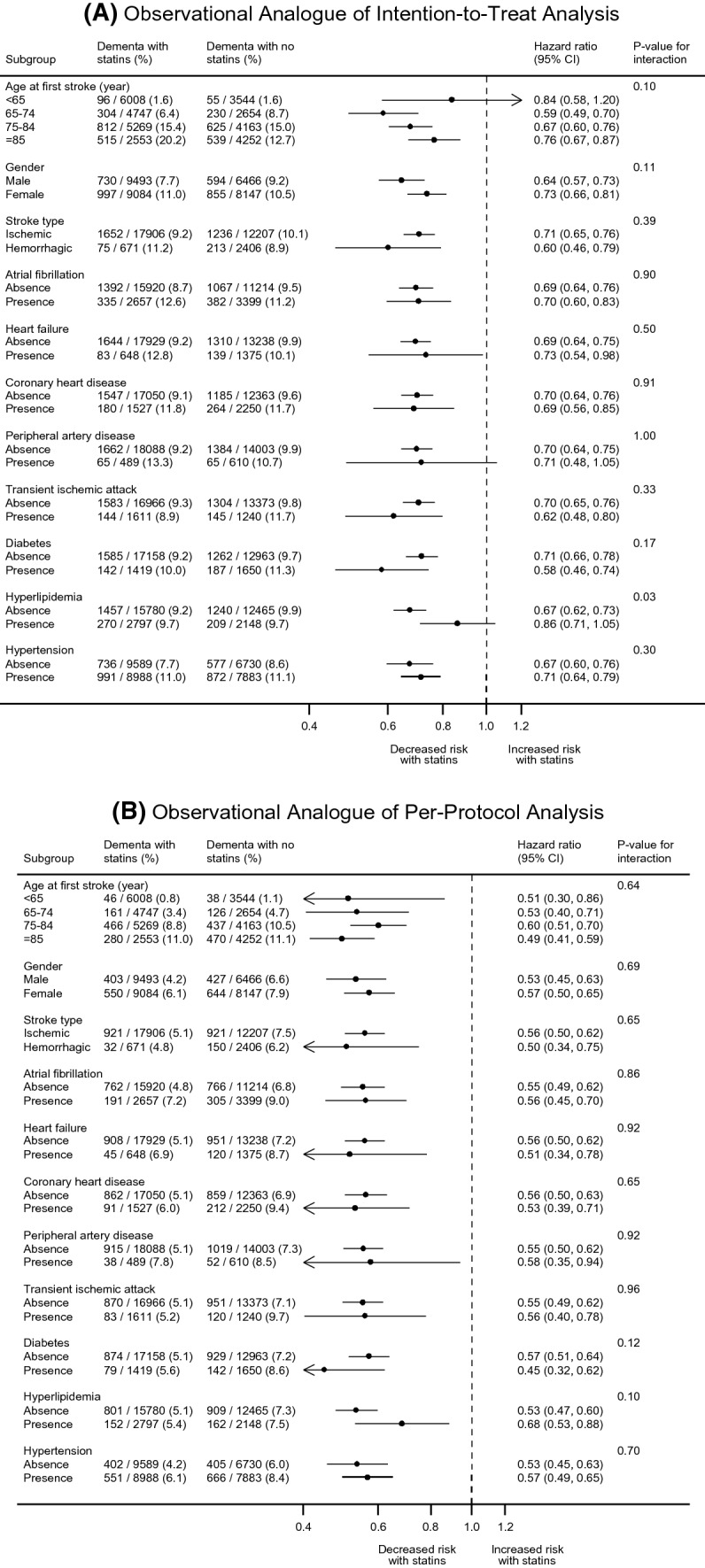
Subgroup analysis for statin use. All the outcome models adjusted for baseline characteristics, baseline selection, loss to follow-up and months of follow-up. For PP analysis, artificial censoring due to deviation from initial treatment during time-at-risk period was additionally accounted for. CI, confidence interval

The association of statin initiation with post-stroke dementia was stronger in patients with no hyperlipidaemia than in those with hyperlipidaemia in the ITT analysis (*P* for interaction = 0.031) (Fig. 3). However, the difference became statistically non-significant after Bonferroni correction. No differences were observed between stroke subtypes or other pre-specified subgroups in the ITT or PP analysis.

### Sensitivity analysis

In general, results from the main analysis were robust under different sensitivity analyses (Supplementary material of Appendix 8). The only difference between the main analysis and sensitivity analyses was that the association of statin use with CHD (in ITT analysis) and fracture (in PP analysis) was not significant when the original (i.e., untruncated) weights were used in the models (Supplementary material of Appendix 8). However, the point estimates changed little with different weight truncation criteria. When including fracture in the full adjustment model for dementia, there was little change to our main findings in both ITT and PP analysis (aHR_ITT_ 0.70, 95% CI 0.61–0.80; aHR_PP_ 0.55, 95% CI 0.46–0.66). By assuming the impact of unmeasured confounding on the risk of dementia associated with statin use was similar to that of fracture, the fully adjusted association for dementia attenuated but remained significant (aHR_ITT_ 0.80, 95% CI 0.70–0.90; aHR_PP_ 0.64, 95% CI 0.54–0.76).

## Discussion

### Summary of principal findings

In this study, statin initiation within the first 3 months after an incident stroke (most aged at least 45 years) was associated with a decreased risk of dementia (ITT analysis). When additionally accounting for treatment persistence over time, the magnitude of effect estimate became stronger, with about 50% reduction in the risk of post-stroke dementia in patients with sustained use of statins (PP analysis). One possible explanation is that an ITT analysis is generally more susceptible to exposure misclassification during follow-up, which would bias the results toward the null in our statin use versus non-use comparison. The findings may also suggest enhanced persistence with statin use could potentially lower the risk of post-stroke dementia. There was no clear evidence that the association of statin use with risk of dementia differed by stroke subtype or other pre-specified baseline patient characteristics examined in our subgroup analysis. The observed association may be overestimated in part due to unmeasured confounding which could also affect the association of statin use with fracture but not that with CHD or peptic ulcer. However, indirect adjustment for unmeasured confounding did not substantially change the inverse association. A series of sensitivity analysis to explore the impact of potential bias sources did not show any appreciable changes to our main findings. It should be noted that statin initiators in our study were younger and had fewer comorbidities than no statin initiators (Table 1) due to exclusion of pre-stroke statin users, a design decision aimed to reduce prevalent-user bias [37, 38] but could reduce the generalisability of our main findings to all stroke patients [39].

### Strengths and limitations

Compared with previous studies, this study included the largest sample size to date to investigate the association between statin use and post-stroke dementia. This study used a clear definition of statin initiation, new-user design (excluding prevalent statin users) and inverse probability weighted marginal structural models to account for potential selection bias and confounding. Control outcomes were used to assess the impact of unmeasured confounding. This study also benefits from the strengths of the CPRD data, such as representativeness of real practice settings, detailed prescription information, large sample size, and long-term follow-up [25].

However, there are some limitations in this study. First, unmeasured confounding likely existed as indicated by an observed association of statins with fracture (a negative control outcome). Unmeasured confounders shared between both associations for dementia and fracture in our study likely include (but are not limited to) recurrent stroke, ethnicity, education attainment, physical activity, dietary/supplementation (such as vitamin D and calcium), and baseline cognitive function [40, 41], which are poorly recorded in the CPRD and HES. We did not have information on stroke severity, which may be associated with both statin initiation and dementia risk. However, our sensitivity analysis restricting to those who survived for at least six months after stroke, which would have excluded most severe strokes, showed similar results to our main findings. In addition, patient and practitioner preference for statin use, which could reflect underlying health conditions associated with the risk of dementia, was not captured in the databases. While unmeasured confounding may be mitigated by an active comparator (ideally initiators of other lipid-lowering monotherapy), this was not feasible in our new-user study due to very limited number of people initiating these drugs (only 108 initiators among eligible stroke survivors, with 11 developing incident dementia during follow-up).

Second, differential misclassification of measured baseline and time-varying covariates (mainly in relation to diagnosis of comorbidities) may have also resulted in residual confounding. This misclassification was more likely to occur in statin initiators due to less contact with health services (Table 1), with unpredictable impact on our effect estimates. Similarly, as dementia was not systematically assessed in all stroke patients, misclassification can also occur in relation to the issue of underdiagnosis of dementia in clinical practice [42], in particular among statin initiators who had less contact with general practitioners (Table 1). In this case, the real association would be weaker than our estimates. However, the incidence of post-stroke dementia observed in our study was in line with that in the general population when accounting for the higher risk of dementia in stroke patients (Supplementary material of Appendix 6). As per the current consensus definition of post-stroke dementia [2], our study was focused on any dementia, without considering dementia subtype or mild cognitive impairment, which were poorly recorded in the CPRD.

Third, while censoring due to death and transfer-out has been accounted for, the association may still be overestimated to some extent due to death and transfer-out as competing events. Fourth, ideally, separate weights should be calculated for death and transfer-out as their causes might differ. However, this was not feasible due to model convergence issues. For the same reason, overall weights, rather than subgroup-specific weights, were used in the subgroup analysis, which might introduce residual confounding. Fifth, weight truncation might also lead to residual confounding [35]. However, sensitivity analysis using original weights or other truncation criteria still suggested the inverse association of statin use with dementia. Sixth, we defined statin initiation and sustained use based on statin prescriptions, which did not necessarily mean uptake of the medications. Furthermore, there were missing data for stroke subtype, BMI, and smoking. However, this limitation was unlikely to have an important impact on the results as sensitivity analyses dealing with missing data did not materially change the results.

### Comparisons with other studies and possible mechanisms

No trials have investigated the effect of statins on the risk of dementia in stroke patients [23]. Only two observational studies have reported on the association of statins with post-stroke dementia [43, 44]. In one study, the inverse association was not statistically significant (crude OR 0.89, 95% CI 0.65–1.21, with no adjusted estimate reported) [43]. The other study suggested a significant inverse association (adjusted HR 0.81, 95% CI 0.73–0.89) with adjustment for age, sex, socioeconomic status, baseline comorbidities and co-medications [44]. Our study found a stronger association of statin use with dementia. Our attempt to overcome the limitations in the previous studies, such as prevalent user bias, immortal time bias, time-varying confounding, and treatment non-persistence, could partially explain the difference in findings. While the exact mechanisms of statins on cognitive protection have not been elucidated, statins may lower the risk of post-stroke dementia by preventing recurrent stroke [45], reducing the production of amyloid-β peptides and neurofibrillary tangles [46, 47], and exerting pleiotropic effects [48].

In terms of the control outcomes, we observed a smaller protective effect on CHD risk in our ITT estimate but a similar risk in our PP analysis when compared with the RCTs [27, 28]. We did not observe an association with peptic ulcer, as would have been anticipated from the trials [14, 31, 32], suggesting the observed association of statin use with dementia would not be substantially confounded by the unmeasured factors shared with that between statin use and peptic ulcer, such as diet, alcohol consumption, gastrointestinal related drugs and gut microbiota. We found that statin use, contrary to the trial results, was associated with a reduced risk of fracture [14, 30]. The lower risk of fracture found in our study suggests the possibility of unmeasured confounding by healthy user effects or the possibility of statins reducing the risk of falls associated with fracture by preventing dementia. This inverse association with fracture was also observed in other observational studies [49], including in stroke patients (aHR 0.66, 95% CI 0.58–0.76) [50].

### Implications for practice

We found no evidence that statin use after stroke could accelerate cognitive deterioration as previous studies reported [16–20], but rather was associated with reduced risk of dementia, regardless of stroke subtype. This potential benefit might be enhanced through greater persistence with statins. This underlines the importance of interventions to tackle non-persistence, which has been recognised to compromise the effectiveness of statins in the prevention of ASCVD [6, 7]. Given the potential benefit of statins in preventing post-stroke dementia does not vary by age or cardiovascular risk factors, efforts should be made to increase uptake particularly in older patients and in those without prior cardiovascular risk factors, who have been shown to be less likely to be on statin treatment after stroke [51].

### Implications for future research

Confirmatory evidence of the effect of statins on preventing dementia is needed. The ongoing placebo-controlled trial, Pragmatic Evaluation of Events and Benefits of Lipid-Lowering in Older Adults (PREVENTABLE), assesses the overall benefits and risks of statins for preventing dementia in 20,000 adults aged 75 years or older without cardiovascular disease [52]. Such a trial would not be possible to conduct in ischaemic stroke because of the proven benefits in reducing risk of future cardiovascular events [3–5]. Since our study only provided evidence on the average estimate of association for statin use regardless of potencies and doses, further real-world evidence could illuminate whether potency, dose, and lipophilicity of statins can influence the risk of dementia following ischaemic stroke. With cumulative observational evidence on functional benefits of statins for intracerebral haemorrhage [53, 54], cognitive outcomes could be assessed in future statin trials conducted in these patients. Development and testing of interventions to enhance the use of and persistence with statins after stroke are needed.

## Conclusions

Statin initiation after stroke was associated with a lower risk of dementia, regardless of stroke subtype. This potential benefit is greater in patients persistent with statins. The observed association between statin use and post-stroke dementia may be overestimated in part due to unmeasured confounding shared with the association between statin use and fracture. Further trials with cognitive outcomes and more real-world observational evidence are needed to confirm the potential benefit of statins for preventing post-stroke dementia.

## Supplementary Information

Below is the link to the electronic supplementary material.

Supplementary file1 (PDF 1516 KB)

## Data Availability

The data that support the findings of this study are available from CPRD but restrictions apply to the availability of these data, which were used under licence for the current study, and so are not publicly available.
