# Supplementary material for: Statin use is associated with lower risk of dementia in stroke patients: a community-based cohort study with inverse probability weighted marginal structural model analysis
Source: Eur J Epidemiol. 2022 Mar 19;37(6):615–27. doi: 10.1007/s10654-022-00856-7 (PMC9288375; doi:10.1007/s10654-022-00856-7)
Supplement: Supplementary file 1 — Supplementary file1 (PDF 1516 KB) [file 10654_2022_856_MOESM1_ESM.pdf]

Appendices

Statin use is associated with lower risk of dementia in stroke patients: a community-based cohort study with marginal structural model analysis

Zhirong Yang [1], Sengwee Toh [2], Xiaojuan Li [2], Duncan Edwards [1], Carol Brayne [3], Jonathan Mant [1]

1. Primary Care Unit, Department of Public Health and Primary Care, School of Clinical Medicine, University of Cambridge, Cambridge, UK
2. Department of Population Medicine, Harvard Medical School & Harvard Pilgrim Health Care Institute, Boston, MA, USA
3. Cambridge Public Health, School of Clinical Medicine, University of Cambridge, Cambridge, UK

Appendix 1. Codes for stroke, statins, dementia and control outcomes 1

Appendix 2. Potential confounders 47

Appendix 3. Quality control criteria 49

Appendix 4. Details on effect estimation using inverse probability weighted marginal structural models 50

Appendix 5. Baseline characteristics 56

Appendix 6. Incidence of post-stroke dementia 58

Appendix 7. Distributions of weights in marginal structural models 59

Appendix 8. Sensitivity analysis 60

Appendix 1. Codes for stroke, statins, dementia and control outcomes

Read codes for stroke used in CPRD

Medcode	Read code	Description
569	G64..12	Infarction - cerebral
1298	G66..11	CVA unspecified
1469	G66..00	Stroke and cerebrovascular accident unspecified
2418	G6...00	Cerebrovascular disease
3149	G64z.00	Cerebral infarction NOS
3535	G61z.00	Intracerebral hemorrhage NOS
5051	G61..00	Intracerebral hemorrhage
5185	G64z111	Lateral medullary syndrome
5363	G64..11	CVA - cerebral artery occlusion
5602	G64z.12	Cerebellar infarction
6116	G66..13	CVA - Cerebrovascular accident unspecified
6155	G64..13	Stroke due to cerebral arterial occlusion
6253	G66..12	Stroke unspecified
6960	G61..11	CVA - cerebrovascular accid due to intracerebral hemorrhage
7780	G667.00	Left sided CVA
7912	G614.00	Pontine hemorrhage
8443	G663.00	Brain stem stroke syndrome
8837	G64..00	Cerebral arterial occlusion
9985	G64z200	Left sided cerebral infarction
10504	G64z300	Right sided cerebral infarction
12833	G668.00	Right sided CVA
13564	G613.00	Cerebellar hemorrhage
15019	G641.00	Cerebral embolism
15252	G64z.11	Brainstem infarction NOS
16517	G640.00	Cerebral thrombosis
16956	G669.00	Cerebral palsy, not congenital or infantile, acute
17322	G664.00	Cerebellar stroke syndrome
18604	G61..12	Stroke due to intracerebral hemorrhage
18689	G660.00	Middle cerebral artery syndrome
19201	G61X100	Right sided intracerebral hemorrhage, unspecified
19260	G662.00	Posterior cerebral artery syndrome
19280	G661.00	Anterior cerebral artery syndrome
23671	G63y000	Cerebral infarct due to thrombosis of precerebral arteries
24446	G63y100	Cerebral infarction due to embolism of precerebral arteries
25615	G64z000	Brainstem infarction
26424	G64z400	Infarction of basal ganglia
27975	G641000	Cerebral infarction due to embolism of cerebral arteries
28314	G61X000	Left sided intracerebral hemorrhage, unspecified
30045	G616.00	External capsule hemorrhage
30202	G617.00	Intracerebral hemorrhage, intraventricular
31060	G61X.00	Intracerebral hemorrhage in hemisphere, unspecified
31595	G610.00	Cortical hemorrhage
33499	G665.00	Pure motor lacunar syndrome

Medcode	Read code	Description
33543	G6X..00	Cerebrl infarctn due/unspcf occlusn or sten/cerebrl artrs
34758	G641.11	Cerebral embolus
36717	G640000	Cerebral infarction due to thrombosis of cerebral arteries
39344	G676000	Cereb infarct due cerebral venous thrombosis, nonpyogenic
40338	G611.00	Internal capsule hemorrhage
40758	G6W..00	Cereb infarct due unsp occlus/stenos precerebr arteries
44765	G653.00	Carotid artery syndrome hemispheric
46316	G612.00	Basal nucleus hemorrhage
47642	G64z100	Wallenberg syndrome
50594	G654.00	Multiple and bilateral precerebral artery syndromes
51767	G666.00	Pure sensory lacunar syndrome
53745	Gyu6400	[X]Other cerebral infarction
55247	G65z000	Impending cerebral ischaemia
57315	G618.00	Intracerebral hemorrhage, multiple localised
62342	G615.00	Bulbar hemorrhage
90572	Gyu6500	[X]Occlusion and stenosis of other precerebral arteries
91627	Gyu6300	[X]Cerebrl infarctn due/unspcf occlusn or sten/cerebrl artrs
92036	Gyu6600	[X]Occlusion and stenosis of other cerebral arteries
94482	Gyu6G00	[X]Cereb infarct due unsp occlus/stenos precerebr arteries
96630	Gyu6F00	[X]Intracerebral hemorrhage in hemisphere, unspecified

ICD-10 Codes for stroke used in HES and ONS

ICD Code	Description
I63	Cerebral infarction
I63.0	Cerebral infarction due to thrombosis of precerebral arteries
I63.1	Cerebral infarction due to embolism of precerebral arteries
I63.2	Cerebral infarction due to unspecified occlusion or stenosis of precerebral arteries
I63.3	Cerebral infarction due to thrombosis of cerebral arteries
I63.4	Cerebral infarction due to embolism of cerebral arteries
I63.5	Cerebral infarction due to unspecified occlusion or stenosis of cerebral arteries
I63.6	Cerebral infarction due to cerebral venous thrombosis, nonpyogenic
I63.8	Other cerebral infarction
I63.9	Cerebral infarction, unspecified
I64	Stroke, not specified as hemorrhage or infarction
I64.0	Stroke, not specified as hemorrhage or infarction
I61	Intracerebral hemorrhage
I61.0	Intracerebral hemorrhage in hemisphere, subcortical
I61.1	Intracerebral hemorrhage in hemisphere, cortical
I61.2	Intracerebral hemorrhage in hemisphere, unspecified
I61.3	Intracerebral hemorrhage in brain stem
I61.4	Intracerebral hemorrhage in cerebellum
I61.5	Intracerebral hemorrhage, intraventricular
I61.6	Intracerebral hemorrhage, multiple localised
I61.8	Other intracerebral hemorrhage
I61.9	Intracerebral hemorrhage, unspecified

BNF Codes for statins

Prodcode	Gemscriptcode	BNF code	Description
25	72488020	2120400	Simvastatin 20mg tablets
28	83943020	2120400	Atorvastatin 10mg tablets
42	72487020	2120400	Simvastatin 10mg tablets
51	72489020	2120400	Simvastatin 40mg tablets
75	83944020	2120400	Atorvastatin 20mg tablets
379	75344020	2120400	Fluvastatin 20mg capsules
420	84278020	0000000	Cerivastatin 100microgram tablets
490	73782020	2120400	Pravastatin 10mg tablets
713	85632020	2120400	Rosuvastatin 10mg tablets
730	73783020	2120400	Pravastatin 20mg tablets
745	83945020	2120400	Atorvastatin 40mg tablets
802	87029020	2120400	Simvador 40mg tablets (Discovery Pharmaceuticals)
818	90773020	2120400	Simvastatin 20mg/5ml oral solution sugar free
1219	73784020	2120400	Pravastatin 40mg tablets
1221	73787020	2120400	Lipostat 10mg tablets (Bristol-Myers Squibb Pharmaceuticals
1223	73789020	2120400	Lipostat 40mg tablets (Bristol-Myers Squibb Pharmaceuticals
2137	75345020	2120400	Fluvastatin 40mg capsules
2718	72482020	2120400	Zocor 10mg tablets (Merck Sharp & Dohme Ltd)
2955	83955020	2120400	Lipitor 40mg tablets (Pfizer Ltd)
3411	83953020	2120400	Lipitor 10mg tablets (Pfizer Ltd)
3690	73788020	2120400	Lipostat 20mg tablets (Bristol-Myers Squibb Pharmaceuticals
4961	84284020	2120400	Lipobay 300microgram Tablet (Bayer Plc)
5009	84279020	0000000	Cerivastatin 200microgram tablets
5148	77174020	2120400	Simvastatin 80mg tablets
5251	84280020	0000000	Cerivastatin 300microgram tablets
5278	76527020	0000000	Cerivastatin 400microgram tablets
5775	81727020	2120400	Atorvastatin 80mg tablets
5985	79882020	2120400	Lescol XL 80mg tablets (Novartis Pharmaceuticals UK Ltd)
6168	72484020	2120400	Zocor 40mg tablets (Merck Sharp & Dohme Ltd)
6213	80350020	2120400	Rosuvastatin 20mg tablets
7196	72483020	2120400	Zocor 20mg tablets (Merck Sharp & Dohme Ltd)
7347	76621020	2120400	Crestor 10mg tablets (AstraZeneca UK Ltd)
7374	83954020	2120400	Lipitor 20mg tablets (Pfizer Ltd)
7552	89242020	02120200/02120400	Simvastatin 20mg / Ezetimibe 10mg tablets
7554	89881020	2120400	Rosuvastatin 5mg tablets
8380	75340020	2120400	Lescol 20mg capsules (Novartis Pharmaceuticals UK Ltd)
9153	75341020	2120400	Lescol 40mg capsules (Novartis Pharmaceuticals UK Ltd)
9315	84282020	2120400	Lipobay 100microgram Tablet (Bayer Plc)
9316	84283020	2120400	Lipobay 200microgram Tablet (Bayer Plc)
9897	76618020	2120400	Rosuvastatin 40mg tablets
9920	87027020	2120400	Simvador 20mg tablets (Discovery Pharmaceuticals)
9930	76259020	2120400	Crestor 40mg tablets (AstraZeneca UK Ltd)
10172	89246020	02120200/02120400	Simvastatin 40mg / Ezetimibe 10mg tablets
10183	89260020	02120200/02120400	Simvastatin 40mg with ezetimibe 10mg tablet
10206	89262020	02120200/02120400	Simvastatin 80mg with ezetimibe 10mg tablet
11627	75346020	2120400	Fluvastatin 80mg modified-release tablets
11815	89258020	02120200/02120400	Simvastatin 20mg with ezetimibe 10mg tablet

Prodcode	Gemscriptcode	BNF code	Description
13041	87025020	2120400	Simvador 10mg tablets (Discovery Pharmaceuticals)
14219	89250020	02120200/02120400	Simvastatin 80mg / Ezetimibe 10mg tablets
15252	76624020	2120400	Crestor 20mg tablets (AstraZeneca UK Ltd)
16186	89255020	02120200/02120400	Inegy 10mg/80mg tablets (Merck Sharp & Dohme Ltd)
17059	89248020	02120200/02120400	Inegy 10mg/40mg tablets (Merck Sharp & Dohme Ltd)
17683	81729020	2120400	Lipitor 80mg tablets (Pfizer Ltd)
17688	89883020	2120400	Crestor 5mg tablets (AstraZeneca UK Ltd)
18442	76531020	2120400	Lipobay 400microgram Tablet (Bayer Plc)
21020	89244020	02120200/02120400	Inegy 10mg/20mg tablets (Merck Sharp & Dohme Ltd)
22579	76460020	2120400	Zocor 80mg tablets (Merck Sharp & Dohme Ltd)
24509	!6579801	0000000	SIMVASTATIN
29438	!6579802	0000000	SIMVASTATIN
31658	76528020	0000000	Cerivastatin 800microgram tablets
31930	88103020	2120400	Zocor heart-pro 10mg Tablet (McNeil Products Ltd)
32909	65854020	2120400	Simvastatin 80mg tablets (A A H Pharmaceuticals Ltd)
32921	69857020	2120400	Pravastatin 10mg Tablet (Dr Reddy's Laboratories (UK) Ltd)
33082	65848020	2120400	Simvastatin 20mg tablets (A A H Pharmaceuticals Ltd)
34312	66042020	2120400	Simvastatin 20mg tablets (Mylan)
34316	65879020	2120400	Simvastatin 20mg tablets (Teva UK Ltd)
34353	66045020	2120400	Simvastatin 40mg tablets (Mylan)
34366	65924020	2120400	Simvastatin 20mg tablets (IVAX Pharmaceuticals UK Ltd)
34376	65883020	2120400	Simvastatin 40mg tablets (Teva UK Ltd)
34381	65927020	2120400	Simvastatin 40mg tablets (IVAX Pharmaceuticals UK Ltd)
34476	65672020	2120400	Simvastatin 20mg Tablet (Ratiopharm UK Ltd)
34481	65920020	2120400	Simvastatin 10mg tablets (IVAX Pharmaceuticals UK Ltd)
34502	65851020	2120400	Simvastatin 40mg tablets (A A H Pharmaceuticals Ltd)
34535	66039020	2120400	Simvastatin 10mg tablets (Mylan)
34545	65677020	2120400	Simvastatin 40mg Tablet (Ratiopharm UK Ltd)
34560	65668020	2120400	Simvastatin 10mg Tablet (Ratiopharm UK Ltd)
34746	65949020	2120400	Simvastatin 20mg Tablet (Niche Generics Ltd)
34814	65905020	2120400	Simvastatin 20mg tablets (Wockhardt UK Ltd)
34820	68205020	2120400	Pravastatin 40mg tablets (A A H Pharmaceuticals Ltd)
34879	65952020	2120400	Simvastatin 40mg Tablet (Niche Generics Ltd)
34891	65962020	2120400	Simvastatin 20mg tablets (Kent Pharmaceuticals Ltd)
34907	65910020	2120400	Simvastatin 40mg tablets (Wockhardt UK Ltd)
34955	65829020	2120400	Simvastatin 10mg tablets (A A H Pharmaceuticals Ltd)
34969	65939020	2120400	Simvastatin 40mg tablets (Actavis UK Ltd)
36377	68275020	2120400	Pravastatin 20mg tablets (Teva UK Ltd)
37434	66208020	2120400	Simvastatin 40mg tablets (Sandoz Ltd)
39060	66656020	2120400	Simvastatin 20mg tablets (Dexcel-Pharma Ltd)
39652	96566020	2120400	Simvastatin 40mg/5ml oral solution sugar free
39675	72886020	2120400	Simvastatin 20mg/5ml Oral suspension (Martindale [illegible]
39870	96704020	2120400	Simvador 80mg tablets (Discovery Pharmaceuticals)
40340	65874020	2120400	Simvastatin 10mg tablets (Teva UK Ltd)
40382	68201020	2120400	Pravastatin 20mg tablets (A A H Pharmaceuticals Ltd)
40601	69804020	2120400	Simvastatin 20mg tablets (Ranbaxy (UK) Ltd)
41657	66993020	2120400	Simvastatin 80mg tablets (Teva UK Ltd)
43218	68272020	2120400	Pravastatin 10mg tablets (Teva UK Ltd)

Prodcode	Gemscriptcode	BNF code	Description
44528	77364020	2120400	Simvastatin 20mg/5ml oral suspension sugar free (Rosemont [illegible]
44650	66659020	2120400	Simvastatin 40mg tablets (Dexcel-Pharma Ltd)
44878	88014020	2120400	Ranzolont 10mg tablets (Ranbaxy (UK) Ltd)
45219	65967020	2120400	Simvastatin 40mg tablets (Kent Pharmaceuticals Ltd)
45235	66205020	2120400	Simvastatin 20mg tablets (Sandoz Ltd)
45245	65935020	2120400	Simvastatin 20mg tablets (Actavis UK Ltd)
45346	66066020	2120400	Simvastatin 40mg tablets (Arrow Generics Ltd)
46878	71629020	2120400	Simvastatin 40mg tablets (Almus Pharmaceuticals Ltd)
46956	66069020	2120400	Simvastatin 80mg tablets (Arrow Generics Ltd)
47065	573021	0000000	Atorvastatin 20mg chewable tablets sugar free
47090	569021	0000000	Atorvastatin 10mg chewable tablets sugar free
47630	575021	0000000	Lipitor 20mg chewable tablets (Pfizer Ltd)
47721	571021	2120400	Lipitor 10mg chewable tablets (Pfizer Ltd)
47774	66058020	2120400	Simvastatin 10mg tablets (Arrow Generics Ltd)
47948	66323020	2120400	Simvastatin 10mg tablets (Tillomed Laboratories Ltd)
47988	68735020	2120400	Pravastatin 40mg tablets (Mylan)
48018	66063020	2120400	Simvastatin 20mg tablets (Arrow Generics Ltd)
48051	65957020	2120400	Simvastatin 10mg tablets (Kent Pharmaceuticals Ltd)
48058	69800020	2120400	Simvastatin 10mg tablets (Ranbaxy (UK) Ltd)
48078	65932020	2120400	Simvastatin 10mg tablets (Actavis UK Ltd)
48097	68278020	2120400	Pravastatin 40mg tablets (Teva UK Ltd)
48221	37402020	2120400	Simvastatin 20mg/5ml oral suspension sugar free
48346	41848020	2120400	Atorvastatin 60mg tablets
48431	37429020	2120400	Simvastatin 40mg/5ml oral suspension sugar free
48518	35411020	2120400	Atorvastatin 10mg/5ml oral solution
48867	2512020	2120400	Simvastatin 40mg tablets (Alliance Healthcare (Distribution) Ltd)
48973	41846020	2120400	Atorvastatin 30mg tablets
49061	2518020	2120400	Simvastatin 40mg tablets (Bristol Laboratories Ltd)
49062	2496020	2120400	Simvastatin 20mg tablets (Alliance Healthcare (Distribution) Ltd)
49558	41345020	2120400	Atorvastatin 20mg tablets (A A H Pharmaceuticals Ltd)
49587	71634020	2120400	Simvastatin 80mg tablets (Almus Pharmaceuticals Ltd)
49751	41330020	2120400	Atorvastatin 40mg tablets (Alliance Healthcare (Distribution) [illegible]
50236	41912020	2120400	Atorvastatin 10mg tablets (Zentiva)
50272	42161020	2120400	Atorvastatin 40mg tablets (Pfizer Ltd)
50483	2514020	2120400	Simvastatin 40mg tablets (Relonchem Ltd)
50564	2501020	2120400	Simvastatin 20mg tablets (Relonchem Ltd)
50670	2524020	2120400	Simvastatin 40mg tablets (Milpharm Ltd)
50703	2523020	2120400	Simvastatin 40mg tablets (Accord Healthcare Ltd)
50754	38510020	2120400	Simvastatin 20mg tablets (Medreich Plc)
50788	42167020	2120400	Atorvastatin 20mg tablets (Pfizer Ltd)
50790	41343020	2120400	Atorvastatin 20mg tablets (Dexcel-Pharma Ltd)
50882	2515020	2120400	Simvastatin 40mg tablets (Somex Pharma)
50925	2542020	2120400	Pravastatin 10mg tablets (Sigma Pharmaceuticals Plc)
50963	41328020	2120400	Atorvastatin 40mg tablets (Teva UK Ltd)
51085	38509020	2120400	Simvastatin 10mg tablets (Medreich Plc)
51134	41338020	2120400	Atorvastatin 10mg tablets (A A H Pharmaceuticals Ltd)
51166	38511020	2120400	Simvastatin 40mg tablets (Medreich Plc)
51200	41905020	2120400	Atorvastatin 40mg tablets (Arrow Generics Ltd)

Prodcode	Gemscriptcode	BNF code	Description
51233	2479020	2120400	Simvastatin 10mg tablets (Alliance Healthcare (Distribution) Ltd)
51359	41913020	2120400	Atorvastatin 20mg tablets (Arrow Generics Ltd)
51483	2511020	2120400	Simvastatin 20mg tablets (Milpharm Ltd)
51622	41349020	2120400	Atorvastatin 20mg tablets (Consilient Health Ltd)
51676	38515020	2120400	Pravastatin 40mg tablets (Medreich Plc)
51715	2489020	2120400	Simvastatin 10mg tablets (Sigma Pharmaceuticals Plc)
51876	41331020	2120400	Atorvastatin 40mg tablets (Consilient Health Ltd)
51890	38514020	2120400	Pravastatin 20mg tablets (Medreich Plc)
52097	41906020	2120400	Atorvastatin 40mg tablets (Wockhardt UK Ltd)
52098	69807020	2120400	Simvastatin 40mg tablets (Ranbaxy (UK) Ltd)
52168	41347020	2120400	Atorvastatin 20mg tablets (Aspire Pharma Ltd)
52211	41344020	2120400	Atorvastatin 20mg tablets (Actavis UK Ltd)
52257	2510020	2120400	Simvastatin 20mg tablets (Accord Healthcare Ltd)
52397	43826020	2120400	Atorvastatin 40mg tablets (Dr Reddy's Laboratories (UK) Ltd)
52398	41327020	2120400	Atorvastatin 40mg tablets (A A H Pharmaceuticals Ltd)
52459	41288020	2120400	Atorvastatin 80mg tablets (Actavis UK Ltd)
52460	41329020	2120400	Atorvastatin 40mg tablets (Aspire Pharma Ltd)
52625	65902020	2120400	Simvastatin 10mg tablets (Wockhardt UK Ltd)
52676	20743020	2120400	Simvastatin 10mg/5ml oral suspension
52755	2548020	2120400	Pravastatin 20mg tablets (Alliance Healthcare (Distribution) Ltd)
52812	2505020	2120400	Simvastatin 20mg tablets (Sigma Pharmaceuticals Plc)
52821	43806020	2120400	Atorvastatin 80mg tablets (Dr Reddy's Laboratories (UK) Ltd)
52953	2506020	2120400	Simvastatin 20mg tablets (Bristol Laboratories Ltd)
52962	38512020	2120400	Simvastatin 80mg tablets (Medreich Plc)
53087	2502020	2120400	Simvastatin 20mg tablets (Somex Pharma)
53340	2519020	2120400	Zocor 40mg tablets (Lexon (UK) Ltd)
53415	2495020	2120400	Simvastatin 10mg tablets (Milpharm Ltd)
53460	10669020	2120400	Crestor 10mg tablets (DE Pharmaceuticals)
53594	42020	2120400	Lipitor 80mg tablets (Mawdsley-Brooks & Company Ltd)
53676	66326020	2120400	Simvastatin 20mg tablets (Tillomed Laboratories Ltd)
53770	75882020	2120400	Fluvastatin 40mg capsules (A A H Pharmaceuticals Ltd)
53772	41292020	2120400	Atorvastatin 80mg tablets (Alliance Healthcare (Distribution)
53813	2294020	0000000	Lipobay 100microgram tablets (Bayer Plc)
53822	2490020	2120400	Simvastatin 10mg tablets (Bristol Laboratories Ltd)
53887	41326020	2120400	Atorvastatin 40mg tablets (Actavis UK Ltd)
53890	42148020	2120400	Atorvastatin 80mg tablets (Pfizer Ltd)
53908	66652020	2120400	Simvastatin 10mg tablets (Dexcel-Pharma Ltd)
53966	2521020	2120400	Simvastatin 40mg tablets (Phoenix Healthcare Distribution Ltd)
54240	2517020	2120400	Simvastatin 40mg tablets (Sigma Pharmaceuticals Plc)
54266	20745020	2120400	Simvastatin 20mg/5ml oral suspension
54435	70097020	2120400	Pravastatin 40mg tablets (Almus Pharmaceuticals Ltd)
54493	2485020	2120400	Simvastatin 10mg tablets (Relonchem Ltd)
54535	42166020	2120400	Atorvastatin 10mg tablets (Pfizer Ltd)
54606	78017020	2120400	Simvastatin 20mg/5ml oral suspension sugar free (A A H
54607	70094020	2120400	Pravastatin 20mg tablets (Almus Pharmaceuticals Ltd)
54655	2494020	2120400	Simvastatin 10mg tablets (Accord Healthcare Ltd)
54819	77492020	2120400	Simvastatin 40mg/5ml oral suspension sugar free (Rosemont
54947	71626020	2120400	Simvastatin 20mg tablets (Almus Pharmaceuticals Ltd)

Prodcode	Gemscriptcode	BNF code	Description
54976	2486020	2120400	Simvastatin 10mg tablets (Somex Pharma)
54985	35160020	2120400	Simvastatin 40mg/5ml oral suspension
54992	35413020	2120400	Atorvastatin 10mg/5ml oral suspension
55032	41336020	2120400	Atorvastatin 10mg tablets (Dexcel-Pharma Ltd)
55034	35417020	2120400	Atorvastatin 40mg/5ml oral suspension
55207	2387020	0000000	Lipobay 200microgram tablets (Bayer Plc)
55444	41907020	2120400	Atorvastatin 40mg tablets (Zentiva)
55452	2508020	2120400	Simvastatin 20mg tablets (Phoenix Healthcare Distribution Ltd)
55727	41337020	2120400	Atorvastatin 10mg tablets (Actavis UK Ltd)
55912	2557020	2120400	Pravastatin 40mg tablets (Alliance Healthcare (Distribution) Ltd)
56016	38787020	2120400	Lipitor 20mg chewable tablets (Pfizer Ltd)
56065	47853020	2120400	Simvastatin 20mg/5ml oral suspension sugar free (Waymade [illegible]
56097	38784020	2120400	Atorvastatin 10mg chewable tablets sugar free
56146	47203020	2120400	Pravastatin 10mg tablets (Waymade Healthcare Plc)
56165	38786020	2120400	Atorvastatin 20mg chewable tablets sugar free
56182	41891020	2120400	Atorvastatin 80mg tablets (Zentiva)
56248	2618020	2120400	Atorvastatin 20mg tablets (Sigma Pharmaceuticals Plc)
56481	2488020	2120400	Zocor 10mg tablets (Sigma Pharmaceuticals Plc)
56494	2504020	2120400	Zocor 20mg tablets (Sigma Pharmaceuticals Plc)
56564	47180020	2120400	Atorvastatin 20mg tablets (Almus Pharmaceuticals Ltd)
56607	47204020	2120400	Pravastatin 20mg tablets (Waymade Healthcare Plc)
56735	68731020	2120400	Pravastatin 20mg tablets (Mylan)
56841	41325020	2120400	Atorvastatin 40mg tablets (Dexcel-Pharma Ltd)
56893	2564020	2120400	Pravastatin 40mg tablets (Accord Healthcare Ltd)
56916	68450020	2120400	Pravastatin 40mg tablets (PLIVA Pharma Ltd)
57108	47205020	2120400	Pravastatin 40mg tablets (Waymade Healthcare Plc)
57117	47030020	2120400	Atorvastatin 80mg tablets (Waymade Healthcare Plc)
57137	70090020	2120400	Pravastatin 10mg tablets (Almus Pharmaceuticals Ltd)
57296	2553020	2120400	Pravastatin 20mg tablets (Phoenix Healthcare Distribution Ltd)
57329	36882020	2120400	Simvastatin 25mg/5ml oral suspension
57348	41342020	2120400	Atorvastatin 10mg tablets (Consilient Health Ltd)
57397	2544020	2120400	Pravastatin 10mg tablets (Accord Healthcare Ltd)
57568	2491020	2120400	Zocor 10mg tablets (Lexon (UK) Ltd)
57763	10666020	2120400	Rosuvastatin 10mg tablets (Waymade Healthcare Plc)
57834	16680021	2120400	Atorvastatin 40mg tablets (DE Pharmaceuticals)
57836	41290020	2120400	Atorvastatin 80mg tablets (Teva UK Ltd)
57999	10641020	2120400	Crestor 40mg tablets (Lexon (UK) Ltd)
58041	41346020	2120400	Atorvastatin 20mg tablets (Teva UK Ltd)
58110	41915020	2120400	Atorvastatin 20mg tablets (Zentiva)
58315	47200020	2120400	Simvastatin 20mg tablets (Waymade Healthcare Plc)
58394	41348020	2120400	Atorvastatin 20mg tablets (Alliance Healthcare (Distribution) [illegible]
58418	41289020	2120400	Atorvastatin 80mg tablets (A A H Pharmaceuticals Ltd)
58480	2622020	0000000	Lipobay 300microgram tablets (Bayer Plc)
58617	36834020	2120400	Rosuvastatin 20mg/5ml oral suspension
58742	41889020	2120400	Atorvastatin 80mg tablets (Arrow Generics Ltd)
58755	2492020	2120400	Simvastatin 10mg tablets (Phoenix Healthcare Distribution Ltd)
58834	2603020	2120400	Atorvastatin 10mg tablets (DE Pharmaceuticals)
58868	2605020	2120400	Atorvastatin 10mg tablets (Sigma Pharmaceuticals Plc)

Prodcode	Gemscriptcode	BNF code	Description
59272	47179020	2120400	Atorvastatin 20mg tablets (Waymade Healthcare Plc)
59278	75832020	2120400	Fluvastatin 20mg capsules (Zentiva)
59331	2604020	2120400	Lipitor 10mg tablets (DE Pharmaceuticals)
59357	17406021	2120400	Atorvastatin 10mg tablets (Ranbaxy (UK) Ltd)
59446	47193020	2120400	Atorvastatin 40mg tablets (Almus Pharmaceuticals Ltd)
59447	10674020	2120400	Crestor 20mg tablets (Waymade Healthcare Plc)
59452	10781020	2120400	Rosuvastatin 5mg tablets (Waymade Healthcare Plc)
59508	2554020	2120400	Pravastatin 20mg tablets (Accord Healthcare Ltd)
59776	41291020	2120400	Atorvastatin 80mg tablets (Aspire Pharma Ltd)
59859	41339020	2120400	Atorvastatin 10mg tablets (Teva UK Ltd)
60160	10787020	2120400	Rosuvastatin 5mg tablets (Mawdsley-Brooks & Company Ltd)
60251	68335020	2120400	Pravastatin 10mg tablets (Sandoz Ltd)
60464	35365020	2120400	Atorvastatin 20mg/5ml oral suspension
60511	17409021	2120400	Atorvastatin 40mg tablets (Ranbaxy (UK) Ltd)
60607	16641021	2120400	Atorvastatin 80mg tablets (DE Pharmaceuticals)
60989	14765021	2120400	Atorvastatin 80mg tablets (Phoenix Healthcare Distribution Ltd)
61134	2552020	2120400	Pravastatin 20mg tablets (Sigma Pharmaceuticals Plc)
61149	47177020	2120400	Atorvastatin 10mg tablets (Waymade Healthcare Plc)
61155	78014020	2120400	Simvastatin 40mg/5ml oral suspension sugar free (A A H
61321	66197020	2120400	Simvastatin 10mg tablets (Sandoz Ltd)
61360	71623020	2120400	Simvastatin 10mg tablets (Almus Pharmaceuticals Ltd)
61665	47199020	2120400	Simvastatin 10mg tablets (Waymade Healthcare Plc)
62132	2623020	0000000	Lipobay 400microgram tablets (Bayer Plc)
62137	47201020	2120400	Simvastatin 40mg tablets (Waymade Healthcare Plc)
62148	77027020	2120400	Fluvastatin 20mg capsules (Actavis UK Ltd)
62219	16676021	2120400	Atorvastatin 20mg tablets (DE Pharmaceuticals)
62429	2616020	2120400	Atorvastatin 20mg tablets (DE Pharmaceuticals)
62476	47031020	2120400	Atorvastatin 80mg tablets (Almus Pharmaceuticals Ltd)
62979	2558020	2120400	Pravastatin 40mg tablets (Kent Pharmaceuticals Ltd)
63074	68446020	2120400	Pravastatin 20mg tablets (PLIVA Pharma Ltd)
63140	41341020	2120400	Atorvastatin 10mg tablets (Alliance Healthcare (Distribution)
63249	41293020	2120400	Atorvastatin 80mg tablets (Consilient Health Ltd)
63469	41847020	2120400	Atorvastatin 30mg tablets (Consilient Health Ltd)
63787	2539020	2120400	Pravastatin 10mg tablets (Tillomed Laboratories Ltd)
64067	35363020	2120400	Atorvastatin 20mg/5ml oral solution
64104	47996021	2120400	Simvastatin 20mg tablets (Crescent Pharma Ltd)
64180	47995021	2120400	Simvastatin 10mg tablets (Crescent Pharma Ltd)
64307	47997021	2120400	Simvastatin 40mg tablets (Crescent Pharma Ltd)
64702	44117020	2120400	Atorvastatin 30mg tablets (A A H Pharmaceuticals Ltd)
64810	14786021	2120400	Atorvastatin 40mg tablets (Phoenix Healthcare Distribution Ltd)
64825	14784021	2120400	Atorvastatin 10mg tablets (Phoenix Healthcare Distribution Ltd)
64868	52201021	2120400	Atorvastatin 40mg tablets (Sigma Pharmaceuticals Plc)
64968	53343021	2120400	Simvastatin 10mg tablets (DE Pharmaceuticals)
65181	53345021	2120400	Simvastatin 40mg tablets (DE Pharmaceuticals)
65193	17407021	2120400	Atorvastatin 20mg tablets (Ranbaxy (UK) Ltd)
65679	53344021	2120400	Simvastatin 20mg tablets (DE Pharmaceuticals)
65901	73837020	2120400	Simvastatin 40mg tablets (Zentiva)
65925	37403020	2120400	Simvastatin 20mg/5ml oral suspension sugar free (Alliance

Prodcode	Gemscriptcode	BNF code	Description
66505	65936021	02120300/02120400	Fenofibrate 145mg / Simvastatin 40mg tablets
66780	65934021	02120300/02120400	Fenofibrate 145mg / Simvastatin 20mg tablets
66963	52136021	2120400	Atorvastatin 80mg tablets (Sigma Pharmaceuticals Plc)
67098	66010021	2120400	Simvastatin 10mg tablets (Brown & Burk UK Ltd)
67328	2596020	2120400	Lescol XL 80mg tablets (Mawdsley-Brooks & Company Ltd)
67402	62259021	2120400	Atorvastatin 40mg tablets (Kent Pharmaceuticals Ltd)
67573	16674021	2120400	Atorvastatin 10mg tablets (DE Pharmaceuticals)
67660	17393021	2120400	Atorvastatin 80mg tablets (Ranbaxy (UK) Ltd)
67745	73830020	2120400	Simvastatin 10mg tablets (Zentiva)
67773	73834020	2120400	Simvastatin 20mg tablets (Zentiva)
67829	68340020	2120400	Pravastatin 20mg tablets (Sandoz Ltd)
67846	47178020	2120400	Atorvastatin 10mg tablets (Almus Pharmaceuticals Ltd)
68023	41340020	2120400	Atorvastatin 10mg tablets (Aspire Pharma Ltd)
68048	14785021	2120400	Atorvastatin 20mg tablets (Phoenix Healthcare Distribution Ltd)
68156	68196020	2120400	Pravastatin 10mg tablets (A A H Pharmaceuticals Ltd)
68467	62258021	2120400	Atorvastatin 20mg tablets (Kent Pharmaceuticals Ltd)
68563	66012021	2120400	Simvastatin 40mg tablets (Brown & Burk UK Ltd)
68686	70624021	2120400	Simvastatin 20mg tablets (Genesis Pharmaceuticals Ltd)
68785	70625021	2120400	Atorvastatin 10mg tablets (Mylan)
68827	70626021	2120400	Atorvastatin 20mg tablets (Mylan)
69093	41890020	2120400	Atorvastatin 80mg tablets (Wockhardt UK Ltd)
69413	66011021	2120400	Simvastatin 20mg tablets (Brown & Burk UK Ltd)
69427	70627021	2120400	Atorvastatin 40mg tablets (Mylan)
69528	65935021	02120300/02120400	Cholib 145mg/20mg tablets (Mylan)
70308	10678020	2120400	Crestor 20mg tablets (Sigma Pharmaceuticals Plc)
70486	65937021	02120300/02120400	Cholib 145mg/40mg tablets (Mylan)
70693	52199021	2120400	Atorvastatin 10mg tablets (Sigma Pharmaceuticals Plc)
70987	43827020	2120400	Atorvastatin 10mg tablets (Dr Reddy's Laboratories (UK) Ltd)
71014	10675020	2120400	Rosuvastatin 20mg tablets (Waymade Healthcare Plc)
71015	38513020	2120400	Pravastatin 10mg tablets (Medreich Plc)
71017	43828020	2120400	Atorvastatin 20mg tablets (Dr Reddy's Laboratories (UK) Ltd)
71029	40520020	2120400	Fluvastatin 40mg capsules (Sandoz Ltd)
71773	66013021	2120400	Simvastatin 80mg tablets (Brown & Burk UK Ltd)
72048	68377020	2120400	Pravastatin 40mg tablets (Actavis UK Ltd)
72050	71081021	2120400	Simvastatin 10mg tablets (Genesis Pharmaceuticals Ltd)
72149	35881020	2120400	Pravastatin 5mg/5ml oral suspension
72164	78966021	2120400	Atorvastatin 20mg tablets (Bristol Laboratories Ltd)
72213	41914020	2120400	Atorvastatin 20mg tablets (Wockhardt UK Ltd)
72308	2573020	2120400	Fluvastatin 20mg capsules (Alliance Healthcare (Distribution)

Abbreviation: BNF, British National Formulary.

Read codes for dementia used in CPRD

Medcode	Read code	Description
1350	E00..12	Senile/presenile dementia
1916	E00..11	Senile dementia
1917	F110.00	Alzheimer's disease
4357	Eu02z14	[X] Senile dementia NOS
4693	Eu02z00	[X] Unspecified dementia
5931	1461.00	H/O: dementia
6578	Eu01.00	[X]Vascular dementia
7323	E000.00	Uncomplicated senile dementia
7572	F116.00	Lewy body disease
7664	Eu00.00	[X]Dementia in Alzheimer's disease
8195	Eu00z11	[X]Alzheimer's dementia unspec
8634	E004.11	Multi infarct dementia
8934	Eu01200	[X]Subcortical vascular dementia
9509	Eu02300	[X]Dementia in Parkinson's disease
9565	Eu01.11	[X]Arteriosclerotic dementia
11136	F111.00	Pick's disease
11175	Eu01100	[X]Multi-infarct dementia
11379	Eu00112	[X]Senile dementia,Alzheimer's type
12621	Eu02.00	[X]Dementia in other diseases classified elsewhere
15165	E001.00	Presenile dementia
16797	F110000	Alzheimer's disease with early onset
18386	E002000	Senile dementia with paranoia
19393	Eu01z00	[X]Vascular dementia, unspecified
19477	E004.00	Arteriosclerotic dementia
21887	E002100	Senile dementia with depression
25386	E041.00	Dementia in conditions EC
25704	Eu00011	[X]Presenile dementia,Alzheimer's type
26270	Eu02500	[X]Lewy body dementia
26323	Eu10711	[X]Alcoholic dementia NOS
27342	E012.11	Alcoholic dementia NOS
27677	E001300	Presenile dementia with depression
27759	Eu02z16	[X] Senile dementia, depressed or paranoid type
28402	Eu02000	[X]Dementia in Pick's disease
29386	Eu00z00	[X]Dementia in Alzheimer's disease, unspecified
30032	E001200	Presenile dementia with paranoia
30706	Eu00200	[X]Dementia in Alzheimer's dis, atypical or mixed type
31016	Eu01300	[X]Mixed cortical and subcortical vascular dementia
32057	F110100	Alzheimer's disease with late onset
33707	E00..00	Senile and presenile organic psychotic conditions
34944	Eu02z13	[X] Primary degenerative dementia NOS
37014	Eu02200	[X]Dementia in Huntington's disease
37015	E003.00	Senile dementia with delirium
38438	E001z00	Presenile dementia NOS
38678	Eu00100	[X]Dementia in Alzheimer's disease with late onset
41089	E002z00	Senile dementia with depressive or paranoid features NOS
41185	Eu02400	[X]Dementia in human immunodef virus [HIV] disease
42279	E004z00	Arteriosclerotic dementia NOS
42602	E001000	Uncomplicated presenile dementia
43089	E004000	Uncomplicated arteriosclerotic dementia
43292	E004300	Arteriosclerotic dementia with depression
43346	Eu00113	[X]Primary degen dementia of Alzheimer's type, senile onset

Medcode	Read code	Description
44674	E002.00	Senile dementia with depressive or paranoid features
46488	Eu01000	[X]Vascular dementia of acute onset
46762	Eu00111	[X]Alzheimer's disease type 1
48501	Eu02z11	[X] Presenile dementia NOS
49263	Eu00000	[X]Dementia in Alzheimer's disease with early onset
49513	E001100	Presenile dementia with delirium
53446	Eu04100	[X]Delirium superimposed on dementia
54106	Eu02100	[X]Dementia in Creutzfeldt-Jakob disease
54505	E012.00	Other alcoholic dementia
55313	Eu01y00	[X]Other vascular dementia
55467	E004200	Arteriosclerotic dementia with paranoia
55838	Eu01111	[X]Predominantly cortical dementia
56912	E004100	Arteriosclerotic dementia with delirium
59122	Fyu3000	[X]Other Alzheimer's disease
60059	Eu00012	[X]Primary degen dementia, Alzheimer's type, presenile onset
61528	Eu00013	[X]Alzheimer's disease type 2
64267	Eu02y00	[X]Dementia in other specified diseases class if elsewhere

ICD-10 Codes for dementia used in HES and ONS

ICD Code	Description
F00	Dementia in Alzheimer disease
F00.0	Dementia in Alzheimer disease with early onset
F00.1	Dementia in Alzheimer disease with late onset
F00.2	Dementia in Alzheimer disease, atypical or mixed type
F00.9	Dementia in Alzheimer disease, unspecified
F01	Vascular dementia
F01.0	Vascular dementia of acute onset
F01.1	Multi-infarct dementia
F01.2	Subcortical vascular dementia
F01.3	Mixed cortical and subcortical vascular dementia
F01.8	Other vascular dementia
F01.9	Vascular dementia, unspecified
F02	Dementia in other diseases classified elsewhere
F02.0	Dementia in Pick disease
F02.1	Dementia in Creutzfeldt-Jakob disease
F02.2	Dementia in Huntington disease
F02.3	Dementia in Parkinson disease
F02.4	Dementia in human immunodeficiency virus [HIV] disease
F02.8	Dementia in other specified diseases classified elsewhere
F03	Unspecified dementia
F05.1	Delirium superimposed on dementia
G30	Alzheimer disease
G30.0	Alzheimer disease with early onset
G30.1	Alzheimer disease with late onset
G30.8	Other Alzheimer disease
G30.9	Alzheimer disease, unspecified
G31.0	Circumscribed brain atrophy
G31.8	Other specified degenerative diseases of nervous system

Read codes for coronary heart disease used in CPRD

Medcode	Read code	Description
240	G3...00	Ischemic heart disease
241	G30..00	Acute myocardial infarction
732	7928z00	Transluminal balloon angioplasty of coronary artery NOS
737	792..11	Coronary artery bypass graft operations
1204	G30..14	Heart attack
1344	G340.12	Coronary artery disease
1414	G33z300	Angina on effort
1430	G33..00	Angina pectoris
1431	G311.13	Unstable angina
1655	G340.11	Triple vessel disease of the heart
1676	G3z..00	Ischemic heart disease NOS
1677	G30..15	MI - acute myocardial infarction
1678	G308.00	Inferior myocardial infarction NOS
1792	G3...13	IHD - Ischemic heart disease
2155	G341000	Ventricular cardiac aneurysm
2491	G30..12	Coronary thrombosis
2901	7928	Transluminal balloon angioplasty of coronary artery
3159	792Dy00	Other specified other bypass of coronary artery
3704	G307.00	Acute subendocardial infarction
3999	G340000	Single coronary vessel disease
4017	G32..00	Old myocardial infarction
4656	G311.11	Crescendo angina
5030	ZV45K00	[V]Presence of coronary artery bypass graft
5254	G340100	Double coronary vessel disease
5387	G301.00	Other specified anterior myocardial infarction
5413	G340.00	Coronary atherosclerosis
5674	ZV45K11	[V]Presence of coronary artery bypass graft - CABG
5703	7928.11	Percutaneous balloon coronary angioplasty
5744	7927500	Open angioplasty of coronary artery
5904	792..00	Coronary artery operations
6182	7929y00	Other therapeutic transluminal op on coronary artery OS
6331	G341.00	Aneurysm of heart
6336	14A5.00	H/O: angina pectoris
6980	ZV45L00	[V]Status following coronary angioplasty NOS
7134	7921.11	Other autograft bypass of coronary artery
7137	7920y00	Saphenous vein graft replacement of coronary artery OS
7320	G343.00	Ischemic cardiomyopathy
7347	G311100	Unstable angina
7442	7920200	Saphenous vein graft replacement of three coronary arteries
7609	7921z00	Other autograft replacement of coronary artery NOS
7634	7920100	Saphenous vein graft replacement of two coronary arteries
7696	G33z200	Syncope anginosa
8312	7920.11	Saphenous vein graft bypass of coronary artery
8568	G37..00	Cardiac syndrome X
8679	7920000	Saphenous vein graft replacement of one coronary artery
8935	G302.00	Acute inferolateral infarction
8942	7929400	Insertion of coronary artery stent
9276	G31y000	Acute coronary insufficiency
9413	G31y.00	Other acute and subacute ischemic heart disease
9414	7921	Other autograft replacement of coronary artery

Medcode	Read code	Description
9507	G307000	Acute non-Q wave infarction
9555	G33z500	Post infarct angina
10209	7921200	Autograft replacement of three coronary arteries NEC
10562	G307100	Acute non-ST segment elevation myocardial infarction
10603	792z.00	Coronary artery operations NOS
11048	G331.11	Variant angina pectoris
11610	7920300	Saphenous vein graft replacement of four+ coronary arteries
11983	G311500	Acute coronary syndrome
12139	G300.00	Acute anterolateral infarction
12229	G30X000	Acute ST segment elevation myocardial infarction
12734	SP07600	Coronary artery bypass graft occlusion
12804	G33z700	Stable angina
12986	G331.00	Prinzmetal's angina
13566	G30..11	Attack - heart
13571	G30..16	Thrombosis - coronary
14658	G30z.00	Acute myocardial infarction NOS
14897	G301z00	Anterior myocardial infarction NOS
14898	G305.00	Lateral myocardial infarction NOS
15661	G310.11	Dressler's syndrome
15754	G34z.00	Other chronic ischemic heart disease NOS
16408	G32..11	Healed myocardial infarction
17133	G30A.00	Mural thrombosis
17307	G311200	Angina at rest
17464	G32..12	Personal history of myocardial infarction
17689	G30..17	Silent myocardial infarction
17872	G301100	Acute anteroseptal infarction
18118	G311400	Worsening angina
18125	G330000	Nocturnal angina
18249	7920	Saphenous vein graft replacement of coronary artery
18643	ZV45800	[V]Presence of coronary angioplasty implant and graft
18670	7928000	Percut transluminal balloon angioplasty one coronary artery
18842	G35..00	Subsequent myocardial infarction
18889	G34z000	Asymptomatic coronary heart disease
18913	ZV45700	[V]Presence of aortocoronary bypass graft
19046	7929300	Rotary blade coronary angioplasty
19193	7923z00	Prosthetic replacement of coronary artery NOS
19402	7923	Prosthetic replacement of coronary artery
19413	7921100	Autograft replacement of two coronary arteries NEC
19655	G311.14	Angina at rest
20095	G330.00	Angina decubitus
20903	7A6G100	Peroperative angioplasty
21844	G31y300	Transient myocardial ischaemia
22020	792B000	Endarterectomy of coronary artery NEC
22383	G3y..00	Other specified ischemic heart disease
22647	7925311	LIMA single anastomosis
22828	7929000	Percutaneous transluminal laser coronary angioplasty
23078	G34y100	Chronic myocardial ischaemia
23579	G310.00	Postmyocardial infarction syndrome
23708	G361.00	Atrial septal defect/curr comp folow acut myocardal infarct
23892	G304.00	Posterior myocardial infarction NOS
24126	G360.00	Haemopericardium/current comp folow acut myocard infarct

Medcode	Read code	Description
24540	G34y000	Chronic coronary insufficiency
24888	7929	Other therapeutic transluminal operations on coronary artery
25842	G33z.00	Angina pectoris NOS
26863	G33z600	New onset angina
27484	G341.11	Cardiac aneurysm
27951	G31..00	Other acute and subacute ischemic heart disease
27977	G31yz00	Other acute and subacute ischemic heart disease NOS
28138	G34..00	Other chronic ischemic heart disease
28554	G33zz00	Angina pectoris NOS
28736	G30y000	Acute atrial infarction
28837	7925.11	Creation of bypass from mammary artery to coronary artery
29421	G344.00	Silent myocardial ischaemia
29553	G366.00	Thrombosis atrium,auric append&vent/curr comp foll acute MI
29643	G303.00	Acute inferoposterior infarction
29758	G30X.00	Acute transmural myocardial infarction of unspecif site
29902	G330z00	Angina decubitus NOS
30330	G309.00	Acute Q-wave infarct
30421	G30..13	Cardiac rupture following myocardial infarction (MI)
31519	7925100	Double implant of mammary arteries into coronary arteries
31540	7924200	Revision of bypass for three coronary arteries
31556	7922	Allograft replacement of coronary artery
31571	792y.00	Other specified operations on coronary artery
31679	7929z00	Other therapeutic transluminal op on coronary artery NOS
32272	G38..00	Postoperative myocardial infarction
32450	G33z400	Ischemic chest pain
32651	7922.11	Allograft bypass of coronary artery
32854	G30B.00	Acute posterolateral myocardial infarction
33461	7924	Revision of bypass for coronary artery
33471	792Dz00	Other bypass of coronary artery NOS
33620	792B.00	Repair of coronary artery NEC
33650	7929100	Percut transluminal coronary thrombolysis with streptokinase
33718	7925000	Double anastomosis of mammary arteries to coronary arteries
33735	7928100	Percut translum balloon angioplasty mult coronary arteries
34328	G311300	Refractory angina
34633	G34y.00	Other specified chronic ischemic heart disease
34803	G30y.00	Other acute myocardial infarction
34963	792D.00	Other bypass of coronary artery
34965	792A.00	Diagnostic transluminal operations on coronary artery
35119	G501.00	Post infarction pericarditis
35674	14A3.00	H/O: myocardial infarct <60
35713	G34yz00	Other specified chronic ischemic heart disease NOS
36011	7923.11	Prosthetic bypass of coronary artery
36423	G36..00	Certain current complication follow acute myocardial infarct
36523	G311.00	Preinfarction syndrome
36609	G342.00	Atherosclerotic cardiovascular disease
36854	G332.00	Coronary artery spasm
37657	G362.00	Ventric septal defect/curr comp fol acut myocardal infarctn
37682	7925	Connection of mammary artery to coronary artery
37719	7925y00	Connection of mammary artery to coronary artery OS
38609	G351.00	Subsequent myocardial infarction of inferior wall
38813	7A54500	Rotary blade angioplasty

Medcode	Read code	Description
39449	G312.00	Coronary thrombosis not resulting in myocardial infarction
39546	Gyu3000	[X]Other forms of angina pectoris
39655	G311.12	Impending infarction
39693	G31y200	Subendocardial ischaemia
40399	14A4.00	H/O: myocardial infarct >60
40429	G301000	Acute anteroapical infarction
40996	7929111	Percut translum coronary thrombolytic therapy- streptokinase
41221	G30y200	Acute septal infarction
41547	7928y00	Transluminal balloon angioplasty of coronary artery OS
41677	G341z00	Aneurysm of heart NOS
41757	7927z00	Other open operation on coronary artery NOS
41835	G384.00	Postoperative subendocardial myocardial infarction
42304	7929500	Insertion of drug-eluting coronary artery stent
42462	7928200	Percut translum balloon angioplasty bypass graft coronary a
42708	7921300	Autograft replacement of four of more coronary arteries NEC
43939	793G.00	Perc translumin balloon angioplasty stenting coronary artery
44561	7921000	Autograft replacement of one coronary artery NEC
44585	792Bz00	Repair of coronary artery NOS
44723	7925200	Single anast mammary art to left ant descend coronary art
45370	7922300	Allograft replacement of four or more coronary arteries
45809	G350.00	Subsequent myocardial infarction of anterior wall
45886	7922200	Allograft replacement of three coronary arteries
46017	G30yz00	Other acute myocardial infarction NOS
46112	G380.00	Postoperative transmural myocardial infarction anterior wall
46166	G35X.00	Subsequent myocardial infarction of unspecified site
46276	G381.00	Postoperative transmural myocardial infarction inferior wall
47788	7927	Other open operations on coronary artery
48206	7927300	Transposition of coronary artery NEC
48767	7922z00	Allograft replacement of coronary artery NOS
48822	7925011	LIMA sequential anastomosis
50372	14AH.00	H/O: Myocardial infarction in last year
51507	7925300	Single anastomosis of mammary artery to coronary artery NEC
51515	7920z00	Saphenous vein graft replacement coronary artery NOS
51702	7927400	Exploration of coronary artery
52938	7924000	Revision of bypass for one coronary artery
54251	G311z00	Preinfarction syndrome NOS
54535	G33z100	Stenocardia
55092	792C000	Replacement of coronary arteries using multiple methods
55137	G311011	MI - myocardial infarction aborted
55598	792C.00	Other replacement of coronary artery
56905	792Ay00	Diagnostic transluminal operation on coronary artery OS
56990	7925z00	Connection of mammary artery to coronary artery NOS
57062	14AJ.00	H/O: Angina in last year
57241	7922100	Allograft replacement of two coronary arteries
57634	7924z00	Revision of bypass for coronary artery NOS
59189	G363.00	Ruptur cardiac wall w'out haemopericard/cur comp fol ac MI
59193	G341200	Aneurysm of coronary vessels
59423	7922y00	Other specified allograft replacement of coronary artery
59940	G364.00	Ruptur chordae tendinae/curr comp fol acute myocard infarct
60067	793G000	Perc translum ball angio insert 1-2 drug elut stents cor art
60753	7926300	Single implantation thoracic artery into coronary artery NEC

Medcode	Read code	Description
61208	793Gz00	Perc translum balloon angioplasty stenting coronary art NOS
61248	792Az00	Diagnostic transluminal operation on coronary artery NOS
61310	7921y00	Other autograft replacement of coronary artery OS
62608	7926000	Double anastom thoracic arteries to coronary arteries NEC
62626	G30y100	Acute papillary muscle infarction
63153	7924500	Revision of implantation of thoracic artery into heart
63467	G306.00	True posterior myocardial infarction
64923	7A6H300	Prosthetic graft patch angioplasty
66236	7923200	Prosthetic replacement of three coronary arteries
66388	G33z000	Status anginosus
66583	7929200	Percut translum inject therap subst to coronary artery NEC
66664	7923100	Prosthetic replacement of two coronary arteries
66921	7A6H400	Percutaneous transluminal angioplasty of vascular graft
67087	G341100	Other cardiac wall aneurysm
67554	7924100	Revision of bypass for two coronary arteries
67591	7926200	Single anastomosis of thoracic artery to coronary artery NEC
67761	7923300	Prosthetic replacement of four or more coronary arteries
68123	7925312	RIMA single anastomosis
68139	7925400	Single implantation of mammary artery into coronary artery
68357	G31y100	Microinfarction of heart
68748	G38z.00	Postoperative myocardial infarction, unspecified
69247	792By00	Other specified repair of coronary artery
69474	G365.00	Rupture papillary muscle/curr comp fol acute myocard infarct
70111	7922000	Allograft replacement of one coronary artery
70755	792Cz00	Replacement of coronary artery NOS
72562	G353.00	Subsequent myocardial infarction of other sites
72780	7926z00	Connection of other thoracic artery to coronary artery NOS
85947	793G200	Perc translum balloon angioplasty insert 1-2 stents cor art
86071	7928300	Percut translum cutting balloon angioplasty coronary artery
87849	793G100	Perc tran ball angio ins 3 or more drug elut stents cor art
91774	G341300	Acquired atrioventricular fistula of heart
92233	7925012	RIMA sequential anastomosis
92419	7923000	Prosthetic replacement of one coronary artery
92927	793G300	Percutaneous cor balloon angiop 3 more stents cor art NEC
93618	7929600	Percutaneous transluminal atherectomy of coronary artery
93706	793H000	Percutaneous transluminal balloon dilation cardiac conduit
93828	792Cy00	Other specified replacement of coronary artery
95382	7927y00	Other specified other open operation on coronary artery
96804	7926	Connection of other thoracic artery to coronary artery
96838	Gyu3400	[X]Acute transmural myocardial infarction of unspecif site
97953	7924y00	Other specified revision of bypass for coronary artery
99991	Gyu3600	[X]Subsequent myocardial infarction of unspecified site
101569	7924300	Revision of bypass for four or more coronary arteries
105250	G341111	Mural cardiac aneurysm
105479	G39..00	Coronary microvascular disease
106812	G383.00	Postoperative transmural myocardial infarction unspec site
109035	Gyu3500	[X]Subsequent myocardial infarction of other sites

ICD-10 Codes for coronary heart disease used in HES and ONS

ICD Code	Description
I20	Angina pectoris
I20.0	Unstable angina
I20.1	Angina pectoris with documented spasm
I20.8	Other forms of angina pectoris
I20.9	Angina pectoris, unspecified
I21	Acute myocardial infarction
I21.0	Acute transmural myocardial infarction of anterior wall
I21.1	Acute transmural myocardial infarction of inferior wall
I21.2	Acute transmural myocardial infarction of other sites
I21.3	Acute transmural myocardial infarction of unspecified site
I21.4	Acute subendocardial myocardial infarction
I21.9	Acute myocardial infarction, unspecified
I22	Subsequent myocardial infarction
I22.0	Subsequent myocardial infarction of anterior wall
I22.1	Subsequent myocardial infarction of inferior wall
I22.8	Subsequent myocardial infarction of other sites
I22.9	Subsequent myocardial infarction of unspecified site
I23	Certain current complications following acute myocardial infarction
I23.0	Haemopericardium as current complication following acute myocardial infarction
I23.1	Atrial septal defect as current complication following acute myocardial infarction
I23.2	Ventricular septal defect as current complication following acute myocardial infarction
I23.3	Rupture of cardiac wall without haemopericardium as current complication following acute myocardial infarction
I23.4	Rupture of chordae tendineae as current complication following acute myocardial infarction
I23.5	Rupture of papillary muscle as current complication following acute myocardial infarction
I23.6	Thrombosis of atrium, auricular appendage, and ventricle as current complications following acute myocardial infarction
I23.8	Other current complications following acute myocardial infarction
I24	Other acute ischemic heart diseases
I24.0	Coronary thrombosis not resulting in myocardial infarction
I24.1	Dressler syndrome
I24.8	Other forms of acute ischemic heart disease
I24.9	Acute ischemic heart disease, unspecified
I25	Chronic ischemic heart disease
I25.0	Atherosclerotic cardiovascular disease, so described
I25.1	Atherosclerotic heart disease
I25.2	Old myocardial infarction
I25.3	Aneurysm of heart
I25.4	Coronary artery aneurysm and dissection
I25.5	Ischemic cardiomyopathy
I25.6	Silent myocardial ischaemia
I25.8	Other forms of chronic ischemic heart disease
I25.9	Chronic ischemic heart disease, unspecified

Read codes for fracture used in CPRD

Medcode	Read code	Description
137	S23x111	Fracture of radius NOS
169	S35..11	Metatarsal bone fracture
199	S23B.00	Fracture of lower end of radius
203	S234.11	Wrist fracture - closed
235	S32..00	Fracture of patella
280	S120.00	Closed fracture rib
325	S34..00	Fracture of ankle
343	S234100	Closed Colles' fracture
358	S3z..11	Fracture NOS
417	S020.00	Closed fracture nose
441	S26..00	Fracture of one or more phalanges of hand
455	S3z0000	Greenstick fracture
482	S26..12	Thumb fracture excluding base
483	S20..00	Fracture of clavicle
517	S22..00	Fracture of humerus
520	S31z.00	Fracture of femur, NOS
553	S242000	Fracture of scaphoid
721	S0...00	Fracture of skull
738	S13..00	Fracture or disruption of pelvis
806	S339.00	Fracture of fibula alone
835	S10B200	Fracture of coccyx
845	S35..00	Fracture of one or more tarsal and metatarsal bones
868	S4...13	Fracture dislocations and fracture subluxations
909	S23z.00	Fracture of radius and ulna, NOS
953	S3z1.00	Open fracture of bones, unspecified
971	S33x000	Closed fracture of tibia, unspecified part, NOS
993	S242200	Fracture of other metacarpal bone
1073	S23x211	Fracture of ulna NOS
1177	S21..00	Fracture of scapula
1179	S28..11	Ill-defined fracture of arm
1250	S224.11	Elbow fracture - closed
1548	S228.00	Fracture of lower end of humerus
1591	S130.00	Closed fracture acetabulum
1700	S352.11	March fracture
1742	S234200	Closed fracture of the distal radius, unspecified
1857	S354.00	Fracture of calcaneus
1873	S36..11	Toe fracture
1994	S30..11	Hip fracture
2101	S226.00	Fracture of upper end of humerus
2176	S362.00	Fracture of great toe
2225	S30..00	Fracture of neck of femur
2250	S344.12	Pott's fracture - ankle
2251	S024100	Closed fracture zygoma
2303	S237.00	Fracture of upper end of radius
2328	S10B500	Fracture of pubis
2442	S355.00	Fracture of talus
2461	S01..00	Fracture of base of skull
2470	S3z..00	Fracture of unspecified bones
2603	S3...11	Leg fracture
2630	S33..00	Fracture of tibia and fibula
2642	S022.00	Fracture of mandible, closed
2643	S25..11	Hand fracture - metacarpal bone
2660	S28z.00	Ill-defined fractures of upper limb NOS
2662	S230300	Closed Monteggia's fracture
2672	S36..00	Fracture of one or more phalanges of foot
2710	S35..12	Tarsal bone fracture
2862	S234700	Closed Smith's fracture
2887	7K1L100	Manipulation of fracture of bone NEC

Medcode	Read code	Description
2888	S25..00	Fracture of metacarpal bone
3025	S3z2.00	Stress fracture
3095	7J03100	Reduction of fracture of nasal bones NEC
3288	S10A.00	Fracture of neck
3408	S021.00	Open fracture nose
3573	S10x.00	Closed fracture of spine, unspecified,
3675	S10B100	Fracture of sacrum
3748	S233.00	Open fracture of radius and ulna, shaft
3809	S00..11	Frontal bone fracture
3888	S104.00	Closed fracture lumbar vertebra
3937	S352700	Closed fracture metatarsal
3983	S122.00	Closed fracture sternum
4029	S210300	Closed fracture scapula, glenoid
4211	S20..11	Collar bone fracture
4225	S024000	Closed fracture maxilla
4304	S33x100	Closed fracture of fibula, unspecified part, NOS
4306	S360.00	Closed fracture of one or more phalanges of foot
4310	S352300	Closed fracture cuboid
4350	7J13400	Reduction of Le Fort 1 fracture of maxilla
4359	S23x300	Closed fracture of the radius and ulna
4409	S10..12	Fracture of vertebra without spinal cord lesion
4465	7J03200	Reduction of fracture of zygomatic bones
4528	7K1G.00	Other primary open reduction of fracture of bone
4572	S33x200	Closed fracture of tibia and fibula, unspecified part
4582	S26z.00	Fracture of one or more phalanges of hand NOS
4629	7K1L.00	Other closed reduction of fracture of bone
4641	7K1T100	Debridement of open fracture
4725	S242300	Multiple fractures of metacarpal bones
4737	S34x.00	Closed fracture ankle, unspecified
4978	S02x100	Fracture of orbit NOS, closed
5009	S348.00	Fracture of medial malleolus
5260	S26..11	Finger fracture
5280	S028000	Fracture of nasal bones
5301	S302.00	Closed fracture of proximal femur, pertrochanteric
5302	S132.00	Closed fracture pubis
5332	S312300	Closed fracture distal femur, supracondylar
5344	S210400	Closed fracture scapula, blade
5345	S4A..00	Fracture-dislocation or subluxation shoulder
5354	S240400	Closed fracture pisiform
5381	S15..00	Fracture of thoracic vertebra
5445	S100100	Closed fracture atlas
5526	N331.00	Pathological fracture
5567	S01..19	Temporal bone fracture
5886	7K1L700	Closed reduction of fracture of tibia and or fibula
5929	S2...11	Arm fracture
5951	7K1LM00	Closed reduction of fracture of wrist
6062	S356.00	Fracture of metatarsal bone
6069	7K1LH00	Closed reduction of fracture of finger
6074	S235100	Open Colles' fracture
6106	7K1L800	Closed reduction of fracture of ankle
6168	S240100	Closed fracture of the scaphoid
6195	S2...00	Fracture of upper limb
6213	S23C.00	Fracture of lower end of both ulna and radius
6248	7K1J.00	Closed (or no) reduction of fracture and internal fixation
6286	S340.00	Closed fracture ankle, medial malleolus
6299	S263.00	Fracture of other finger
6320	S312100	Closed fracture of femoral condyle, unspecified
6379	7K1LF00	Closed reduction of fracture of humerus
6380	S235B00	Open fracture radial styloid

Medcode	Read code	Description
6392	S250.00	Closed fracture of metacarpal bone(s)
6660	7K1L400	Closed reduction of fracture of hip
6667	S132100	Closed fracture pelvis, multiple pubic rami - stable
6731	S349.00	Fracture of lateral malleolus
6734	7K14.00	Open surgical fracture of bone
6825	S23..00	Fracture of radius and ulna
6839	S339000	Closed fracture of distal fibula
6868	S310.00	Closed fracture of femur, shaft or unspecified part
6881	S250z00	Closed fracture of metacarpal bone(s) NOS
6893	S224100	Closed fracture distal humerus, supracondylar
6915	S234B00	Closed fracture radial styloid
6917	S336.00	Fracture of upper end of tibia
6942	7K1LL00	Closed reduction of fracture of radius and or ulna
6994	7J12.00	Reduction of fracture of mandible
7004	S132000	Closed fracture pelvis, single pubic ramus
7009	S230600	Closed fracture radius, head
7034	7K1LE00	Closed reduction of fracture of elbow
7135	S342000	Closed fracture ankle, lateral malleolus, low
7159	S363.00	Fracture of other toe
7317	S344.00	Closed fracture ankle, bimalleolar
7339	7K1LA00	Closed reduction of fracture of toe
7340	S342.00	Closed fracture ankle, lateral malleolus
7393	7K15.00	Other surgical fracture of bone
7428	7K1LG00	Closed reduction of fracture of shoulder
7500	S262.00	Fracture of thumb
7531	S252.00	Closed fracture sesamoid bone of hand
7564	S242100	Fracture of first metacarpal bone
7636	S231600	Open fracture radial head
7660	S230700	Closed fracture radius, neck
7672	7K1D.00	Primary open reduction fracture bone & intramedull fixation
7723	S337.00	Fracture of shaft of tibia
7754	S225.11	Elbow fracture - open
7831	S120000	Closed fracture of rib, unspecified
7930	7K1F500	Primary open reduction fracture patella fixat tension band
7988	S239.00	Fracture of shaft of radius
8040	S31..00	Other fracture of femur
8056	S242.00	Fracture at wrist and hand level
8199	S264.00	Multiple fractures of fingers
8243	S305.00	Subtrochanteric fracture
8255	S10..00	Fracture of spine without mention of spinal cord injury
8263	S350.11	Heel bone fracture
8266	S104100	Closed fracture lumbar vertebra, wedge
8276	S350.00	Closed fracture of calcaneus
8302	S260.00	Closed fracture of one or more phalanges of hand
8348	S4A0.00	Closed fracture-dislocation shoulder
8382	S238.00	Fracture of shaft of ulna
8410	S231B00	Open fracture olecranon, intra-articular
8465	S334100	Closed fracture distal tibia, intra-articular
8573	S00..12	Parietal bone fracture
8589	S315.00	Fracture of lower end of femur
8613	S10B600	Multiple fractures of lumbar spine and pelvis
8646	S314.00	Fracture of shaft of femur
8648	S302400	Closed fracture of femur, intertrochanteric
8661	S224600	Closed fracture distal humerus, lateral epicondyle
8704	S23y300	Open fracture of the radius and ulna
8800	7K1L600	Closed reduction of fracture of knee
8885	7K1LJ00	Closed reduction of fracture of thumb
8891	S3...00	Fracture of lower limb
8915	S293.00	Multiple fractures of forearm

Medcode	Read code	Description
8968	S120A00	Cough fracture
9072	S10B400	Fracture of acetabulum
9103	S02..00	Fracture of face bones
9165	S234300	Closed fracture of ulna, styloid process
9174	S3x4.00	Multiple fractures of foot
9212	S34z.00	Fracture of ankle, NOS
9261	S4C1000	Open fracture-dislocation, distal radio-ulnar joint
9348	S3x3.00	Multiple fractures of lower leg
9420	S221.00	Open fracture of the proximal humerus
9468	S4B0000	Closed fracture-dislocation elbow joint
9538	S230100	Closed fracture olecranon, extra-articular
9688	S127.00	Fracture of rib
9736	S01..15	Occiput bone fracture
9771	S020.11	Closed fracture nasal bone
9917	S347.00	Open fracture ankle, trimalleolar
10007	S338.00	Fracture of lower end of tibia
10009	S346.00	Closed fracture ankle, trimalleolar
10022	S235.11	Wrist fracture - open
10033	S234F00	Closed Barton's fracture
10095	S311100	Open fracture shaft of femur
10102	7K1Ky00	Closed reduction of bone fracture and external fixation OS
10149	S23A.00	Fracture of shafts of both ulna and radius
10167	S24..11	Hand fracture - carpal bone
10228	S4B..00	Fracture-dislocation or subluxation elbow
10246	S231300	Open Monteggia's fracture
10250	S4D..00	Fracture-dislocation/subluxation finger/thumb
10252	S1...00	Fracture of neck and trunk
10357	S240200	Closed fracture lunate
10382	S22z.00	Fracture of humerus NOS
10462	S4D0500	Closed fracture-dislocation, interphalangeal joint thumb
10570	S30y.11	Hip fracture NOS
10622	S221.11	Shoulder fracture - open
10640	S23..11	Forearm fracture
10696	S127000	Multiple fractures of ribs
10735	S21..11	Shoulder blade fracture
10736	7206100	Open reduction of fracture of orbit
10737	82...11	Closed reduction of fracture
10990	S10B000	Fracture of lumbar vertebra
11004	S120900	Closed fracture multiple ribs
11044	S220300	Closed fracture proximal humerus, greater tuberosity
11066	S234900	Closed volar Barton's fracture
11161	S028300	Fracture of mandible
11222	S220.00	Closed fracture of the proximal humerus
11262	S235300	Open fracture of ulna, styloid process
11275	S3X..00	Fracture of lower leg, part unspecified
11277	S150.00	Multiple fractures of thoracic spine
11296	S100.00	Closed fracture of cervical spine
11313	S220100	Closed fracture proximal humerus, neck
11333	7K1K800	Primary external fixation of fracture
11342	7J12200	Closed reduction of fracture of mandible NEC
11378	S12z.11	Rib fracture NOS
11453	7K1LB00	Closed reduction of fracture of hallux
11503	N331M00	Fragility fracture due to unspecified osteoporosis
11635	S352200	Closed fracture navicular
11639	S134z00	Other or multiple closed fracture of pelvis NOS
11770	S102y00	Other specified closed fracture thoracic vertebra
11872	7K1LV00	Primary closed reduction of fracture alone
11969	S128.00	Fracture of sternum
12001	Q203.12	Other birth fracture

Medcode	Read code	Description
12063	S230B00	Closed fracture olecranon, intra-articular
12179	S022100	Closed fracture of mandible, condylar process
12369	S339100	Open fracture of distal fibula
12406	S10B.00	Fracture of lumbar spine and pelvis
12462	S025100	Open fracture zygoma
12516	S2B..00	Fracture of bone of hand
12546	S250400	Closed fracture finger metacarpal neck
12673	N331900	Osteoporosis + pathological fracture thoracic vertebrae
12791	S310011	Thigh fracture NOS
12823	7K1J300	Closed reduction fracture small bone & fixation using screw
14746	S3xz.00	Other, multiple and ill-defined fractures of lower limb NOS
14826	S344.11	Dupuytren's fracture, fibula
14834	S108.00	Closed fracture pelvis, coccyx
14878	S024.00	Fracture of malar or maxillary bones, closed
15079	S352100	Closed fracture of talus
15085	7K1Lz00	Other closed reduction of fracture of bone NOS
15166	S350.12	Os calcis fracture
15184	S00..00	Fracture of vault of skull
15376	S224.00	Closed fracture of the distal humerus
15491	S4G..00	Fracture-dislocation or subluxation ankle
15613	S100000	Closed fracture of unspecified cervical vertebra
15622	7J42500	Spinal traction for fracture of spine NEC
15666	S240.00	Closed fracture of carpal bone
15764	S23x.00	Closed fracture of radius and ulna, unspecified part
15800	7J03.00	Reduction of fracture of facial bone
15877	S106.00	Closed fracture sacrum
15927	S352.00	Closed fracture of other tarsal and metatarsal bones
16141	7K1Gz00	Other primary open reduction of fracture of bone NOS
16277	S100200	Closed fracture axis
16389	S210100	Closed fracture scapula, acromion
16494	S120100	Closed fracture of one rib
16769	TC7..00	Fracture, cause unspecified
16866	S225700	Open fracture distal humerus, medial epicondyle
16890	S022.12	Fracture of lower jaw, closed
16895	N1y1.00	Fatigue fracture of vertebra
16944	S292.00	Multiple fractures of clavicle, scapula and humerus
16985	S240A00	Closed fracture scaphoid, proximal pole
17107	S29..12	Multiple rib fractures
17138	7J17700	Traction for fracture of jaw
17249	S4H..00	Fracture-dislocation or subluxation foot
17286	S241100	Open fracture of the scaphoid
17377	N331800	Osteoporosis + pathological fracture lumbar vertebrae
17443	7J12.11	Reduction of fracture of jaw NEC
17455	S02z.11	Jaw fracture NOS
17606	S240700	Closed fracture capitate
17822	S230200	Closed fracture of ulna, coronoid
17921	S4C2.00	Closed fracture-subluxation of the wrist
17922	S4C0000	Closed fracture-dislocation distal radio-ulnar joint
17952	S23x100	Closed fracture of radius (alone), unspecified
17956	S201200	Open fracture clavicle, shaft
18273	S30y.00	Closed fracture of neck of femur NOS
18299	S234.00	Closed fracture of radius and ulna, lower end
18301	S235F00	Open Barton's fracture
18336	S261.00	Open fracture of one or more phalanges of hand
18338	S260F00	Closed fracture finger proximal phalanx, shaft
18388	S343.00	Open fracture ankle, lateral malleolus
18389	S234000	Closed fracture of forearm, lower end, unspecified
18394	S224200	Closed fracture distal humerus, lateral condyle
18584	S345.00	Open fracture ankle, bimalleolar

Medcode	Read code	Description
18614	S4C..00	Fracture-dislocation or subluxation of wrist
18676	B585000	Pathological fracture due to metastatic bone disease
18825	NyuB800	[X]Unspecified osteoporosis with pathological fracture
18840	S330300	Closed fracture proximal tibia, medial condyle (plateau)
18841	S4D1.00	Open fracture-dislocation digit
18962	7K1L500	Closed reduction of fracture of femur
19058	S234D00	Closed fracture distal radius, extra-articular, other type
19186	S222000	Closed fracture of humerus NOS
19189	S10A200	Multiple fractures of cervical spine
19206	SR1..00	Fractures involving multiple body regions
19375	S4D0400	Closed fracture-dislocation, proximal interphalangeal joint
19387	S302011	Closed fracture of femur, greater trochanter
19403	S240B00	Closed fracture scaphoid, waist, transverse
20195	S00z.00	Fracture of vault of skull NOS
20253	S352000	Closed fracture of tarsal bone, unspecified
20445	7206400	Open reduction of fracture of orbit and internal fixation
20515	S028100	Fracture of orbital floor
20598	7J43.00	Fixation of fracture of spine
20678	S333200	Open fracture of tibia and fibula, shaft
20744	7J42.00	Other reduction of fracture of spine
20893	S310012	Upper leg fracture NOS
21175	S250x00	Closed fractures of multiple sites of unspecified metacarpus
21773	S3x2.00	Multiple fractures of femur
21922	S312200	Closed fracture of femur, lower epiphysis
21942	7K1Gy11	Primary open reduction of bone fracture & external fixation
22144	7K6GN00	Closed reduction fracture disloc joint & internal fixation
22329	S312.11	Closed fracture of femur, distal end
22370	S330400	Closed fracture proximal tibia, lateral condyle (plateau)
22375	S24..00	Fracture of carpal bone
22761	S330012	Closed fracture of tibial tuberosity
22780	7J13.00	Reduction of fracture of maxilla
23780	S03z.11	Depressed skull fracture NOS
23803	S301800	Open fracture proximal femur,subcapital, Garden grade III
23987	S234211	Dupuytren's fracture, radius - closed
24135	S01..14	Middle fossa fracture
24276	S30w.00	Closed fracture of unspecified proximal femur
24516	S260D00	Closed fracture finger proximal phalanx
24534	S4A2100	Closed fracture-subluxation acromio-clavicular joint
24587	S4E..00	Fracture-dislocation or subluxation hip
24598	S250500	Closed fracture finger metacarpal head
24611	7K6F200	Primary open reduction of fracture dislocation of joint NEC
24615	S4J1200	Open fracture-dislocation sterno-clavicular joint, anterior
24620	S352B00	Closed fracture metatarsal base
24621	S23x200	Closed fracture of ulna (alone), unspecified
24671	S101500	Open fracture of fifth cervical vertebra
24672	S100700	Closed fracture of seventh cervical vertebra
24674	S310100	Closed fracture shaft of femur
24715	7K1K.00	Closed (or no) reduction of fracture and external fixation
24790	S026.00	Closed orbital blow-out fracture
25073	S360000	Closed fracture proximal phalanx, toe
25173	7J03000	Reduction of fracture of nasoethmoid complex of bones
25284	S1z..00	Fracture of neck and trunk NOS
25312	7J13300	Reduction of blowout fracture of orbital floor
25445	S4D0100	Closed fracture-dislocation, metacarpophalangeal joint
25485	S33z.00	Fracture of tibia and fibula, NOS
25519	S250300	Closed fracture finger metacarpal shaft
25631	S02z.00	Fracture of facial bone NOS
25811	S250800	Closed fracture of thumb metacarpal
26045	S240500	Closed fracture trapezium

Medcode	Read code	Description
26130	7K1LD00	Closed reduction of fracture of nasal bone
26324	S232.00	Closed fracture of radius and ulna, shaft
26408	S02xz00	Fracture of other facial bones, closed, NOS
27287	S02x000	Fracture of alveolus, closed
27361	7J12y00	Other specified reduction of fracture of mandible
27404	S102.00	Closed fracture thoracic vertebra
27492	S031.00	Closed fracture of skull NOS with intracranial injury
27567	S352C00	Closed fracture metatarsal shaft
27575	S100500	Closed fracture of fifth cervical vertebra
27590	S235.00	Open fracture of radius and ulna, lower end
27591	S234z00	Closed fracture of forearm, lower end, NOS
27597	N331600	Idiopathic osteoporosis with pathological fracture
27605	S294.00	Fractures involving multiple regions of both upper limbs
27620	S210.00	Closed fracture of scapula
27643	S260S00	Closed fracture finger distal phalanx, base
27654	S100600	Closed fracture of sixth cervical vertebra
27657	S001.00	Closed fracture vault of skull with intracranial injury
27699	S260E00	Closed fracture finger proximal phalanx, base
27718	S4A0100	Closed fracture-dislocation acromio-clavicular joint
27719	S334.00	Closed fracture distal tibia
27721	S335000	Open fracture distal tibia, extra-articular
27783	S4C0300	Closed fracture-dislocation, carpometacarpal joint
27784	S233z00	Open fracture of radius and ulna, shaft, NOS
27818	S12z.12	Sternum fracture NOS
27854	S134600	Closed fracture pelvis, iliac wing
27881	S250600	Closed fracture finger metacarpal
27886	S222100	Closed fracture of humerus, shaft
27922	S10B300	Fracture of ilium
27992	S335.00	Open fracture distal tibia
28066	S20z.00	Fracture of clavicle NOS
28068	S333.00	Open fracture of tibia/fibula, shaft
28070	S4G3.00	Open fracture-subluxation, ankle joint
28118	S333000	Open fracture shaft of tibia
28133	S10A000	Fracture of first cervical vertebra
28179	S200z00	Closed fracture of clavicle NOS
28197	S261R00	Open fracture finger distal phalanx
28198	S333z00	Open fracture of tibia and fibula, shaft, NOS
28233	S33y.00	Open fracture of tibia and fibula, unspecified part, NOS
28234	S134400	Closed fracture pelvis, anterior superior iliac spine
28244	S120z00	Closed fracture of rib(s) NOS
28249	S260800	Closed fracture thumb distal phalanx
28251	S360200	Closed fracture distal phalanx, toe
28273	S321.00	Open fracture of the patella
28293	S234E00	Closed fracture distal radius, intra-articular, other type
28307	S200200	Closed fracture clavicle, shaft
28352	S33y100	Open fracture of fibula, unspecified part, NOS
28371	S352F00	Closed fracture metatarsal, multiple
28375	S13y.00	Closed fracture of pelvis NOS
28393	S224800	Closed fracture distal humerus, capitellum
28413	S240300	Closed fracture triquetral
28425	S240E00	Closed fracture scaphoid, tuberosity
28426	S332100	Closed fracture shaft of fibula
28524	S102100	Closed fracture thoracic vertebra, wedge
28538	S127100	Cough fracture of ribs
28550	S330000	Closed fracture of the proximal tibia
28604	S362000	Closed fracture of great toe
28621	7J03z00	Reduction of fracture of facial bone NOS
28702	S132z00	Closed fracture pubis NOS
28708	S234600	Closed fracture radius and ulna, distal

Medcode	Read code	Description
28724	S224700	Closed fracture distal humerus, medial epicondyle
28731	S4F1.00	Open fracture-dislocation, knee joint
28739	S220400	Closed fracture proximal humerus, head
28741	S23y200	Open fracture of ulna (alone), unspecified
28800	S4H0400	Closed fracture-dislocation, IPJ, single toe
28875	S352J00	Closed fracture of base of fifth metatarsal
28913	S022400	Closed fracture of mandible, ramus, unspecified
28926	7J12100	Open reduction of fracture of mandible NEC
28954	S312.00	Closed fracture distal femur
28965	S304.00	Pertrochanteric fracture
28966	7J43000	Primary open reduc spinal fracture+internal fix+plate
29084	S33y200	Open fracture of tibia and fibula, unspecified part
29089	S104400	Closed fracture lumbar vertebra, transverse process
29091	S022000	Closed fracture mandible (site unspecified)
29103	7K1KE00	Primary closed reduction of fracture and external fixation
29109	S33x.00	Closed fracture of tibia and fibula, unspecified part, NOS
29111	S251.00	Open fracture of metacarpal bone(s)
29117	S260R00	Closed fracture finger distal phalanx
29119	S02x.00	Closed fracture other facial bone
29121	S332.00	Closed fracture of tibia/fibula, shaft
29137	S220700	Closed fracture proximal humerus, four part
29145	S302200	Closed fracture proximal femur, subtrochanteric
29152	S231100	Open fracture olecranon, extra-articular
29162	S3zz.00	Fracture of bones NOS
29164	S33y000	Open fracture of tibia, unspecified part, NOS
29332	N331y00	Other specified pathological fracture
29582	7K1D511	K wiring of fracture
29748	S352D00	Closed fracture metatarsal neck
29804	S360100	Closed fracture middle phalanx, toe
29899	S200300	Closed fracture clavicle, lateral end
29911	S4F..00	Fracture-dislocation or subluxation knee
29981	S4F4.00	Closed fracture-dislocation, patello-femoral joint
30028	S028200	Fracture of malar and maxillary bones
30058	S10..11	Fracture of transverse process spine - no spinal cord lesion
30076	S2z..00	Fracture of upper limb NOS
30203	S028.00	Fracture of skull and facial bones
30213	7K1L300	Remanipulation of fracture of bone NEC
30288	7J13000	Reduction of fracture of alveolus of maxilla
30352	N331100	Pathological fracture of lumbar vertebra
30377	Z6G1900	Fracture - traction
30418	S235800	Open Galeazzi fracture
30429	7K1F.00	Primary open reduction of intraarticular fracture of bone
30611	S352111	Closed fracture of astragalus
30616	N331000	Pathological fracture of thoracic vertebra
30659	S227.00	Fracture of shaft of humerus
30707	S01..12	Ethmoid sinus fracture
30956	S11x.00	Closed fracture of spine with spinal cord lesion unspecified
31153	S02y100	Fracture of orbit NOS, open
31525	S250000	Closed fracture of metacarpal bone (s), site unspecified
31545	S112700	Cls spinal fracture with complete thorac cord lesion, T7-12
31708	S29..11	Multiple fractures of arm
31760	S225100	Open fracture distal humerus, supracondylar
31797	S04..11	Multiple face fractures
31847	S362100	Open fracture of great toe
31933	7J41.00	Decompression of fracture of spine
31999	S240800	Closed fracture hamate
32009	SR10.00	Fractures involving head with neck
32011	S025000	Open fracture maxilla
32012	7K1Le00	Primary arthroscopic reduction and fixation of fracture

Medcode	Read code	Description
32063	S11..00	Fracture of spine with spinal cord lesion
32178	7K14y00	Other specified open surgical fracture of bone
32298	S6z..00	Intracranial injury, excluding those with skull fracture NOS
32348	S224400	Closed fracture of distal humerus, condyle(s) unspecified
32638	S125200	Closed fracture of thyroid cartilage
32646	S225800	Open fracture distal humerus, capitellum
32866	S313.11	Open fracture of femur, distal end
33357	7K15z00	Other surgical fracture of bone NOS
33393	S320.00	Closed fracture of the patella
33404	S236.00	Fracture of upper end of ulna
33457	S331100	Open fracture proximal fibula
33459	S027.00	Open orbital blow-out fracture
33475	S321200	Open fracture patella, distal pole
33489	S220200	Closed fracture of proximal humerus, anatomical neck
33503	S100611	C6 vertebra closed fracture without spinal cord lesion
33515	S044.00	Multiple fractures involving skull and facial bones
33520	S332200	Closed fracture of tibia and fibula, shaft
33526	N331300	Osteoporosis of disuse with pathological fracture
33540	S224z00	Closed fracture of distal humerus, not otherwise specified
33582	S260300	Closed fracture thumb proximal phalanx
33593	S01z.00	Fracture of base of skull NOS
33598	S260L00	Closed fracture finger middle phalanx, base
33602	7K1G400	Primary open reduction of fracture and cast immobilisation
33616	S260K00	Closed fracture finger middle phalanx
33651	S260700	Closed fracture thumb proximal phalanx, head
33656	S330100	Closed fracture proximal fibula
33666	S4F2.00	Closed fracture-subluxation, knee joint
33678	S260400	Closed fracture thumb proximal phalanx, base
33679	S260G00	Closed fracture finger proximal phalanx, neck
33680	S223.00	Open fracture of humerus, shaft or unspecified part
33684	S261D00	Open fracture finger proximal phalanx
33687	S4B0.00	Closed fracture-dislocation elbow
33692	S0z..00	Fracture of skull NOS
33695	S260T00	Closed fracture finger distal phalanx, shaft
33704	S4A0000	Closed fracture-dislocation shoulder joint
33706	S331000	Open fracture of the proximal tibia
33720	S224000	Closed fracture of elbow, unspecified part
33749	S200.00	Closed fracture of clavicle
33757	S260M00	Closed fracture finger middle phalanx, shaft
33768	S330600	Closed fracture spine, tibia
33808	S232z00	Closed fracture of radius and ulna, shaft, NOS
33845	S260U00	Closed fracture finger distal phalanx, tuft
33866	S261M00	Open fracture finger middle phalanx, shaft
33870	S21z.00	Fracture of scapula NOS
33882	S2A..00	Fracture of upper limb, level unspecified
33883	S230900	Closed fracture of the proximal radius
33903	S37..00	Fracture of lower limb, level unspecified
33905	S250200	Closed fracture finger metacarpal base
33908	S27..00	Multiple fractures of hand bones
33929	S240z00	Closed fracture of carpal bone NOS
33933	S230000	Closed fracture of proximal forearm, unspecified part
33957	S300700	Closed fracture proximal femur, subcapital, Garden grade II
33961	S134.00	Other or multiple closed fracture of pelvis
33967	S100211	C2 vertebra closed fracture without spinal cord lesion
33974	S341.00	Open fracture ankle, medial malleolus
33985	S4D0300	Closed fracture-dislocation, distal interphalangeal joint
33990	S271.00	Open multiple fractures of hand bones
34021	S332000	Closed fracture shaft of tibia
34058	S260000	Closed fracture of phalanx or phalanges, unspecified

Medcode	Read code	Description
34078	S300900	Closed fracture proximal femur, subcapital, Garden grade IV
34080	S260900	Closed fracture thumb distal phalanx, base
34106	S311000	Open fracture of femur, unspecified part
34151	S334000	Closed fracture distal tibia, extra-articular
34166	S10z.00	Fracture of spine without mention of spinal cord lesion NOS
34172	S225.00	Open fracture of the distal humerus
34195	S134800	Closed fracture dislocation of sacro-iliac joint
34197	S120400	Closed fracture of four ribs
34212	S4J2100	Closed fracture-subluxation of pelvis
34277	7K1Dy00	Prim open reduction fracture bone & intramedullary fixatn OS
34302	S4G0.00	Closed fracture-dislocation, ankle joint
34307	S260P00	Closed fracture finger middle phalanx, head
34351	S300600	Closed fracture proximal femur, subcapital, Garden grade I
34355	7K1JK00	Primary closed reduction of fracture and wire fixation
34356	S260N00	Closed fracture finger middle phalanx, neck
34367	S23y100	Open fracture of radius (alone), unspecified
34370	S230800	Closed fracture proximal radius, comminuted
34371	S230400	Closed fracture of proximal ulna, comminuted
34396	7K1G100	Prmy open reduction of fracture and external fixation
34403	S10A100	Fracture of second cervical vertebra
34424	S36z.00	Fracture of one or more phalanges of foot NOS
34426	S230500	Closed fracture of the proximal ulna
34429	S4C0.00	Closed fracture dislocation of wrist
34632	7J02200	Elevation of depressed fracture of cranium
34634	7J42100	Open reduction of fracture of spine NEC
34685	S133000	Open fracture pelvis, single pubic ramus
34708	S134100	Closed fracture pelvis, ischium
34723	S350100	Closed fracture calcaneus, intra-articular
34730	S235700	Open Smith's fracture
34737	S231700	Open fracture radial neck
34738	S313500	Open fracture distal femur, lateral condyle
34850	7J43100	Fixation of fracture of spine using Harrington rod
34873	S100511	C5 vertebra closed fracture without spinal cord lesion
34907	S210200	Closed fracture scapula, coracoid
34910	S4J0100	Closed fracture-dislocation of pelvis
34954	S352400	Closed fracture medial cuneiform
34993	S4J0.00	Other closed fracture-dislocation
35011	S32z.00	Fracture of patella, NOS
35018	S135z00	Other/multiple open fracture of pelvis NOS
35031	S232300	Closed fracture radius and ulna, middle
35077	S352E00	Closed fracture metatarsal head
35096	S104300	Closed fracture lumbar vertebra, spinous process
35198	S4D0.00	Closed fracture-dislocation digit
35253	S33x.11	Lower leg fracture NOS
35260	S150000	Closed multiple fractures of thoracic spine
35312	S02yz00	Fracture of other facial bones,open, NOS
35386	S211.00	Open fracture of scapula
35456	S6...00	Intracranial injury excluding those with skull fracture
35530	7K1LK00	Closed reduction of fracture of metacarpus
35620	S342100	Closed fracture ankle, lateral malleolus, high
35789	S4A2.00	Closed fracture-subluxation shoulder
35837	S240600	Closed fracture trapezoid
35849	S112.00	Closed fracture of thoracic spine with spinal cord lesion
35889	7J13200	Closed reduction of fracture of maxilla NEC
36268	S022z00	Fracture of mandible, closed, NOS
36304	S352H00	Closed fracture of cuneiforms
36328	S23xz00	Closed fracture of radius and ulna, NOS
36332	S210600	Closed fracture scapula, neck
36391	S300400	Closed fracture head of femur

Medcode	Read code	Description
36448	S024.11	Fracture of upper jaw, closed
36449	7K1Ez00	Prim open reduction fracture bone & extramedull fixation NOS
36464	S222.00	Closed fracture of humerus, shaft or unspecified part
36497	7K1Jz00	Closed reduction of bone fracture and internal fixation NOS
36527	S4D2100	Closed fracture-subluxation, metacarpophalangeal joint
36556	S261000	Open fracture of phalanx or phalanges, unspecified
36599	S300800	Closed fracture proximal femur, subcapital, Garden grade III
36698	7K1Dz00	Prim open reduction fracture bone & intramedull fixation NOS
36772	S023.11	Fracture of lower jaw, open
36893	7K1LW00	Primary closed reduction of fracture and skin traction
37192	S021.11	Open fracture nasal bone
37291	S3x0.00	Other, multiple and ill-defined closed fractures lower limb
37297	7J12z00	Reduction of fracture of mandible NOS
37310	S3z0.00	Closed fracture of bones, unspecified
37450	S352800	Closed fracture talus, head
37582	S4D1400	Open fracture-dislocation, proximal interphalangeal joint
37609	S01..16	Orbital roof fracture
37662	S310000	Closed fracture of femur, unspecified part
37686	S01..18	Sphenoid bone fracture
37865	S361.00	Open fracture of one or more phalanges of foot
37875	S231A00	Open fracture radius and ulna, proximal
37904	S022x00	Closed fracture of mandible, multiple sites
37945	N331C00	Pathological fracture of cervical vertebra
37986	S261800	Open fracture thumb distal phalanx
38028	S210000	Closed fracture of scapula, unspecified part
38050	S023.00	Fracture of mandible, open
38053	S100711	C7 vertebra closed fracture without spinal cord lesion
38054	S30z.00	Open fracture of neck of femur NOS
38131	7K1LN00	Closed reduction of fracture of upper limb
38353	S220z00	Closed fracture of proximal humerus not otherwise specified
38355	S312500	Closed fracture distal femur, lateral condyle
38395	N331B00	Postmenopausal osteoporosis with pathological fracture
38398	S235600	Open fracture radius and ulna, distal
38408	S4C0100	Closed fracture-dislocation radiocarpal joint
38433	7K1L900	Closed reduction of fracture of metatarsus
38472	7K1Ld00	Primary arthroscopic reduction of fracture
38489	S300.00	Closed fracture proximal femur, transcervical
38590	S4J1.00	Other open fracture-dislocation
38733	S330700	Closed fracture tubercle, tibia
38765	S34y.00	Open fracture ankle, unspecified
38878	S301500	Open fracture proximal femur,subcapital, Garden grade unspec
38895	S132y00	Other specified closed fracture pubis
38943	S4F7.00	Open fracture-subluxation, patello-femoral joint
39071	7206700	Packing of maxilla to correct blow-out fracture of orbit
39321	Q203y12	Fracture of nose due to birth trauma
39334	N331200	Postoophorectomy osteoporosis with pathological fracture
39396	S303400	Open fracture of femur, intertrochanteric
39458	S240900	Closed fracture hamate, hook
39708	S4D0200	Closed fracture-dislocation IPJ, unspecified
39733	S35z.00	Fracture of tarsal and metatarsal bones NOS
39758	S353100	Open fracture of talus
39816	S102000	Closed fracture thoracic vertebra, burst
39859	S04..12	Multiple skull fractures
39887	S100A00	Closed fracture axis, odontoid process
39893	S4H1300	Open fracture-dislocation, metatarsophalangeal joint, single
40069	S331.00	Open fracture of tibia and fibula, proximal
40078	S125100	Closed fracture of hyoid bone
40124	Q202.00	Fracture of clavicle due to birth trauma
40164	S330500	Closed fracture proximal tibia, bicondylar

Medcode	Read code	Description
40267	S4E0.00	Closed fracture-dislocation, hip joint
40268	S234800	Closed Galeazzi fracture
40304	S261K00	Open fracture finger middle phalanx
40321	7K1G300	Primary open reduction of fracture alone
40330	S220600	Closed fracture proximal humerus, three part
40358	S223100	Open fracture of humerus, shaft
40361	S251600	Open fracture finger metacarpal
40367	S224300	Closed fracture distal humerus, medial condyle
40368	S370.00	Closed fracture of lower limb, level unspecified
40394	S125000	Closed fracture larynx
40445	S4H1000	Open fracture-dislocation, subtalar joint
40476	S234500	Closed fracture distal ulna, unspecified
40533	S120200	Closed fracture of two ribs
40535	S260z00	Closed fracture of one or more phalanges of hand NOS
40587	S134500	Closed fracture pelvis, anterior inferior iliac spine
40643	S134000	Closed fracture of ilium, unspecified
40650	S4F6.00	Closed fracture-subluxation, patello-femoral joint
40653	S4F0.00	Closed fracture-dislocation, knee joint
40658	S353H00	Open fracture cuneiforms
40752	S4J..00	Other fracture-dislocation or subluxation
40817	S4D2400	Closed fracture-subluxation, proximal interphalangeal joint
40976	S292000	Closed multiple fractures of clavicle, scapula and humerus
40992	S353200	Open fracture navicular
41138	S102z00	Closed fracture thoracic vertebra not otherwise specified
41287	S320400	Closed fracture patella, comminuted (stellate)
41548	S100400	Closed fracture of fourth cervical vertebra
41558	S4D2000	Closed fracture-subluxation digit, unspecified
41563	7K14z00	Open surgical fracture of bone NOS
41675	S01..13	Frontal sinus fracture
41698	S134300	Closed fracture pelvis, ischial tuberosity
41707	S022500	Closed fracture of mandible, angle of jaw
41730	S022200	Closed fracture of mandible, subcondylar
41760	7K1Lb00	Primary cast immobilisation of fracture
41888	7K1G200	Primary open reduction+external fixation of femoral fracture
41930	S100z00	Closed fracture of cervical spine not otherwise specified
41971	S33xz00	Closed fracture of tibia and fibula, unspecified part, NOS
42076	S234400	Closed fracture of ulna, lower epiphysis
42139	S260B00	Closed fracture thumb distal phalanx, tuft
42149	S100C00	Closed fracture axis, spinous process
42186	7K1Jy00	Closed reduction of bone fracture and internal fixation OS
42561	S100111	C1 vertebra closed fracture - no spinal cord lesion
42780	S105.00	Open fracture lumbar vertebra
42805	S313300	Open fracture distal femur, supracondylar
42844	S4C1.00	Open fracture dislocation wrist
42864	S232100	Closed fracture of the radial shaft
42902	S4H1400	Open fracture-dislocation, IPJ, single toe
42957	S230z00	Closed fracture of proximal forearm not otherwise specified
42968	S104000	Closed fracture lumbar vertebra, burst
42969	S344000	Closed fracture ankle, bimalleolar, low fibular fracture
42972	S311.00	Open fracture of femur, shaft or unspecified part
42978	S330z00	Closed fracture of tibia and fibula, proximal NOS
42990	S4D0000	Closed fracture-dislocation digit, unspecified
43091	S112600	Cls spinal fracture with unspec thoracic cord lesion, T7-12
43153	S352z00	Closed fracture of one or more tarsal + metatarsal bones NOS
43378	S351.00	Open fracture of calcaneus
43423	S4D1300	Open fracture-dislocation, distal interphalangeal joint
43448	S135400	Open fracture pelvis, anterior superior iliac spine
43566	S343000	Open fracture ankle, lateral malleolus, low
43570	S230.00	Closed fracture of proximal radius and ulna

Medcode	Read code	Description
43600	7J42400	Halo skull traction for fracture of spine
43681	S260500	Closed fracture thumb proximal phalanx, shaft
43730	7J03300	Reduction of closed fracture of orbit bone
43786	S11..12	Fracture of vertebra with spinal cord lesion
43792	S24z.00	Fracture of carpal bone NOS
43972	S4B1.00	Open fracture-dislocation elbow
44059	S114.00	Closed fracture of lumbar spine with spinal cord lesion
44142	S225000	Open fracture of elbow, unspecified part
44156	S241400	Open fracture pisiform
44245	S371.00	Open fracture of lower limb, level unspecified
44276	S331300	Open fracture proximal tibia, medial condyle (plateau)
44329	S320200	Closed fracture patella, distal pole
44343	S02A.00	Le Fort I fracture maxilla
44431	S270.00	Closed multiple fractures of hand bones
44538	S230A00	Closed fracture radius and ulna, proximal
44628	S4H0.00	Closed fracture-dislocation foot
44652	S4C2000	Closed fracture-subluxation, distal radio-ulnar jt
44673	S4H2.00	Closed fracture-subluxation, foot
44700	S261E00	Open fracture finger proximal phalanx, base
44711	S200100	Closed fracture clavicle, medial end
44712	S240D00	Closed fracture scaphoid, waist, comminuted
44715	S200000	Closed fracture of clavicle, unspecified part
44721	S220000	Closed fracture of proximal humerus, unspecified part
44737	S260J00	Closed fracture finger proximal phalanx, multiple
44786	S331400	Open fracture proximal tibia, lateral condyle (plateau)
44790	S232200	Closed fracture of the ulnar shaft
44826	S121200	Open fracture of two ribs
44830	S330.00	Closed fracture of tibia and fibula, proximal
44844	S234C00	Closed fracture distal radius, intra-articular, die-punch
44924	S235200	Open fracture of the distal radius, unspecified
44943	S260H00	Closed fracture finger proximal phalanx, head
44949	S02B.00	Le Fort II fracture maxilla
45011	S4J1100	Open fracture-dislocation of pelvis
45094	S260W00	Closed fracture finger distal phalanx, multiple
45141	S302100	Closed fracture proximal femur, intertrochanteric, two part
45275	S221z00	Open fracture of proximal humerus not otherwise specified
45374	S260x00	Closed fractures of phalanx or phalanges, multiple sites
45394	7K1F300	Primary intraarticular fixation intraartic fracture bone NEC
45410	7J41z00	Decompression of fracture of spine NOS
45517	S3x..00	Other, multiple and ill-defined fractures of lower limb
45527	S130z00	Closed fracture acetabulum NOS
45529	S313000	Open fracture distal femur, unspecified
45562	S312400	Closed fracture distal femur, medial condyle
45575	Q203000	Fracture of humerus due to birth trauma
45664	S352A00	Closed fracture talus, body
45690	S4D1500	Open fracture-dislocation, interphalangeal joint thumb
45695	S231.00	Open fracture of proximal radius and ulna
45723	S12z.00	Fracture of rib(s), sternum, larynx or trachea NOS
45779	S300A00	Closed fracture of femur, upper epiphysis
45847	Q203100	Fracture of radius or ulna due to birth trauma
45859	Q203111	Birth fracture of radius
45934	SR11.00	Fractures involving thorax with lower back and pelvis
46142	S04..00	Multiple fractures involving skull or face with other bones
46171	7J42700	Primary collar stabilisation of spinal fracture
46592	S132200	Closed fracture pelvis, multiple pubic rami - unstable
46798	S4C3100	Open fracture-subluxation radiocarpal joint
46894	N331500	Drug-induced osteoporosis with pathological fracture
46955	S352900	Closed fracture talus, neck
47478	S280.00	Closed ill-defined fractures of upper limb

Medcode	Read code	Description
47690	Q203.11	Other fractures due to birth trauma
47828	S347000	Open fracture ankle, trimalleolar, low fibular fracture
47837	S240C00	Closed fracture scaphoid, waist, oblique
47839	S225z00	Open fracture of distal humerus, not otherwise specified
47842	S000.00	Closed fracture vault of skull without intracranial injury
47847	S251z00	Open fracture of metacarpal bone(s) NOS
48142	S313200	Open fracture of femur, lower epiphysis
48159	7J42.11	Other reduction of fracture of spine and stabilisation
48217	S225600	Open fracture distal humerus, lateral epicondyle
48219	7K1LZ00	Primary skin traction of fracture
48224	S121.00	Open fracture rib
48239	S221300	Open fracture proximal humerus, greater tuberosity
48245	S233300	Open fracture radius and ulna, middle
48337	S302012	Closed fracture of femur, lesser trochanter
48636	S025.00	Fracture of malar or maxillary bones, open
48772	N331A00	Osteoporosis + pathological fracture cervical vertebrae
48837	S261S00	Open fracture finger distal phalanx, base
48859	S210500	Closed fracture scapula, spine
48874	S4D2.00	Closed fracture-subluxation digit
48886	S102400	Closed fracture thoracic vertebra, transverse process
48925	S353z00	Open fracture of tarsal and metatarsal bones NOS
48958	S112100	Cls spinal fracture wth complete thoracic cord lesion,T1-6
48961	S223000	Open fracture of humerus NOS
49209	S300y00	Closed fracture proximal femur, other transcervical
49220	Q203y11	Fracture due to birth trauma NEC
49256	S4C1100	Open fracture-dislocation radiocarpal joint
49267	S28..00	Ill-defined fractures of upper limb
49526	S320000	Closed fracture patella, transverse
49529	7J42200	Manipulative reduction of fracture of spine
49564	7J42000	Open reduction of fracture of spine & excis facet of spine
49567	S114000	Closed spinal fracture with unspecified lumbar cord lesion
49588	S241E00	Open fracture scaphoid, tuberosity
49595	S313100	Open fracture of femoral condyle, unspecified
49598	S250B00	Closed fracture thumb metacarpal neck
49644	S024z00	Fracture of malar or maxillary bones, closed, NOS
49796	S235500	Open fracture distal ulna - other
49798	S331700	Open fracture tubercle, tibia
49801	S331012	Open fracture of tibial tuberosity
49821	S353300	Open fracture cuboid
49840	S02y000	Fracture of alveolus, open
49847	S352600	Closed fracture lateral cuneiform
49870	7K1G000	Prmy open reduction of fracture and skeletal traction
50053	S234A00	Closd dorsal Barton's fracture
50148	S4C2100	Closed fracture-subluxation radiocarpal joint
50223	S231000	Open fracture of proximal forearm, unspecified
50227	S321000	Open fracture patella, transverse
50247	S04z.00	Multiple fractures involving skull/face with other bones NOS
50254	S321400	Open fracture patella, comminuted (stellate)
50270	SR1z.00	Multiple fractures, unspecified
50460	7K1Fz00	Primary open reduction of intraarticular fracture bone NOS
50517	S361000	Open fracture proximal phalanx, toe
50549	S320100	Closed fracture patella, proximal pole
50553	Q203200	Fracture of femur due to birth trauma
50573	S4A1100	Open fracture-dislocation acromio-clavicular joint
50634	S250A00	Closed fracture thumb metacarpal shaft
50654	S23x000	Closed fracture of forearm, unspecified
50749	S133.00	Open fracture of pubis
50781	S261z00	Open fracture of one or more phalanges of hand NOS
51018	S118.00	Closed fracture of coccyx with spinal cord lesion

Medcode	Read code	Description
51038	S133100	Open fracture pelvis, multiple pubic rami - stable
51147	7J13500	Reduction of Le Fort 2 fracture of maxilla
51170	S313.00	Open fracture distal femur
51213	S361200	Open fracture distal phalanx, toe
51299	S003.00	Open fracture vault of skull with intracranial injury
51364	S232000	Closed fracture of radius, shaft, unspecified
51392	7J13100	Open reduction of fracture of maxilla NEC
51521	7J42600	Primary bedrest stabilisation of spinal fracture
51666	S241300	Open fracture triquetral
51700	S261U00	Open fracture finger distal phalanx, tuft
51861	S300311	Closed fracture, base of neck of femur
51938	S333100	Open fracture shaft of fibula
51946	S4D3300	Open fracture-subluxation, distal interphalangeal joint
51999	S301900	Open fracture proximal femur,subcapital, Garden grade IV
52067	S253.00	Open fracture sesamoid bone of hand
52083	S224500	Closed fracture of distal humerus, trochlea
52194	S300300	Closed fracture proximal femur, basicervical
52300	S110.00	Closed fracture of cervical spine with cord lesion
52305	S251300	Open fracture finger metacarpal shaft
52318	S310z00	Closed fracture of shaft or unspecified part, NOS
52322	S330900	Closed fracture fibula, neck
52333	S261T00	Open fracture finger distal phalanx, shaft
52340	S4H1.00	Open fracture-dislocation, foot
52346	S346100	Closed fracture ankle, trimalleolar, high fibular fracture
52371	S344100	Closed fracture ankle, bimalleolar, high fibular fracture
52389	S234111	Smith's fracture - closed
52398	S260V00	Closed fracture finger distal phalanx, mallet
52406	S220500	Closed fracture of humerus, upper epiphysis
52457	Syu4300	[X]Fracture of other parts of shoulder and upper arm
52470	S134700	Closed vertical fracture of ilium
52499	S330800	Closed fracture fibula, head
52588	S251400	Open fracture finger metacarpal neck
52614	S231200	Open fracture of ulna, coronoid
52699	S100311	C3 vertebra closed fracture without spinal cord lesion
52895	S250700	Closed fracture finger metacarpal, multiple
52977	Syu5400	[X]Fracture of forearm, unspecified
53007	Zw01.00	[Q] Fractures involving the epiphyseal plate
53068	Syu6500	[X]Fracture of other & unspecified parts of wrist and hand
53279	S312000	Closed fracture of distal femur, unspecified
53337	S100H00	Closed fracture cervical vertebra, wedge
53348	7K1Fy00	Primary open reduction of intraarticular fracture bone OS
53423	SR12.00	Fractures involving multiple regions of one upper limb
53566	S131.00	Open fracture acetabulum
53575	7J02300	Repair of fracture of cranium NEC
53593	S261B00	Open fracture thumb distal phalanx, tuft
53622	S221100	Open fracture proximal humerus, neck
53650	S250C00	Closed fracture thumb metacarpal head
53677	S224900	Closed fracture distal humerus, bicondylar (T-Y fracture)
53688	S221000	Open fracture of proximal humerus, unspecified part
53689	S234911	Closed volar Barton's fracture-dislocation
53693	S251500	Open fracture finger metacarpal head
53698	S235D00	Open fracture distal radius, extra-articular other type
53866	Syu4400	[X]Fracture of shoulder and upper arm, unspecified
53905	S360300	Closed fracture multiple phalanges, toe
53923	S27z.00	Multiple fractures of hand bones NOS
53951	S330011	Closed fracture of tibial condyles
53976	S101200	Open fracture axis
54123	S261W00	Open fracture finger distal phalanx, multiple
54145	S331200	Open fracture of tibia and fibula, proximal

Medcode	Read code	Description
54149	S4B1000	Open fracture-dislocation elbow joint
54242	S312600	Closed fracture distal femur, bicondylar (T-Y fracture)
54280	S330200	Closed fracture of tibia and fibula, proximal
54299	S100K00	Closed fracture cervical vertebra, spinous process
54353	S12X.00	Fracture of bony thorax, part unspecified
54553	S023x00	Open fracture of mandible, multiple sites
54567	Q203400	Fracture of skull due to birth trauma
54660	S320300	Closed fracture patella, vertical
54780	S23y.00	Open fracture of radius and ulna, unspecified part
54834	N331700	Fracture of bone in neoplastic disease
54855	S125300	Closed fracture of trachea
55077	7K1LC00	Closed reduction of fracture of lower limb
55195	S11z.00	Fracture of spine with spinal cord lesion NOS
55201	S231z00	Open fracture of forearm, upper end, NOS
55212	S4C0600	Closed fracture-dislocation peri-lunate trans-scaphoid
55280	S109.00	Open fracture pelvis, coccyx
55308	7K1L011	Manipulation of fracture and skeletal traction NEC
55327	S312x00	Closed fracture distal femur, comminuted/intra-articular
55346	S100x00	Multiple closed fractures of cervical vertebrae
55356	S4D2300	Closed fracture-subluxation, distal interphalangeal joint
55412	S4C2400	Closed fracture-subluxation lunate (volar)
55424	S120500	Closed fracture of five ribs
55464	S332z00	Closed fracture of tibia and fibula, shaft, NOS
55531	S02x200	Fracture of palate, closed
55627	S101.00	Open fracture of cervical spine
55687	S211100	Open fracture scapula, acromion
55814	S241A00	Open fracture scaphoid, proximal pole
55930	7K6FE00	Primary open reduction of fracture dislocation alone
55939	S353700	Open fracture metatarsal
55955	S023500	Open fracture of mandible, angle of jaw
56311	S241500	Open fracture trapezium
56384	S120300	Closed fracture of three ribs
56525	S346000	Closed fracture ankle, trimalleolar, low fibular fracture
56599	S4H0000	Closed fracture-dislocation, subtalar joint
56886	S240y00	Closed fracture of other carpal bone
56927	S4G1.00	Open fracture-dislocation, ankle joint
56961	S12..00	Fracture of rib(s), sternum, larynx and trachea
57190	S022700	Closed fracture of mandible, alveolar border of body
57196	S4F3.00	Open fracture-subluxation, knee joint
57223	SR16000	Closed fracture inv thorax wth low back and pelvis and limbs
57246	S002.00	Open fracture vault of skull without intracranial injury
57301	NyuB000	[X]Other osteoporosis with pathological fracture
57328	S03z.00	Skull fracture NOS
57439	S331z00	Open fracture of tibia and fibula, proximal NOS
57444	S116.00	Closed fracture of sacrum with spinal cord lesion
57592	S210z00	Closed fracture of scapula NOS
57644	S030.00	Closed fracture of skull NOS without intracranial injury
57736	S234A11	Closed dorsal Barton's fracture-dislocation
57893	7K1LT00	Primary closed reduction of fracture and cast immobilisation
57923	S130300	Closed fracture acetabulum, posterior column
57924	S350000	Closed fracture calcaneus, extra-articular
57979	S240000	Closed fracture of carpal bone, unspecified
57981	S14z.00	Fracture of ill-defined bone of trunk NOS
58065	S352500	Closed fracture intermediate cuneiform
58190	S12X000	Closed fracture of bony thorax part unspecified
58642	S30x.00	Open fracture of unspecified proximal femur
58720	S4E1.00	Open fracture-dislocation, hip joint
58752	S4B2.00	Closed fracture-subluxation elbow
59006	S022800	Closed fracture of mandible, body, other and unspecified

Medcode	Read code	Description
59219	S4C3.00	Open fracture-subluxation of the wrist
59233	S02y.00	Open fracture other facial bone
59341	S022300	Closed fracture of mandible, coronoid process
59411	S4G2.00	Closed fracture-subluxation, ankle joint
59798	S01..11	Anterior fossa fracture
59904	S130y00	Other specified closed fracture acetabulum
59943	S221500	Open fracture of humerus, upper epiphysis
59985	S241C00	Open fracture scaphoid, waist, oblique
59996	S101711	C7 vertebra open fracture without spinal cord lesion
60108	S211300	Open fracture scapula, glenoid
60163	S225200	Open fracture distal humerus, lateral condyle
60239	S023z00	Fracture of mandible, open, NOS
60254	7J12000	Reduction of fracture of alveolus of mandible
60260	S023800	Open fracture of mandible, body, other and unspecified
60343	S4C3000	Open fracture-subluxation, distal radio-ulnar joint
60352	7J43z00	Fixation of fracture of spine NOS
60382	S101311	C3 vertebra open fracture without spinal cord lesion
60412	7J13600	Reduction of Le Fort 3 fracture of maxilla
60487	S4D2200	Closed fracture-subluxation IPJ, unspecified
60518	S233000	Open fracture of radius, shaft, unspecified
60580	S4A2000	Closed fracture-subluxation shoulder joint
60593	S100300	Closed fracture of third cervical vertebra
60608	S140.00	Closed fracture of ill-defined bone of trunk
60615	S113.00	Open fracture of thoracic spine with spinal cord lesion
60630	S235000	Open fracture of forearm, lower end, unspecified
60633	S023100	Open fracture of mandible, condylar process
60669	S4F5.00	Open fracture-dislocation, patello-femoral joint
60765	S251800	Open fracture of thumb metacarpal
60828	S4A3100	Open fracture-subluxation acromio-clavicular joint
60885	S301600	Open fracture proximal femur,subcapital, Garden grade I
61150	S104200	Closed fracture lumbar vertebra, spondylolysis
61181	S260C00	Closed fracture thumb distal phalanx, mallet
61279	S261300	Open fracture thumb proximal phalanx
61374	S231500	Open fracture of the proximal ulna
61378	S222z00	Closed fracture of humerus, shaft or unspecified part NOS
61388	S03..00	Other and unqualified skull fractures
61389	7J42M00	Primary cls reduc spinal fracture+skull traction stabilisatn
61491	7J43400	Primary open reduc spinal fracture+internal fix+rod system
61529	S261H00	Open fracture finger proximal phalanx, head
61556	S361300	Open fracture multiple phalanges, toe
61653	7K6GX00	Primary closed reduction of fracture dislocation alone
61675	S235900	Open volar Barton's fracture
61733	S303.00	Open fracture of proximal femur, pertrochanteric
61757	S01..17	Posterior fossa fracture
61802	S312z00	Closed fracture of distal femur not otherwise specified
61812	S201.00	Open fracture of clavicle
61858	S4D1100	Open fracture-dislocation, metacarpophalangeal joint
62047	S103100	Open fracture thoracic vertebra, wedge
62334	S261F00	Open fracture finger proximal phalanx, shaft
62337	S110000	Cls spinal fracture with unspec cervical cord lesion, C1-4
62343	N331z00	Pathological fracture NOS
62354	Q203300	Fracture of tibia or fibula due to birth trauma
62489	7J43.11	Internal fixation of fracture of spine
62562	S131y00	Other specified open fracture acetabulum
62631	S4J2000	Closed fracture-subluxation of sternum
62649	SP04C00	Fracture of bone allograft
62716	S022.11	Fracture of inferior maxilla, closed
62719	S101611	C6 vertebra open fracture without spinal cord lesion
62787	S33yz00	Open fracture of tibia and fibula, unspecified part, NOS

Medcode	Read code	Description
62808	S260A00	Closed fracture thumb distal phalanx, shaft
62833	S224x00	Closed fracture of distal humerus, multiple
62853	S251000	Open fracture of metacarpal bone(s), site unspecified
62960	S4B0100	Closed fracture-dislocation superior radio-ulnar joint
62966	S300z00	Closed fracture proximal femur, transcervical, NOS
62977	S010.00	Closed fracture base of skull without intracranial injury
63049	S4D0600	Closed fracture-dislocation multiple digits
63064	7J03y00	Other specified reduction of fracture of facial bone
63071	S4D2500	Closed fracture-subluxation, interphalangeal joint thumb
63085	7K1G500	Primary open reduction of fracture and functional bracing
63242	S14..00	Fracture of ill-defined bones of trunk
63253	S103.00	Open fracture thoracic vertebra
63292	S4C1600	Open fracture-dislocation peri-lunate trans-scaphoid
63452	7J42300	Spinal extension traction for fracture of spine
63588	S235E00	Open fracture distal radius, intra-articular other type
63633	S331600	Open fracture spine, tibia
63679	S011.00	Closed fracture base of skull with intracranial injury
63712	S4C2600	Closed fracture-subluxation peri-lunate trans-scaphoid
63899	S225500	Open fracture of distal humerus, trochlea
63948	S231900	Open fracture of the proximal radius
63954	7J43211	Barr skull traction for fracture of spine
63980	7J43200	Fixation of fracture of spine and skull traction HFQ
63982	S123.00	Open fracture sternum
64021	S211200	Open fracture scapula, coracoid
64027	S260600	Closed fracture thumb proximal phalanx, neck
64113	S261G00	Open fracture finger proximal phalanx, neck
64139	S13z.00	Open fracture of pelvis NOS
64222	7K1Kz00	Closed reduction of bone fracture and external fixation NOS
64229	S4A1.00	Open fracture-dislocation shoulder
64297	S100L00	Closed fracture cervical vertebra, transverse process
64378	S352G00	Closed tarsal fractures, multiple
64388	7J43300	Primary open reduc spinal fracture+internal fix+wire
64435	S4C0400	Closed fracture-dislocation lunate (volar)
64545	S353C00	Open fracture metatarsal shaft
64575	S241D00	Open fracture scaphoid, waist, comminuted
64725	S241700	Open fracture capitate
64777	S131z00	Open fracture acetabulum NOS
64862	7K1K000	Closed reduction fracture bone and fixation to skeleton HFQ
64872	S102300	Closed fracture thoracic vertebra, spinous process
65028	S4H2100	Closed fracture-subluxation, midtarsal joint
65084	S135.00	Other or multiple open fracture of pelvis
65141	S241.00	Open fracture of carpal bone
65155	S4J0000	Closed fracture-dislocation of sternum
65228	S335100	Open fracture distal tibia, intra-articular
65297	S4J3100	Open fracture-subluxation of pelvis
65300	S101600	Open fracture of sixth cervical vertebra
65301	S23yz00	Open fracture of radius and ulna, NOS
65302	S105100	Open fracture lumbar vertebra, wedge
65484	S120600	Closed fracture of six ribs
65494	S4D3400	Open fracture-subluxation, proximal interphalangeal joint
65606	7J41y00	Other specified decompression of fracture of spine
65636	S234912	Closed volar Barton fracture-subluxation
65690	S300200	Closed fracture proximal femur, midcervical section
65715	S261A00	Open fracture thumb distal phalanx, shaft
65731	S261900	Open fracture thumb distal phalanx, base
65848	S4D1000	Open fracture-dislocation digit, unspecified
65895	S4H3300	Open fracture-subluxation, metatarsophalangeal joint, single
66099	S353F00	Open fracture metatarsal, multiple
66164	S10y.00	Open fracture of spine, unspecified,

Medcode	Read code	Description
66231	S353400	Open fracture medial cuneiform
66233	S233100	Open fracture of the radial shaft
66237	S292100	Open multiple fractures of clavicle, scapula and humerus
66322	S150100	Open multiple fracture of thoracic spine
66434	S107.00	Open fracture sacrum
66544	S4D3100	Open fracture-subluxation, metacarpophalangeal joint
66774	S235z00	Open fracture of forearm, lower end, NOS
66808	S345000	Open fracture ankle, bimalleolar, low fibular fracture
66853	S281.00	Open ill-defined fractures of upper limb
67007	S353.00	Open fracture of other tarsal and metatarsal bones
67011	S261x00	Open fracture of phalanx or phalanges, multiple sites
67036	S4B2000	Closed fracture-subluxation elbow joint
67097	S260Q00	Closed fracture finger middle phalanx, multiple
67239	S353J00	Open fracture of base of fifth metatarsal
67294	S313400	Open fracture distal femur, medial condyle
67358	S100411	C4 vertebra closed fracture without spinal cord lesion
67394	S301700	Open fracture proximal femur,subcapital, Garden grade II
67584	S4C0200	Closed fracture-dislocation mid carpal
67603	S033.00	Open fracture of skull NOS with intracranial injury
67669	S135600	Open fracture pelvis, iliac wing
67718	S261L00	Open fracture finger middle phalanx, base
67849	S4H0200	Closed fracture-dislocation, tarsometatarsal joint
67973	S100G00	Closed fracture cervical vertebra, burst
68019	N331400	Postsurgical malabsorption osteoporosis with path fracture
68085	S241000	Open fracture of carpal bone, unspecified
68229	S300y11	Closed fracture of femur, subcapital
68262	S4C3600	Open fracture-subluxation peri-lunate trans-scaphoid
68514	S4H3400	Open fracture-subluxation, IPJ, single toe
68556	S201300	Open fracture clavicle, lateral end
68595	S4C2300	Closed fracture-subluxation, carpometacarpal joint
68652	S120800	Closed fracture of eight or more ribs
68660	S023400	Open fracture of mandible, ramus, unspecified
68668	S301y00	Open fracture proximal femur, other transcervical
68763	S135300	Open fracture pelvis, ischial tuberosity
68765	S241200	Open fracture lunate
68811	7J41100	Anterior decompression of fracture of spine
68899	S29..13	Multiple fractures of sternum
68940	S025z00	Fracture of malar or maxillary bones, open, NOS
68981	S02C.00	Le Fort III fracture maxilla
69098	S101111	C1 vertebra open fracture without spinal cord lesion
69213	S241800	Open fracture hamate
69312	S225300	Open fracture distal humerus, medial condyle
69319	7J13z00	Reduction of fracture of maxilla NOS
69362	7K6H411	Remanipulation of fracture dislocation alone
69363	S261N00	Open fracture finger middle phalanx, neck
69418	S130400	Closed fracture acetabulum, floor
69432	S111.00	Open fracture of cervical spine with spinal cord lesion
69496	SD92000	Fracture blister
69551	SP04A00	Fracture of bone autograft
69645	S101100	Open fracture atlas
69702	7K1K700	Primary functional bracing of fracture
69728	S361100	Open fracture middle phalanx, toe
69729	S261J00	Open fracture finger proximal phalanx, multiple
69737	S012.00	Open fracture base skull without mention intracranial injury
69786	Zw02400	[Q] Stress fracture
69824	SR13.00	Fractures involving multiple regions of one lower limb
69917	S3x1.00	Other, multiple and ill-defined open fractures of lower limb
69919	S300100	Closed fracture proximal femur, transepiphyseal
69974	S100900	Closed fracture atlas, comminuted

Medcode	Read code	Description
70226	S351100	Open fractures calcaneus, intra-articular
70282	S025.11	Fracture of upper jaw, open
70479	S303z00	Open fracture of proximal femur, pertrochanteric, NOS
70486	S221400	Open fracture proximal humerus, head
70503	S233200	Open fracture of the ulnar shaft
70590	S23y000	Open fracture of forearm, unspecified
70604	S221600	Open fracture proximal humerus, three part
70653	S221700	Open fracture proximal humerus, four part
70673	S023000	Open fracture mandible (site unspecified)
70674	S133z00	Open fracture of pubis NOS
70864	S201000	Open fracture of clavicle, unspecified part
70919	7K1Ly00	Other specified other closed reduction of fracture of bone
71006	7J42900	Primary cast stabilisation of spinal fracture
71132	S4H0100	Closed fracture-dislocation, midtarsal joint
71207	S221200	Open fracture of proximal humerus, anatomical neck
71282	S303200	Open fracture proximal femur, subtrochanteric
71452	S121900	Open fracture multiple ribs
71567	S125.00	Closed fracture larynx and trachea
71583	S022600	Closed fracture of mandible, symphysis of body
71622	S4J2.00	Other closed fracture-subluxation
71739	Syu6400	[X]Fracture of other metacarpal bone
71953	S211600	Open fracture scapula, neck
72071	S4H2000	Closed fracture-subluxation, subtalar joint
72138	S301100	Open fracture proximal femur, transepiphyseal
72324	S11..11	Fracture of transverse process of spine + spinal cord lesion
72404	S106000	Closed compression fracture sacrum
72407	S251700	Open fracture finger metacarpal, multiple
72408	S231800	Open fracture proximal radius, comminuted
72525	S130000	Closed fracture acetabulum, anterior lip alone
72586	S353B00	Open fracture metatarsal base
72600	S106100	Closed vertical fracture of sacrum
72617	S101x00	Multiple open fractures of cervical vertebrae
72711	S110600	Cls spinal fracture with unspec cervical cord lesion, C5-7
72822	S4H2600	Closed fracture-subluxation, IPJ, multiple toes
72908	7K15y00	Other specified other surgical fracture of bone
73105	S343100	Open fracture ankle, lateral malleolus, high
73109	S211400	Open fracture scapula, blade
73113	Syu7200	[X]Fractures of other parts of femur
73150	S251200	Open fracture finger metacarpal base
73165	S240F00	Closed fracture carpal bones, multiple
73208	S313x00	Open fracture distal femur, comminuted/intra-articular
73210	S301400	Open fracture head, femur
73234	S301y11	Open fracture of femur, subcapital
73336	SR1z000	[X]Closed multiple fractures unspecified
73344	7J43y00	Other specified fixation of fracture of spine
73416	S110z00	Closed fracture of cervical spine with cord lesion NOS
73426	S223z00	Open fracture of humerus, shaft or unspecified part NOS
73479	S130200	Closed fracture acetabulum, anterior column
73601	S105000	Open fracture lumbar vertebra, burst
73611	S112z00	Closed fracture of thoracic spine with cord lesion NOS
73613	S121000	Open fracture of rib, unspecified
73703	S4H2200	Closed fracture-subluxation, tarsometatarsal joint
73768	S211000	Open fracture of scapula, unspecified part
73786	SR10000	Closed fractures involving head with neck
73788	S114500	Closed spinal fracture with cauda equina lesion
73812	7K1N900	Primary skeletal traction of fracture
73824	S241B00	Open fracture scaphoid, waist, transverse
73956	S121700	Open fracture of seven ribs
73981	S301.00	Open fracture proximal femur, transcervical

Medcode	Read code	Description
73986	S4D3.00	Open fracture-subluxation digit
78444	S33A.00	Fracture of tibia
85491	S4B3.00	Open fracture-subluxation elbow
85656	7K6Hh00	Sec open red fracture dislocat joint and intern fixation NEC
86803	S225400	Open fracture of distal humerus, condyle(s) unspecified
88269	7J41300	Vertebroplasty of fracture of spine
88737	S313z00	Open fracture of distal femur not otherwise specified
88784	7J13y00	Other specified reduction of fracture of maxilla
89101	7206800	Internal fixation of fracture of orbit
89434	7P20100	Delivery of rehabilitation for hip fracture
90472	7J41500	Balloon kyphoplasty of fracture of spine
90494	S120700	Closed fracture of seven ribs
91649	7J43700	Primary open reduc spinal fracture+other internal fix
91658	7J41400	Posterior decompression of fracture of spine NEC
91919	7K1G600	Primary open reduction of fracture and skin traction
92043	S4H1200	Open fracture-dislocation, tarsometatarsal joint
92268	S4H0600	Closed fracture-dislocation, IPJ, multiple toes
92349	S4D3500	Open fracture-subluxation, interphalangeal joint thumb
92356	7J42z00	Other reduction of fracture of spine NOS
92830	SR12000	Closed fractures involving multiple regions of one upp limb
93029	S331011	Open fracture of tibial condyles
93374	S4E2.00	Closed fracture-subluxation, hip joint
93497	N331N00	Fragility fracture
93536	S353900	Open fracture talus, neck
93650	S4H3.00	Open fracture-subluxation, foot
93705	N331M11	Minimal trauma fracture due to unspecified osteoporosis
93752	7J42y00	Other specified other reduction of fracture of spine
93915	Q203112	Birth fracture of ulna
93981	N331N11	Minimal trauma fracture
94031	S261400	Open fracture thumb proximal phalanx, base
94108	S126300	Open fracture of trachea
94127	S133y00	Other specified open fracture of pubis
94189	S115.00	Open fracture of lumbar spine with spinal cord lesion
94236	S4J1000	Open fracture-dislocation of sternum
94265	S4D1200	Open fracture-dislocation IPJ, unspecified
94292	S100D00	Closed fracture axis, transverse process
94360	S311z00	Open fracture of femur, shaft or unspecified part, NOS
94393	S251x00	Open fractures of multiple sites of unspecified metacarpus
94416	S261500	Open fracture thumb proximal phalanx, shaft
94435	S211z00	Open fracture of scapula NOS
94460	S201z00	Open fracture of clavicle NOS
94584	S117300	Open fracture of sacrum with other spinal cord injury
94593	S4J3000	Open fracture-subluxation of sternum
94638	Syu1500	[X]Fracture of other specified cervical vertebra
94649	S130100	Closed fracture acetabulum, posterior lip alone
94655	S135800	Open fracture dislocation of sacro-iliac joint
94661	S4C3300	Open fracture-subluxation, carpometacarpal joint
94692	Syu0400	[X]Fracture of skull and facial bones, part unspecified
94844	S101A00	Open fracture axis, odontoid process
95006	S100E00	Closed fracture axis, posterior arch
95040	Zw02.00	[Q] Fracture type qualifying terms
95513	S100M00	Closed fracture cervical vertebra, posterior arch
95529	S114100	Closed spinal fracture with complete lumbar cord lesion
95585	S104600	Closed fracture lumbar vertebra, tricolumnar
95619	Zw02E00	[Q] Open fracture grade 2
95620	S100J00	Closed fracture cervical vertebra, spondylolysis
95633	S294000	Cl fractures involving multiple regions of both upper limbs
95674	N1y2.00	Pars interarticularis stress fracture
95728	S4H2400	Closed fracture-subluxation, IPJ, single toe

Medcode	Read code	Description
95839	S12y000	Closed fracture of other parts of bony thorax
95842	S104500	Closed fracture lumbar vertebra, posterior arch
96136	S4C0500	Closed fracture-dislocation peri-lunate (dorsal)
96438	S4D3600	Open fracture-subluxation multiple digits
96460	SR1z100	[X]Open multiple fractures unspecified
96473	S117.00	Open fracture of sacrum with spinal cord lesion
96514	S110800	Cls spinal fracture with anterior cervcl cord lesion, C5-7
96518	S301A00	Open fracture of femur, upper epiphysis
96643	S126100	Open fracture of hyoid bone
96644	S303011	Open fracture of femur, greater trochanter
96659	S102200	Closed fracture thoracic vertebra, spondylolysis
96691	S235400	Open fracture of ulna, lower epiphysis
96939	Syu8300	[X]Fractures of other parts of lower leg
96984	S135000	Open fracture of ilium, unspecified
96998	S4D1600	Open fracture-dislocation multiple digits
97064	S013.00	Open fracture base of skull with intracranial injury
97111	7K1F400	Prim extraarticular reduction intraartic fracture bone NEC
97120	S101211	C2 vertebra open fracture without spinal cord lesion
97211	S353500	Open fracture intermediate cuneiform
97352	Syu4200	[X]Multiple fractures of clavicle, scapula and humerus
97354	S107100	Open vertical fracture of sacrum
97380	Syu9400	[X]Fracture of other tarsal bones
97386	S353A00	Open fracture talus, body
97476	S4C1300	Open fracture-dislocation carpometacarpal joint
97803	S353D00	Open fracture metatarsal neck
97820	S225900	Open fracture distal humerus, bicondylar (T-Y fracture)
97971	S303300	Open fracture proximal femur, intertrochanteric, comminuted
98165	7J42B00	Primary other external stabilisation of spinal fracture
98267	S130600	Closed fracture acetabulum, double column unspecified
98393	S100800	Closed fracture atlas, isolated arch or articular process
98681	S235111	Smith's fracture - open
98867	S251A00	Open fracture thumb metacarpal shaft
98933	S241600	Open fracture trapezoid
99027	S331800	Open fracture fibula, head
99151	S101511	C5 vertebra open fracture without spinal cord lesion
99161	S331900	Open fracture fibula, neck
99203	S135y00	Other open fracture of pelvis
99303	SR14.00	Fractures involving multiple regions of both lower limbs
99325	S225x00	Open fracture of distal humerus, multiple
99376	S116z00	Closed fracture of sacrum with spinal cord lesion NOS
99397	S261600	Open fracture thumb proximal phalanx, neck
99459	S261P00	Open fracture finger middle phalanx, head
99516	S102500	Closed fracture thoracic vertebra, posterior arch
99549	S023200	Open fracture of mandible, subcondylar
99895	S12y.00	Fracture of other parts of bony thorax
99936	Syu1600	[X]Fracture of other parts of neck
99994	7K1K900	Other primary external immobilisation of fracture
100040	S201100	Open fracture clavicle, medial end
100159	S321100	Open fracture patella, proximal pole
100196	S353000	Open fracture of tarsal bone, unspecified
100202	S33C.00	Closed fracture of distal tibia and fibula
100350	S4C2y00	Closed fracture-subluxation other carpal
100640	S33B.00	Open fracture of distal tibia and fibula
100677	N331.13	Sponanteous fracture
100771	S301311	Open fracture base of neck of femur
101031	S336000	Fracture tibial plateau
101299	S113000	Opn spinal fracture with unspec thoracic cord lesion, T1-6
101316	S261700	Open fracture thumb proximal phalanx, head
101318	S103500	Open fracture thoracic vertebra, posterior arch

Medcode	Read code	Description
101447	S133200	Open fracture pelvis, multiple pubic rami - unstable
101517	S118z00	Closed fracture of coccyx with spinal cord lesion NOS
101560	S121z00	Open fracture of rib(s) NOS
101567	S303100	Open fracture proximal femur, intertrochanteric, two part
101574	S101000	Open fracture of unspecified cervical vertebra
101840	S331A00	Open fracture tibial plateau
102013	S241z00	Open fracture of carpal bone NOS
102043	S112A00	Cls spinal fracture with posterior thorac cord lesion, T7-12
102046	S261V00	Open fracture finger distal phalanx, mallet
102155	Syu6300	[X]Fracture of other carpal bone(s)
102225	S251C00	Open fracture thumb metacarpal head
102302	S234G00	Greenstick fracture of distal radius
102735	S110700	Cls spinal fracture with complete cervcl cord lesion, C5-7
102916	Syu5300	[X]Fracture of other parts of forearm
102965	S4A1000	Open fracture-dislocation shoulder joint
103024	Syu8D00	[X]Fracture of lower leg, part unspecified
103049	SR15000	Cl fractures involving multiple regions upper with lower lmb
103416	S261C00	Open fracture thumb distal phalanx, mallet
103428	SP22400	Intra-operative fracture
103524	S4C2200	Closed fracture-subluxation mid carpal
104015	S4D2600	Closed fracture-subluxation multiple digits
104066	7K1Y100	Remanip fracture long bone and rigid internal fixation NEC
104067	7K1L211	Remanipulation of fracture and skeletal traction NEC
104070	S235C00	Open fracture distal radius, intra-articular, die-punch
104183	Q204100	Spine fracture due to birth trauma
104355	S4B1100	Open fracture-dislocation superior radio-ulnar joint
104356	S4B2100	Closed fracture-subluxation superior radio-ulnar joint
104738	S02y200	Fracture of palate, open
104755	S113A00	Opn spinal fracture with posterior thorac cord lesion, T7-12
104803	7J42L00	Primary cls reduction spinal fracture+bedrest stabilisation
104931	S023600	Open fracture of mandible, symphysis of body
105278	S235211	Dupuytren's fracture, radius - open
105612	S353E00	Open fracture metatarsal head
105695	S105400	Open fracture lumbar vertebra, transverse process
105702	S100B00	Closed fracture axis, spondylolysis
105816	S345100	Open fracture ankle, bimalleolar, high fibular fracture
105819	S347100	Open fracture ankle, trimalleolar, high fibular fracture
105935	S107000	Open compression fracture sacrum
106283	S023700	Open fracture of mandible, alveolar border of body
106504	7J41000	Complex decompression of fracture of spine
106875	S4H1600	Open fracture-dislocation, IPJ, multiple toes
107741	S234A12	Closed dorsal Barton fracture-subluxation
108000	S135100	Open fracture pelvis, ischium
108242	S353600	Open fracture lateral cuneiform
108484	S112000	Cls spinal fracture with unspec thoracic cord lesion,T1-6
108596	S4H1100	Open fracture-dislocation, midtarsal joint
109115	S353800	Open fracture talus, head
109377	S110100	Cls spinal fracture with complete cervcl cord lesion, C1-4
109469	SyuBB00	[X]Fracture of unspecified body region
109772	Syu0300	[X]Fractures of other skull and facial bones
110205	SyuA200	[X]Fractures involving other combinations of body regions
110247	7J42J00	Primary closed reduction spinal fracture alone
110732	S101900	Open fracture atlas, comminuted
110906	S241900	Open fracture hamate, hook
111042	Syu2800	[X]Fracture of bony thorax, part unspecified
111060	S4A3.00	Open fracture-subluxation shoulder
111125	S4J3.00	Other open fracture-subluxation
111268	Zw02D00	[Q] Open fracture grade 1
111858	S235A00	Open dorsal Barton's fracture

Medcode	Read code	Description
112187	S135500	Open fracture pelvis, anterior inferior iliac spine
112577	S4H3200	Open fracture-subluxation, tarsometatarsal joint
112595	S023300	Open fracture of mandible, coronoid process
112617	S100.11	Closed fracture of atlas without spinal cord lesion
112701	S121100	Open fracture of one rib
112709	S126200	Open fracture of thyroid cartilage
112712	S301z00	Open fracture proximal femur, transcervical, NOS

ICD-10 Codes for fracture used in HES and ONS

ICD Code	Description
S02	Fracture of skull and facial bones
S02.0	Fracture of vault of skull
S02.1	Fracture of base of skull
S02.2	Fracture of nasal bones
S02.3	Fracture of orbital floor
S02.4	Fracture of malar and maxillary bones
S02.6	Fracture of mandible
S02.7	Multiple fractures involving skull and facial bones
S02.8	Fractures of other skull and facial bones
S02.9	Fracture of skull and facial bones, part unspecified
S12	Fracture of neck
S12.0	Fracture of first cervical vertebra
S12.1	Fracture of second cervical vertebra
S12.2	Fracture of other specified cervical vertebra
S12.7	Multiple fractures of cervical spine
S12.8	Fracture of other parts of neck
S12.9	Fracture of neck, part unspecified
S22	Fracture of rib(s), sternum and thoracic spine
S22.0	Fracture of thoracic vertebra
S22.1	Multiple fractures of thoracic spine
S22.2	Fracture of sternum
S22.3	Fracture of rib
S22.4	Multiple fractures of ribs
S22.5	Flail chest
S22.8	Fracture of other parts of bony thorax
S22.9	Fracture of bony thorax, part unspecified
S32	Fracture of lumbar spine and pelvis
S32.0	Fracture of lumbar vertebra
S32.1	Fracture of sacrum
S32.2	Fracture of coccyx
S32.3	Fracture of ilium
S32.4	Fracture of acetabulum
S32.5	Fracture of pubis
S32.7	Multiple fractures of lumbar spine and pelvis
S32.8	Fracture of other and unspecified parts of lumbar spine and pelvis
S42	Fracture of shoulder and upper arm
S42.0	Fracture of clavicle

ICD Code	Description
S42.1	Fracture of scapula
S42.2	Fracture of upper end of humerus
S42.3	Fracture of shaft of humerus
S42.4	Fracture of lower end of humerus
S42.7	Multiple fractures of clavicle, scapula and humerus
S42.8	Fracture of other parts of shoulder and upper arm
S42.9	Fracture of shoulder girdle, part unspecified
S52	Fracture of forearm
S52.0	Fracture of upper end of ulna
S52.1	Fracture of upper end of radius
S52.2	Fracture of shaft of ulna
S52.3	Fracture of shaft of radius
S52.4	Fracture of shafts of both ulna and radius
S52.5	Fracture of lower end of radius
S52.6	Fracture of lower end of both ulna and radius
S52.7	Multiple fractures of forearm
S52.8	Fracture of other parts of forearm
S52.9	Fracture of forearm, part unspecified
S62	Fracture at wrist and hand level
S62.0	Fracture of navicular [scaphoid] bone of hand
S62.1	Fracture of other carpal bone(s)
S62.2	Fracture of first metacarpal bone
S62.3	Fracture of other metacarpal bone
S62.4	Multiple fractures of metacarpal bones
S62.5	Fracture of thumb
S62.6	Fracture of other finger
S62.7	Multiple fractures of fingers
S62.8	Fracture of other and unspecified parts of wrist and hand
S72	Fracture of femur
S72.0	Fracture of neck of femur
S72.1	Pertrochanteric fracture
S72.2	Subtrochanteric fracture
S72.3	Fracture of shaft of femur
S72.4	Fracture of lower end of femur
S72.7	Multiple fractures of femur
S72.8	Fractures of other parts of femur
S72.9	Fracture of femur, part unspecified
S82	Fracture of lower leg, including ankle
S82.0	Fracture of patella
S82.1	Fracture of upper end of tibia
S82.2	Fracture of shaft of tibia
S82.3	Fracture of lower end of tibia
S82.4	Fracture of fibula alone
S82.5	Fracture of medial malleolus
S82.6	Fracture of lateral malleolus
S82.7	Multiple fractures of lower leg
S82.8	Fractures of other parts of lower leg

ICD Code	Description
S82.9	Fracture of lower leg, part unspecified
S92	Fracture of foot, except ankle
S92.0	Fracture of calcaneus
S92.1	Fracture of talus
S92.2	Fracture of other tarsal bone(s)
S92.3	Fracture of metatarsal bone
S92.4	Fracture of great toe
S92.5	Fracture of other toe
S92.7	Multiple fractures of foot
S92.9	Fracture of foot, unspecified
M80	Osteoporosis with pathological fracture
M80.0	Postmenopausal osteoporosis with pathological fracture
M80.1	Postoophorectomy osteoporosis with pathological fracture
M80.2	Osteoporosis of disuse with pathological fracture
M80.3	Postsurgical malabsorption osteoporosis with pathological fracture
M80.4	Drug-induced osteoporosis with pathological fracture
M80.5	Idiopathic osteoporosis with pathological fracture
M80.8	Other osteoporosis with pathological fracture
M80.9	Unspecified osteoporosis with pathological fracture
M84.3	Stress fracture, not elsewhere classified
M84.4	Pathological fracture, not elsewhere classified
M84.8	Other disorders of continuity of bone
M84.9	Disorder of continuity of bone, unspecified
T02	Fractures involving multiple body regions
T02.0	Fractures involving head with neck
T02.1	Fractures involving thorax with lower back and pelvis
T02.2	Fractures involving multiple regions of one upper limb
T02.3	Fractures involving multiple regions of one lower limb
T02.4	Fractures involving multiple regions of both upper limbs
T02.5	Fractures involving multiple regions of both lower limbs
T02.6	Fractures involving multiple regions of upper limb(s) with lower limb(s)
T02.7	Fractures involving thorax with lower back and pelvis with limb(s)
T02.8	Fractures involving other combinations of body regions
T02.9	Multiple fractures, unspecified
T08	Fracture of spine, level unspecified
T10	Fracture of upper limb, level unspecified
T12	Fracture of lower limb, level unspecified
T14.2	Fracture of unspecified body region

Read codes for peptic ulcer used in CPRD

Medcode	Read code	Description
352	J12..00	Duodenal ulcer - (DU)
657	J12y200	Unspecified duodenal ulcer with perforation
670	J13..00	Peptic ulcer - (PU) site unspecified
1262	J11..00	Gastric ulcer - (GU)
1295	J14..15	Stomal ulcer
3101	J11..12	Pyloric ulcer
3462	J123.00	Duodenal erosion
4741	7627000	Closure of perforated duodenal ulcer
5521	J130200	Acute peptic ulcer with perforation
5928	7627	Operations on duodenal ulcer
6333	J11..11	Prepyloric ulcer
6865	761J.11	Stomach ulcer operations
9853	J121.00	Chronic duodenal ulcer
11104	J111211	Perforated chronic gastric ulcer
11124	J110111	Bleeding acute gastric ulcer
14671	J110200	Acute gastric ulcer with perforation
15175	J12z.00	Duodenal ulcer NOS
15403	J14z.00	Gastrojejunal ulcer NOS
15821	J13..11	Stress ulcer NOS
15979	J14y200	Unspecified gastrojejunal ulcer with perforation
18001	J120100	Acute duodenal ulcer with haemorrhage
18027	J120.00	Acute duodenal ulcer
18324	J120200	Acute duodenal ulcer with perforation
18625	J121111	Bleeding chronic duodenal ulcer
18654	J111.00	Chronic gastric ulcer
19928	J13z.00	Peptic ulcer NOS
20677	761J000	Closure of perforated gastric ulcer
22918	J122.00	Duodenal ulcer disease
23082	J14..00	Gastrojejunal ulcer (GJU)
23688	761J.00	Operations on gastric ulcer
24021	J102000	Peptic ulcer of oesophagus
24040	J110.00	Acute gastric ulcer
24342	J110y00	Acute gastric ulcer unspecified
26261	ZV12711	[V]Personal history of peptic ulcer
28366	J12yy00	Unspec duodenal ulcer; unspec haemorrhage and/or perforation
29317	J124.00	Recurrent duodenal ulcer
29771	J11z.00	Gastric ulcer NOS
30054	J110100	Acute gastric ulcer with haemorrhage
32856	J130.00	Acute peptic ulcer
33438	J121000	Chronic duodenal ulcer without mention of complication
33914	761Jz00	Operation on gastric ulcer NOS
36461	J11y200	Unspecified gastric ulcer with perforation
36583	J111111	Bleeding chronic gastric ulcer
37620	J131200	Chronic peptic ulcer with perforation
40997	J131.00	Chronic peptic ulcer
41271	ZV12C00	[V] Personal history of gastric ulcer
42274	J140z00	Acute gastrojejunal ulcer NOS
44073	J121400	Chronic duodenal ulcer with obstruction
44284	J11yz00	Unspecified gastric ulcer NOS
44309	J111z00	Chronic gastric ulcer NOS
44324	J110z00	Acute gastric ulcer NOS
44335	J120000	Acute duodenal ulcer without mention of complication
44637	J130100	Acute peptic ulcer with haemorrhage
45184	J140.00	Acute gastrojejunal ulcer
45304	J130300	Acute peptic ulcer with haemorrhage and perforation
48730	J120300	Acute duodenal ulcer with haemorrhage and perforation
48946	J111000	Chronic gastric ulcer without mention of complication
50048	J130z00	Acute peptic ulcer NOS

Medcode	Read code	Description
50497	J13y.00	Unspecified peptic ulcer
51406	J121z00	Chronic duodenal ulcer NOS
52138	J121y00	Chronic duodenal ulcer unspecified
52313	J131400	Chronic peptic ulcer with obstruction
52323	J11z.12	Multiple gastric ulcers
53081	J11y.00	Unspecified gastric ulcer
53126	J131100	Chronic peptic ulcer with haemorrhage
53336	J111200	Chronic gastric ulcer with perforation
53669	J12y.00	Unspecified duodenal ulcer
53797	J120z00	Acute duodenal ulcer NOS
53822	J120y00	Acute duodenal ulcer unspecified
57958	J11y100	Unspecified gastric ulcer with haemorrhage
60249	J13yz00	Unspecified peptic ulcer NOS
60346	J14y100	Unspecified gastrojejunal ulcer with haemorrhage
63001	J112.00	Anti-platelet induced gastric ulcer
63482	J14y.00	Unspecified gastrojejunal ulcer
63582	J111100	Chronic gastric ulcer with haemorrhage
63718	761D600	Endoscopic injection haemostasis of gastric ulcer
64014	761J100	Closure of gastric ulcer NEC
64111	J13y200	Unspecified peptic ulcer with perforation
64165	J110000	Acute gastric ulcer without mention of complication
64556	J111y00	Chronic gastric ulcer unspecified
64710	7612500	Resection of gastric ulcer by cautery
64913	J14..12	Gastrocolic ulcer
65737	J12yz00	Unspecified duodenal ulcer NOS
66092	J14yz00	Unspecified gastrojejunal ulcer NOS
67082	J13y000	Unspecified peptic ulcer without mention of complication
67356	J111400	Chronic gastric ulcer with obstruction
67711	J130y00	Acute peptic ulcer unspecified
68661	J130000	Acute peptic ulcer without mention of complication
69663	J131z00	Chronic peptic ulcer NOS
70005	761J111	Suture of ulcer of stomach NEC
70390	J131y00	Chronic peptic ulcer unspecified
70456	J13y100	Unspecified peptic ulcer with haemorrhage
71150	J12y000	Unspecified duodenal ulcer without mention of complication
71403	J110300	Acute gastric ulcer with haemorrhage and perforation
71881	J121300	Chronic duodenal ulcer with haemorrhage and perforation
71897	J111300	Chronic gastric ulcer with haemorrhage and perforation
71904	J12y400	Unspecified duodenal ulcer with obstruction
73338	J11y000	Unspecified gastric ulcer without mention of complication
73417	J120400	Acute duodenal ulcer with obstruction
73697	J11y400	Unspecified gastric ulcer with obstruction
92695	7612111	Balfour excision of gastric ulcer
93436	J12y300	Unspecified duodenal ulcer with haemorrhage and perforation
94104	761Jy00	Other specified operation on gastric ulcer
94397	J11yy00	Unspec gastric ulcer; unspec haemorrhage and/or perforation
96090	J141.00	Chronic gastrojejunal ulcer
96622	J13y300	Unspecified peptic ulcer with haemorrhage and perforation
96628	J140100	Acute gastrojejunal ulcer with haemorrhage
99430	J131000	Chronic peptic ulcer without mention of complication
99670	J13y400	Unspecified peptic ulcer with obstruction
102177	J140200	Acute gastrojejunal ulcer with perforation
106330	J140300	Acute gastrojejunal ulcer with haemorrhage and perforation
109546	J110400	Acute gastric ulcer with obstruction
110244	J141300	Chronic gastrojejunal ulcer with haemorrhage and perforation

ICD-10 Codes for peptic ulcer used in HES and ONS

ICD Code	Description
K25	Gastric ulcer
K25.0	Gastric ulcer : acute with haemorrhage
K25.1	Gastric ulcer : acute with perforation
K25.2	Gastric ulcer : acute with both haemorrhage and perforation
K25.3	Gastric ulcer : acute without haemorrhage or perforation
K25.4	Gastric ulcer : chronic or unspecified with haemorrhage
K25.5	Gastric ulcer : chronic or unspecified with perforation
K25.6	Gastric ulcer : chronic or unspecified with both haemorrhage and perforation
K25.7	Gastric ulcer : chronic without haemorrhage or perforation
K25.9	Gastric ulcer : unspecified as acute or chronic, without haemorrhage or perforation
K26	Duodenal ulcer
K26.0	Duodenal ulcer : acute with haemorrhage
K26.1	Duodenal ulcer : acute with perforation
K26.2	Duodenal ulcer : acute with both haemorrhage and perforation
K26.3	Duodenal ulcer : acute without haemorrhage or perforation
K26.4	Duodenal ulcer : chronic or unspecified with haemorrhage
K26.5	Duodenal ulcer : chronic or unspecified with perforation
K26.6	Duodenal ulcer : chronic or unspecified with both haemorrhage and perforation
K26.7	Duodenal ulcer : chronic without haemorrhage or perforation
K26.9	Duodenal ulcer : unspecified as acute or chronic, without haemorrhage or perforation
K27	Peptic ulcer, site unspecified
K27.0	Peptic ulcer, site unspecified : acute with haemorrhage
K27.1	Peptic ulcer, site unspecified : acute with perforation
K27.2	Peptic ulcer, site unspecified : acute with both haemorrhage and perforation
K27.3	Peptic ulcer, site unspecified : acute without haemorrhage or perforation
K27.4	Peptic ulcer, site unspecified : chronic or unspecified with haemorrhage
K27.5	Peptic ulcer, site unspecified : chronic or unspecified with perforation
K27.6	Peptic ulcer, site unspecified : chronic or unspecified with both haemorrhage and perforation
K27.7	Peptic ulcer, site unspecified : chronic without haemorrhage or perforation
K27.9	Peptic ulcer, site unspecified : unspecified as acute or chronic, without haemorrhage or perforation
K28	Gastrojejunal ulcer
K28.0	Gastrojejunal ulcer : acute with haemorrhage
K28.1	Gastrojejunal ulcer : acute with perforation
K28.2	Gastrojejunal ulcer : acute with both haemorrhage and perforation
K28.3	Gastrojejunal ulcer : acute without haemorrhage or perforation
K28.4	Gastrojejunal ulcer : chronic or unspecified with haemorrhage
K28.5	Gastrojejunal ulcer : chronic or unspecified with perforation
K28.6	Gastrojejunal ulcer : chronic or unspecified with both haemorrhage and perforation
K28.7	Gastrojejunal ulcer : chronic without haemorrhage or perforation
K28.9	Gastrojejunal ulcer : unspecified as acute or chronic, without haemorrhage or perforation

Appendix 2. Potential confounders

Depending on the time to measure the confounder covariates, three sets of covariates measured using the CPRD and HES data were considered in our analysis for adjustment: baseline characteristics, characteristics by the 90th day of stroke, and time-varying characteristics.

Baseline characteristics

Baseline covariates included demographics, comorbidities, lifestyle, healthcare utilisation and co-medications prior to or on the date of index stroke (Figure 1), unless otherwise specified below. These covariates were used to adjust for between-group imbalance in baseline characteristics and 90-day post-stroke loss to follow-up (i.e. baseline selection).

Demographic covariates included age at index stroke, gender and socioeconomic status. Index of Multiple Deprivation (IMD) categorised into quintiles was used as a marker for socioeconomic status. It includes seven domains: income; employment; health and disability; education, skills and training; barriers to housing and services; crime; and living environment. When the patient-level IMD was missing, we replaced it with the practice-level IMD. Stroke subtype, year of stroke diagnosis, and healthcare utilisation (defined as the number of primary care consultation within one year before index stroke) were also included as baseline covariates.

We used previously developed algorithms to identify the following baseline comorbidities, which are considered to be associated with dementia: atrial fibrillation, alcohol use disorders, coronary heart disease, diabetes, heart failure, hyperlipidaemia, hypertension, peripheral artery disease, transient ischemic attack, anxiety, asthma, chronic obstructive pulmonary disease, depression, epilepsy, Parkinson's disease and rheumatoid arthritis. Lifestyle variables, including smoking (current, former, and never smoking) and body mass index (a continuous variable), were defined using the most recent available values before the index stroke.

Co-medications included anticoagulant, antiplatelet, antidiabetic, antihypertensive and non-statin lipid-lowering treatments, which may be associated with the risk of dementia. We defined these medications via product codes recorded within the 365 days prior to the index stroke.

Characteristics by the 90th day following index stroke

These post-stroke covariates included comorbidities, lifestyle and co-medications. We identified these covariates using the same approaches as mentioned above but extended the time window of ascertainment up to the 90th day after stroke. These covariates, as well as baseline demographics, stroke subtype, year of stroke diagnosis and healthcare utilisation, were used to adjust for censoring associated to characteristics at the start of the time-at-risk period.

Time-varying characteristics

Time-varying covariates included comorbidities, lifestyle and co-medications. We identified these using the same approaches as mentioned above in 30-day intervals of follow-up since the index stroke (Figure 1). For covariates not measured in a particular 30-day interval, we carried the last observation forward to impute the value. These covariates, as well as baseline demographics, stroke subtype, year of stroke diagnosis, and healthcare utilisation, were used to adjust for censoring associated with time-varying confounding during the first 90 days (baseline selection) and the subsequent time-at-risk period.

Appendix 3. Quality control criteria

Data item	Unacceptable value
**ALL the records of a patient were excluded for any reason below:**	
First registration date	Empty; invalid date; prior to year of birth; within one year before the first stroke diagnosis date
Current registration date	Invalid date; prior to first registration date; prior to year of birth
Transferred out date	Invalid date; present with no reason; prior to first registration date; prior to current registration date
A transfer-out reason	Present with no date
Registration status	Temporary patients
Age	Over 125 years at the end of follow-up
Year of birth	Absent
Gender	Other than male, female or indeterminate
Death date	Prior to the first registration date; prior to the current registration date
**RELEVANT episode records of a patient were excluded for any reason below:**	
Event date	Invalid; absent; prior to birth year
Weight	<30kg; >300kg
Height	<1.1 metres; >2.3 metres
**The date was CHANGED for any reason below:**	
Change the death date and transfer-out date to the first stroke diagnosis date	Death date prior to the first stroke diagnosis date; transfer-out date prior to the first stroke diagnosis date
Change the death date and transfer-out date to the first date of dementia diagnosis	Death date prior to the first date of dementia diagnosis; transfer-out date prior to the first date of dementia diagnosis

Appendix 4. Details on effect estimation using inverse probability weighted marginal structural models

We estimated the treatment effects via a two-stage process. Firstly, we estimated the four stabilised weights using logistic regression. In the second stage, we used weighted pooled logistic regression models to estimate the observational analogues of intention-to-treat (ITT) and per-protocol (PP) effects.

First stage: weight estimation

Four types of weights were used: baseline treatment weights, baseline selection weights, follow-up weights and treatment persistence weights – to adjust for baseline confounding, baseline selection bias, loss to follow-up, and non-persistence with the initial treatment, respectively.

To reduce the variability of the original weights, we estimated the stabilised version for each weight. We estimated the stabilised weights by fitting two logistic regression models for denominator and numerator, respectively. The denominator model was used for weight estimation and the numerator model was used for stabilisation of the weights. The denominator represented an individual's probability of receiving their treatment (baseline treatment weight), an individual's probability of being followed up in each 30-day interval over the first 90 days (baseline selection weight), an individual's probability of being followed up in each 30-day interval over the time-at-risk period (follow-up weight) and an individual's probability of persistence with initial treatment in each 30-day interval over the time-at-risk period (treatment persistence weight) given their own past treatment history, confounder history, and baseline/by-90th-day covariates (only given baseline covariates for baseline treatment weight). The numerator represented the same respective probability but only conditional on time (cumulative amount of 30-day interval), age, and gender.

Covariates included in denominator and numerator models for each type of weight are summarised in Table A4-1. Age and time were treated as cubic spline variables, BMI and consultation number were treated as continuous variables, and other variables were treated as binary or categorical variables.

Table A4-1. Covariates included in the denominator and numerator models for the weight calculation

Type of weight	Denominator model	Numerator model
Baseline treatment weight	All baseline covariates	Age and gender
Baseline selection weight	All baseline and time-varying covariates	Age, gender, and time
Follow-up weight	All by-90th-day and time-varying covariates	Age, gender, and time
Treatment persistence weight	All by-90th-day and time-varying covariates	Age, gender, and time

The models of weights were fitted separately by treatment group, except for baseline treatment weight.

Second stage: ITT and PP effect estimation

In this stage, we used weighted pooled logistic regression models to estimate two types of observational effects of statin use: the effect of initiation vs no initiation (observational analogue of ITT analysis) and the effect of sustained use vs no use (observational analogue of PP analysis). In the ITT analysis, the primary model was conducted with full adjustment for baseline characteristics, baseline selection bias and loss to follow-up using the product of baseline treatment weights, baseline selection weights, and follow-up weights. In the PP analysis, the primary model additionally accounted for artificial censoring due to deviation from initial treatment status during time-at-risk period using the product of all the four types of weights. The final weights used in the outcome models were truncated at 1st and 99th percentiles to minimise the impact of extreme weights and improve precision. We used a robust variance estimator to estimate the 95% confidence intervals, given the potential correlation introduced by weighting. A P-value of less than 0.05 was considered of statistical significance.

Mathematical details

Details about the models used in the two stages are listed as below.

Baseline treatment weight

Inverse probability weighting derived from propensity scores was used to adjust for the imbalance in the baseline characteristics between the two treatment groups (i.e., propensity score weighting).

Let A denote treatment received (A = 1 denoting statin initiation; A = 0 denoting no statin initiation), and let X denote a vector of the observed baseline covariates. The propensity score

is defined as PS = P(A = 1|X), representing the probability of a subject initiating statin treatment conditional on all the observed baseline covariates.

In the denominator model, the propensity score, denoted as PS_d, was estimated using a logistic regression model in which the treatment indicator (A = 1 or 0) was regressed on the all the observed baseline covariates listed above. In the numerator model, we calculated the probability of a subject initiating statin treatment conditional only on baseline age (as restricted cubic smoothing splines with four knots) and gender, denoted as PS_n, using the same modelling approach to stabilise the weights.

The stabilised inverse probability of treatment weights was calculated as:

$$SW = \frac{A \times PS_n}{PS_d} + \frac{(1-A)(1-PS_n)}{1-PS_d} \quad (1)$$

The baseline treatment weight was the same for each 30-day interval of a subject through the time-at-risk period.

Baseline selection weight

Potential selection bias due to death or transfer-out within the first 90 days following index stroke before the time-at-risk period was accounted for via the use of inverse probability of censoring weights.

Let C(t) be a dichotomous variable taking the value 1 if a subject is censored in time interval t and 0 otherwise (for this type of weight calculation, t=1, 2, 3). $\bar{C}(t)$ denotes censoring history (that is, the vector of values of C(k) from k = 1 to k = t − 1). $\bar{L}(k-1)$ denotes time-varying covariate history (that is, the matrix of values of L(k-1) from k = 1 to k = t).To adjust for baseline selection bias in the marginal structural model, we derived the probability of remaining uncensored up to time t, and calculated the stabilised version of the baseline selection weight as:

$$CW_S(t) = \prod_{k=1}^{t} \frac{P[C(k)=0 \mid A=a, \bar{C}(k-1)=\bar{0}, V]}{P[C(k)=0 \mid A=a, \bar{C}(k-1)=\bar{0}, \bar{L}(k-1), V]} \quad (2)$$

In this equation (2), A denotes initial treatment, which is a dichotomous variable, taking the value 1 if a subject received a statin within the first 90 days of stroke and 0 otherwise (i.e., the weight was estimated separately by treatment group). We treated it as a baseline variable, instead of a time-varying variable over this period, because the research question of interest is “starting a stain within the first 3 months of stroke” vs “never starting a statin in the first 3 months”. V denotes a subset of the baseline covariates and time, which included age (as

restricted cubic smoothing splines with four knots), gender, and 30-day interval (as indicator variables) in this model. L(k-1) denotes the values of all time-varying covariates at k=t. As an exception, L(0) was defined to be the complement baseline covariate set of V.

Follow-up weight

Potential bias due to death or transfer-out during the time-at-risk period was accounted for via the use of inverse probability of follow-up weights. Using similar approach mentioned above, we derived the probability of remaining uncensored up to time interval t, and calculated the stabilised version of the follow-up weight as:

$$CW_F(t) = \prod_{k=4}^{t} \frac{P[C(k)\ |A=a, \bar{C}(k-1)=\bar{0}, V]}{P[C(k)|A=a, \bar{C}(k-1)=\bar{0}, \bar{L}(k-1), V]} \quad (3)$$

The differences between (2) and (3) were as below. The weight was calculated from the fourth 30-day interval, i.e., t=4, 5,…, T). V included age (as restricted cubic smoothing splines with four knots), gender, and time (as restricted cubic smoothing splines with four knots). Specifically, L(3) denotes the values of all time-varying covariates measured up to the third 30-day interval following stroke. The weight was estimated separately by treatment group.

Treatment persistence weight

In the observational analogue of PP analysis, we artificially censored patients discontinuing their initial treatments. To account for the treatment non-persistence during the time-at-risk period, we derived the probability of remaining on initial treatment up to time interval t, and calculated the stabilised version of the treatment persistence weight as:

$$CW_A(t) = \prod_{k=4}^{t} \frac{P[C_A(k)\ |A=a, \bar{C}_A(k-1)=\bar{0}, V]}{P[C_A(k)|A=a, \bar{C}_A(k-1)=\bar{0}, \bar{L}(k-1), V]} \quad (4)$$

In the equation (4), $C_A(t)$ was defined as a dichotomous variable taking the value 1 if a subject changed the initial treatment in time interval t and 0 otherwise (t=4, 5,…, t). $\bar{C}_A(t)$ denotes persistence history (that is, the vector of values of $C_A(k)$ from k = 4 to k = t − 1). A denotes initial treatment, which is a dichotomous variable, taking the value 1 if a subject received a statin within the first 90 days of stroke and 0 otherwise (i.e., a=0, 1). All the patients were considered to persist with the initial treatment over the time-at-risk period until they discontinued statins (statin initiation group) or start on statins (statin non-initiation group) (as defined in the Follow-up section of the main paper) and thus were artificially censored. $\bar{L}(k-1)$ denotes history of time-varying confounders for a subject (that is, the matrix of values of L(k-1) from k = 4 to k = t). In this model, V included age (as restricted

cubic smoothing splines with four knots), gender, and time (as restricted cubic smoothing splines with four knots). L(k-1) denotes the values of all time-varying covariates at k=t. As an exception, L(3) was defined to be the complement covariate set of V measured up to the third 30-day interval following stroke. The weight was estimated separately by treatment group.

Estimating the outcome model

Using the weights calculated as above, we estimated the hazard ratio for the effects of interest using a weighted pooled logistic regression model, which approximates a time-dependent Cox model [1].

We defined the outcome event Y at each time point over the time-at-risk period, $t = 4, 5, \ldots, T$, to be $Y(t)$. $Y(t) = 0$ persisted for all 30-day intervals until the interval in which the outcome event occurs, at which point $Y(t) = 1$. The risk of the outcome was evaluated with respect to the initial treatment, i.e., $Y(t)$ depends on A, which was assumed unchanged over the whole time-at-risk period in the ITT analysis. For this analysis, we fitted three different outcome models using the pooled logistic regression as:

Model 1: $logit[P(Y(t) = 1|\bar{Y}(t-1) = \bar{0}, A, V)] = \beta_0 + \beta A + \boldsymbol{\gamma} V$ (5)

Model 2: $logit[P(Y(t) = 1|\bar{Y}(t-1) = \bar{0}, \bar{C}_S(t) = \bar{0}, A, V)] = \beta_0 + \beta A + \boldsymbol{\gamma} V$ (6)

Model 3: $logit[P(Y(t) = 1|\bar{Y}(t-1) = \bar{0}, \bar{C}_S(t) = \bar{0}, \bar{C}_F(t) = \bar{0}, A, V)] = \beta_0 + \beta A + \boldsymbol{\gamma} V$ (7)

In model 1, we adjusted for the between-group imbalance in baseline characteristics. The inverse probability of weights used in the model was SW.

In model 2, we additionally adjusted for potential selection bias within the first 90 days of stroke. The inverse probability of weights used in the model was $SW \times CW_S(t)$.

In model 3, we additionally adjusted for loss to follow-up during the time-at-risk period after the first 90 days of stroke. The inverse probability of weights used in the model was $SW \times CW_S(t) \times CW_F(t)$.

For the PP analysis in which patients deviated from their initial treatment were artificially censored, we fitted model 1 and 2 using the same approach above. In model 3, we additionally adjusted for treatment non-persistence during the time-at-risk period after the first 90 days of stroke as:

$logit[P(Y(t) = 1|\bar{Y}(t-1) = \bar{0}, \bar{C}_S(t) = \bar{0}, \bar{C}_F(t) = \bar{0}, \bar{C}_A(t) = \bar{0}, A, V)] = \beta_0 + \beta A + \boldsymbol{\gamma} V$ (8)

The inverse probability of weights used in the model was $SW \times CW_S(t) \times CW_F(t) \times CW_A(t)$.

In (5) to (8), β was the parameter of interest. Because the stabilisation of the weight means that treatment initiation is balanced conditional on all variables V (age, gender and time), these variables included in the numerator model of the stabilised weight were included in the outcome model. The marginal structural models also accounted for the dependence between observations from the same subject which were introduced by the weighting process, and therefore the variance was estimated using a robust variance estimator [2].

1. Dagostino RB, Lee ML, Belanger AJ, Cupples LA, Anderson K, Kannel WB. Relation of pooled logistic-regression to time-dependent Cox regression analysis: the Framingham Heart Study. *Stat Med*. 1990;9(12):1501-15.
2. Hernán M, Robins J. Causal inference: what if. Boca Raton, USA: Chapman & Hall/CRC; 2020.

Appendix 5. Baseline characteristics

Table A5-1. Baseline characteristics by completeness of baseline data

	Total (N=38,034)	Complete (N=33,190)	Incomplete (N=4844)	SMD
**Demographics**				
Age at stroke, median (IQR)	74 (62-83)	74 (62-83)	74 (60-85)	0.004
Female	19,609 (51.6)	17,231 (51.9)	2378 (49.1)	0.06
IMD Group 1 (least deprived)	8236 (21.7)	7174 (21.6)	1062 (21.9)	0.07
Group 2	7228 (19.0)	6263 (18.9)	965 (19.9)	
Group 3	8337 (21.9)	7238 (21.8)	1099 (22.7)	
Group 4	7637 (20.1)	6644 (20.0)	993 (20.5)	
Group 5	6596 (17.3)	5871 (17.7)	725 (15.0)	
**Stroke subtype**				
Ischaemic[#]	34,395 (90.4)	30,113 (90.7)	4282 (88.4)	0.08
Haemorrhagic	3639 (9.6)	3077 (9.3)	562 (11.6)	
**Cardiovascular factors***				
AF	6810 (17.9)	6056 (18.2)	754 (15.6)	0.07
Alcohol use disorders	1884 (5.0)	1651 (5.0)	233 (4.8)	0.01
CHD	4108 (10.8)	3777 (11.4)	331 (6.8)	0.16
Diabetes	3242 (8.5)	3069 (9.2)	173 (3.6)	0.23
Heart failure	2288 (6.0)	2023 (6.1)	265 (5.5)	0.03
Hyperlipidaemia	5219 (13.7)	4945 (14.9)	274 (5.7)	0.31
Hypertension	18,571 (48.8)	16,871 (50.8)	1700 (35.1)	0.32
PAD	1202 (3.2)	1099 (3.3)	103 (2.1)	0.07
TIA	3200 (8.4)	2851 (8.6)	349 (7.2)	0.05
Smoking Current	7983 (21.2)	6807 (20.5)	1176 (26.1)	0.23
Former	11,044 (29.3)	10,104 (30.4)	940 (20.9)	
Never	18,663 (49.5)	16,279 (49.1)	2384 (53.0)	
BMI, median (IQR)	26.1 (23.1-29.4)	26.1 (23.1-29.4)	26.8 (23.6-31.0)	0.20
**Other comorbidities***				
Anxiety	7022 (18.5)	6420 (19.3)	602 (12.4)	0.19
Rheumatoid arthritis	2397 (6.3)	2178 (6.6)	219 (4.5)	0.09
Asthma	4608 (12.1)	4328 (13.0)	280 (5.8)	0.25
COPD	3169 (8.3)	2976 (9.0)	193 (4.0)	0.20
Depression	9571 (25.2)	8679 (26.1)	892 (18.4)	0.19
Epilepsy	1140 (3.0)	1000 (3.0)	140 (2.9)	0.01
Hearing loss	7942 (20.9)	7205 (21.7)	737 (15.2)	0.17
Parkinson's disease	476 (1.3)	406 (1.2)	70 (1.4)	0.02
**Healthcare utilisation***				
Number of consultations, median (IQR)	27 (15-44)	28 (16-45)	19 (6-36)	0.38
**Co-medications***				
Anticoagulant drugs	2143 (5.6)	1977 (6.0)	166 (3.4)	0.12
Antiplatelet drugs	8517 (22.4)	7644 (23.0)	873 (18.0)	0.12
Antidiabetic drugs	2006 (5.3)	1948 (5.9)	58 (1.2)	0.26
Antihypertensive drugs	18,969 (49.9)	17,272 (52.0)	1697 (35.0)	0.35
Other lipid-lowering drugs	730 (1.9)	712 (2.1)	18 (0.4)	0.16

*Cardiovascular factors and other comorbidities were defined up until the index stroke; health care utilisation and co-medications were defined within one years before the index stroke.
#A total of 16,352 (43.0%) patients had an unspecified stroke subtype: 14,352 (43.2%) and 2000 (41.3%) patients with complete and incomplete baseline data, respectively.
Abbreviations: AF, atrial fibrillation; BMI, body mass index; CHD, coronary heart disease; COPD, chronic obstructive pulmonary disease; IMD, Index of Multiple Deprivation; PAD, peripheral artery disease; SMD, standardised mean difference; TIA, transient ischaemic attack.

Table A5-2. Baseline characteristics by survival status within the first 90 days of stroke

	Survival (N=29,601)	Death (N=2907)	Transfer-out (N=682)
**Demographics**			
Age at stroke, median (IQR)	73 (62-82)	84 (74-90)	82 (69-88)
Female	15,066 (50.9)	1758 (60.5)	407 (59.7)
IMD Group 1 (least deprived)	6404 (21.6)	614 (21.1)	156 (22.9)
Group 2	5576 (18.8)	560 (19.3)	127 (18.6)
Group 3	6422 (21.7)	647 (22.3)	169 (24.8)
Group 4	5943 (20.1)	574 (19.7)	127 (18.6)
Group 5	5256 (17.8)	512 (17.6)	103 (15.1)
**Stroke subtype**			
Ischaemic[#]	27,188 (91.1)	2317 (79.7)	608 (89.1)
Haemorrhagic	2413 (8.2)	590 (20.3)	74 (10.9)
**Cardiovascular factors***			
AF	4950 (16.7)	940 (32.3)	166 (24.3)
Alcohol use disorders	1482 (5.0)	138 (4.7)	31 (4.5)
CHD	3077 (10.4)	593 (20.4)	107 (15.7)
Diabetes	2605 (8.8)	377 (13.0)	87 (12.8)
Heart failure	1484 (5.0)	480 (16.5)	59 (8.7)
Hyperlipidaemia	4457 (15.1)	364 (12.5)	124 (18.2)
Hypertension	14,695 (49.6)	1767 (60.8)	409 (60.0)
PAD	871 (2.9)	198 (6.8)	30 (4.4)
TIA	2579 (8.7)	187 (6.4)	85 (12.5)
Smoking Current	6276 (21.2)	416 (14.3)	115 (16.8)
Former	8643 (29.2)	1200 (41.3)	261 (38.3)
Never	14,682 (49.6)	1291 (44.4)	306 (44.9)
BMI, median (IQR)	26.2 (23.2-29.6)	24.6 (21.7-28.0)	25.6 (22.7-29.3)
**Other comorbidities***			
Anxiety	5726 (19.3)	558 (19.2)	136 (19.9)
Rheumatoid arthritis	1860 (6.3)	259 (8.9)	59 (8.7)
Asthma	3896 (13.2)	360 (12.4)	72 (10.6)
COPD	2537 (8.6)	375 (12.9)	64 (9.4)
Depression	7771 (26.3)	714 (24.6)	194 (28.4)
Epilepsy	883 (3.0)	90 (3.1)	27 (4.0)
Hearing loss	6192 (20.9)	856 (29.4)	157 (23.0)
Parkinson's disease	340 (1.1)	59 (2.0)	7 (1.0)
**Healthcare utilisation***			
Number of consultations, median (IQR)	28 (15-43)	39 (25-58)	28 (15-46)
**Co-medications***			
Anticoagulant drugs	1617 (5.5)	313 (10.8)	47 (6.9)
Antiplatelet drugs	6519 (22.0)	965 (33.2)	160 (23.5)
Antidiabetic drugs	1658 (5.6)	237 (8.2)	53 (7.8)
Antihypertensive drugs	14,929 (50.4)	1947 (67.0)	396 (58.1)
Other lipid-lowering drugs	655 (2.2)	50 (1.7)	7 (1.0)

*Cardiovascular factors and other comorbidities were defined up until the index stroke; health care utilisation and co-medications were defined within one years before the index stroke.
#A total of 14,352 (43.2%) patients had an unspecified stroke subtype: 12,873 (43.5%), 1253 (43.1%) and 226 (33.1%) for survival, death and transfer-out, respectively.
Abbreviations: AF, atrial fibrillation; BMI, body mass index; CHD, coronary heart disease; COPD, chronic obstructive pulmonary disease; IMD, Index of Multiple Deprivation; PAD, peripheral artery disease; SMD, standard mean difference; SD, standard deviation; TIA, transient ischaemic attack.

Appendix 6. Incidence of post-stroke dementia

Figure A6-1. Kaplan-Meier plots for dementia by statin initiation

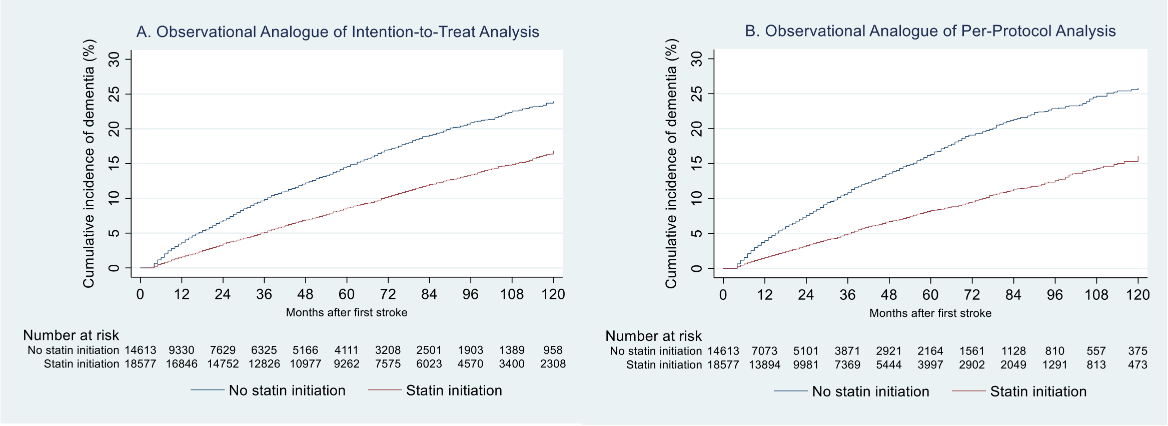

Table A6-1. Age and gender specific incidence rates of dementia in comparison with CFAS II

	Eligible stroke patients in the CPRD (N=29,601) per 1000 person years	General population in the CFAS II (N=5288) per 1000 person years [1]
Male (age years)		
65-69	9.4	5.0
70-74	19.2	8.7
75-79	29.0	16.7
80-84	45.6	24.8
≥85	60.6	38.0
Female (age years)		
65-69	10.1	4.6
70-74	17.7	6.4
75-79	32.7	16.1
80-84	45.9	39.6
≥85	66.6	55.3

Abbreviations: CFAS, Cognitive Function and Ageing Studies; CPRD, Clinical Practice Research Datalink.

The incidence rates of dementia in stroke patients observed in our study were 1.2 to 2.8 times as high as that in the general population from the Cognitive Function and Ageing Studies II [1], approximately in line with the risk ratios summarised in the meta-analysis, which ranged from 1.42 (95% CI 1.20-1.67) to 3.28 (1.92-5.62) [2].

Reference:

1. Matthews FE, Stephan BC, Robinson L, Jagger C, Barnes LE, Arthur A, et al. A two decade dementia incidence comparison from the Cognitive Function and Ageing Studies I and II. *Nat Commun*. 2016;7:11398.
2. Kuzma E, Lourida I, Moore SF, Levine DA, Ukoumunne OC, Llewellyn DJ. Stroke and dementia risk: a systematic review and meta-analysis. *Alzheimers Dement*. 2018;14(11):1416-26.

Appendix 7. Distributions of weights in marginal structural models

Table A7-1. Distributions of weights for dementia

		ITT analysis		PP analysis	
		Mean (SD)	Median (min, max)	Mean (SD)	Median (min, max)
Treatment weight		1.00 (0.40)	0.91 (0.32, 25.55)	0.99 (0.39)	0.91 (0.32, 25.65)
Baseline selection		1.00 (0.07)	1.00 (0.57, 4.04)	1.00 (0.08)	1.00 (0.57, 4.06)
Complete follow-up		1.02 (0.62)	0.99 (0.27, 263.53)	1.01 (1.20)	0.99 (0.25, 518.16)
Treatment persistence		NA	NA	1.01 (0.64)	0.96 (0.14, 155.10)
Model 1 [a]	Original	1.00 (0.40)	0.91 (0.32, 25.55)	0.99 (0.39)	0.91 (0.32, 25.65)
	Truncated*	1.00 (0.32)	0.91 (0.46, 2.53)	0.98 (0.31)	0.91 (0.45, 2.48)
Model 2 [b]	Original	1.00 (0.39)	0.91 (0.32, 26.66)	0.99 (0.39)	0.90 (0.33, 26.77)
	Truncated*	0.99 (0.31)	0.91 (0.48, 2.54)	0.98 (0.31)	0.90 (0.47, 2.49)
Model 3 [c]	Original	1.02 (0.81)	0.91 (0.19, 280.84)	1.05 (1.64)	0.87 (0.07, 480.14)
	Truncated*	1.01 (0.39)	0.91 (0.45, 2.94)	1.00 (0.58)	0.87 (0.27, 3.89)

a. Model 1: adjusted for baseline characteristics and months of follow-up.
b. Model 2: adjusted for baseline selection plus Model 1.
c. Model 3: adjusted for loss to follow-up plus Model 2. For PP analysis, artificial censoring due to deviation from initial treatment during time-at-risk period was additionally accounted for.
*Extreme values were truncated to 1st and 99th percentiles.
Abbreviations: ITT, intention-to-treat; max, maximum weight; min, minimum weight; NA, not applicable; PP, per-protocol; SD, standard deviation.

Table A7-2. Distributions of weights for control outcomes

		CHD		Fracture		Peptic ulcer	
		Mean (SD)	Median (min, max)	Mean (SD)	Median (min, max)	Mean (SD)	Median (min, max)
**ITT analysis**							
Treatment weight		1.01 (0.38)	0.92 (0.32, 8.99)	1.01 (0.38)	0.91 (0.33, 10.85)	1.00 (0.40)	0.91 (0.33, 26.16)
Baseline selection		1.00 (0.06)	1.00 (0.62, 4.39)	1.00 (0.07)	1.00 (0.56, 3.34)	1.00 (0.07)	1.00 (0.58, 3.80)
Complete follow-up		1.03 (2.74)	0.99 (0.25, 954.85)	1.02 (0.85)	0.99 (0.24, 176.18)	1.06 (6.00)	0.99 (0.20, 2025.58)
Model 1 [a]	Original	1.01 (0.38)	0.92 (0.32, 8.99)	1.01 (0.38)	0.91 (0.33, 10.85)	1.00 (0.40)	0.91 (0.33, 26.16)
	Truncated*	1.00 (0.32)	0.92 (0.45, 2.57)	1.00 (0.32)	0.91 (0.46, 2.48)	1.00 (0.32)	0.91 (0.46, 2.55)
Model 2 [b]	Original	1.00 (0.37)	0.91 (0.30, 8.99)	1.00 (0.37)	0.91 (0.31, 11.79)	1.00 (0.40)	0.91 (0.32, 27.30)
	Truncated*	0.99 (0.31)	0.91 (0.47, 2.59)	0.99 (0.31)	0.91 (0.48, 2.49)	0.99 (0.32)	0.91 (0.48, 2.56)
Model 3 [c]	Original	1.04 (3.91)	0.91 (0.17, 1365.83)	1.04 (1.37)	0.91 (0.16, 293.17)	1.08 (5.87)	0.91 (0.14, 1905.39)
	Truncated*	1.01 (0.37)	0.91 (0.45, 2.91)	1.01 (0.39)	0.91 (0.44, 2.94)	1.01 (0.41)	0.91 (0.44, 3.09)
**PP analysis**							
Treatment weight		1.00 (0.38)	0.91 (0.32, 8.96)	0.99 (0.38)	0.91 (0.33, 10.91)	0.99 (0.40)	0.91 (0.33, 26.26)
Baseline selection		1.00 (0.07)	1.00 (0.61, 4.44)	1.00 (0.07)	1.00 (0.55, 3.34)	1.00 (0.08)	1.00 (0.58, 3.82)
Complete follow-up		1.01 (0.75)	0.99 (0.26, 299.86)	1.01 (0.65)	0.99 (0.24, 154.28)	1.27 (39.9)	0.99 (0.20, 10807.8)
Treatment persistence		1.00 (0.44)	0.96 (0.15, 56.44)	1.01 (0.62)	0.96 (0.16, 117.18)	1.01 (0.74)	0.96 (0.14, 216.18)
Model 1 [a]	Original	1.00 (0.38)	0.91 (0.32, 8.96)	0.99 (0.38)	0.91 (0.33, 10.91)	0.99 (0.40)	0.91 (0.33, 26.26)
	Truncated*	0.99 (0.31)	0.91 (0.44, 2.55)	0.99 (0.31)	0.91 (0.46, 2.45)	0.98 (0.31)	0.91 (0.46, 2.49)
Model 2 [b]	Original	0.99 (0.38)	0.91 (0.31, 8.96)	0.99 (0.38)	0.91 (0.31, 11.86)	0.99 (0.39)	0.90 (0.32, 27.41)
	Truncated*	0.98 (0.31)	0.91 (0.46, 2.57)	0.98 (0.30)	0.91 (0.47, 2.46)	0.98 (0.31)	0.90 (0.47, 2.48)
Model 3 [c]	Original	1.04 (1.38)	0.88 (0.08, 349.54)	1.05 (1.64)	0.87 (0.08, 289.47)	1.51 (74.5)	0.87 (0.06, 20511.1)
	Truncated*	1.00 (0.56)	0.88 (0.28, 3.70)	1.00 (0.57)	0.87 (0.27, 3.79)	1.01 (0.61)	0.87 (0.26, 4.20)

a. Model 1: adjusted for baseline characteristics and months of follow-up.
b. Model 2: adjusted for baseline selection plus Model 1.
c. Model 3: adjusted for loss to follow-up plus Model 2. For PP analysis, artificial censoring due to deviation from initial treatment during time-at-risk period was additionally accounted for.
*Extreme values were truncated to 1st and 99th percentiles.
Abbreviations: CHD, coronary heart disease; ITT, intention-to-treat; max, maximum weight; min, minimum weight; PP, per-protocol; PSD, post-stroke dementia; SD, standard deviation.

Appendix 8. Sensitivity analysis

Table A8-1. Sensitivity analysis with different criteria for weight truncation

Outcome*	Truncation at 0.5th and 99.5th percentiles aHR (95% CI)	Truncation at 0.1 and 10 aHR (95% CI)	Original weight aHR (95% CI)
**ITT analysis**			
PSD	0.70 (0.64-0.75)	0.71 (0.65-0.77)	0.72 (0.65-0.79)
CHD	0.87 (0.79-0.96)	0.88 (0.79-0.97)	0.91 (0.80-1.04)
Fracture	0.88 (0.80-0.97)	0.88 (0.79-0.97)	0.88 (0.78-0.98)
Peptic ulcers	1.04 (0.83-1.30)	1.04 (0.83-1.32)	1.10 (0.85-1.41)
**PP analysis**			
PSD	0.55 (0.50-0.62)	0.56 (0.50-0.63)	0.66 (0.47-0.92)
CHD	0.70 (0.61-0.80)	0.68 (0.59-0.78)	0.64 (0.51-0.80)
Fracture	0.85 (0.74-0.98)	0.85 (0.73-0.99)	0.76 (0.58-1.01)
Peptic ulcers	1.10 (0.76-1.59)	1.05 (0.70-1.58)	1.13 (0.72-1.77)

*PSD: Of 33,190 eligible patients (contributing to weight calculation), 29,601 patients who survived at month 3 were included in the outcome models.

CHD: Of 28,946 eligible patients (contributing to weight calculation), 26,098 patients who survived at month 3 were included in the outcome models.

Fracture: Of 22,850 eligible patients (contributing to weight calculation), 20,545 patients who survived at month 3 were included in the outcome models.

Peptic ulcer: Of 31,042 eligible patients (contributing to weight calculation), 27,647 patients who survived at month 3 were included in the outcome models.

All the outcome models adjusted for baseline characteristics, baseline selection, loss to follow-up and months of follow-up. For PP analysis, artificial censoring due to deviation from initial treatment during time-at-risk period was additionally accounted for.

Abbreviations: aHR, adjusted hazard ratio; CHD, coronary heart disease; CI, confidence interval; IMD, Index of Multiple Deprivation; ITT, intention-to-treat; PP, per-protocol.

Table A8-2. Sensitivity analysis with extreme-case analysis for missing BMI and smoking

		ITT analysis	PP analysis#
Imputation with 5th percentile BMI and never smoking			
Crude HR (95% CI)*		0.58 (0.54-0.62)	0.48 (0.44-0.52)
Adjusted HR (95% CI)**	Model 1 [a]	0.72 (0.67-0.77)	0.62 (0.56-0.68)
	Model 2 [b]	0.71 (0.66-0.76)	0.61 (0.55-0.66)
	Model 3 [c]	0.70 (0.65-0.75)	0.56 (0.51-0.62)
Imputation with 95th percentile BMI and never smoking			
Crude HR (95% CI)*		0.58 (0.54-0.62)	0.48 (0.44-0.52)
Adjusted HR (95% CI)**	Model 1 [a]	0.72 (0.67-0.77)	0.61 (0.56-0.67)
	Model 2 [b]	0.71 (0.66-0.76)	0.60 (0.55-0.66)
	Model 3 [c]	0.69 (0.65-0.75)	0.55 (0.50-0.61)

*Of 38,034 eligible patients, 33,700 patients who survived at month 3 were included in the outcome models.

**38,034 eligible patients contributed to weight calculation, of whom 33,700 patients who survived at month 3 were included in the outcome models.

#Deviation from initial treatment during follow-up was artificially censored in all the models.

a. Model 1: adjusted for baseline characteristics and months of follow-up.

b. Model 2: adjusted for baseline selection plus Model 1.

c. Model 3: adjusted for loss to follow-up plus Model 2. For PP analysis, artificial censoring due to deviation from initial treatment during time-at-risk period was additionally accounted for.

Abbreviations: BMI, body mass index; CI, confidence interval; HR, hazard ratio; ITT, intention-to-treat; PP, per-protocol.

Table A8-3. Sensitivity analysis separating unspecified stroke and ischaemic stroke

		**ITT analysis**	**PP analysis#**
Crude HR (95% CI)*		0.58 (0.54-0.62)	0.47 (0.43-0.52)
Adjusted HR (95% CI)**	Model 1 [a]	0.71 (0.66-0.76)	0.60 (0.55-0.66)
	Model 2 [b]	0.70 (0.65-0.75)	0.59 (0.54-0.65)
	Model 3 [c]	0.69 (0.64-0.74)	0.55 (0.49-0.61)

*Of 33,190 eligible patients, 29,601 patients who survived at month 3 were included in the outcome models.
**33,190 eligible patients contributed to weight calculation, of whom 29,601 patients who survived at month 3 were included in the outcome models.
#Deviation from initial treatment during follow-up was artificially censored in all the models.
a. Model 1: adjusted for baseline characteristics and months of follow-up.
b. Model 2: adjusted for baseline selection plus Model 1.
c. Model 3: adjusted for loss to follow-up plus Model 2. For PP analysis, artificial censoring due to deviation from initial treatment during time-at-risk period was additionally accounted for.
Abbreviations: CI, confidence interval; HR, hazard ratio; ITT, intention-to-treat; PP, per-protocol.

Table A8-4. Sensitivity analysis restricted to patients with linkage to Hospital Episode Statistics

		**ITT analysis**	**PP analysis#**
Crude HR (95% CI)*		0.58 (0.54-0.63)	0.46 (0.41-0.51)
Adjusted HR (95% CI)**	Model 1 [a]	0.72 (0.66-0.79)	0.59 (0.52-0.66)
	Model 2 [b]	0.71 (0.65-0.77)	0.58 (0.51-0.65)
	Model 3 [c]	0.70 (0.64-0.76)	0.53 (0.47-0.60)

*Of 18,462 eligible patients, 16,274 patients who survived at month 3 were included in the outcome models.
**18,462 eligible patients contributed to weight calculation, of whom 16,274 patients who survived at month 3 were included in the outcome models.
Deviation from initial treatment during follow-up was artificially censored in all the models.
a. Model 1: adjusted for baseline characteristics and months of follow-up.
b. Model 2: adjusted for baseline selection plus Model 1.
c. Model 3: adjusted for loss to follow-up plus Model 2. For PP analysis, artificial censoring due to deviation from initial treatment during time-at-risk period was additionally accounted for.
Abbreviations: CI, confidence interval; HR, hazard ratio; ITT, intention-to-treat; PP, per-protocol.

Table A8-5. Sensitivity analysis excluding dementia occurring within the first 6 months after stroke

		**ITT analysis**	**PP analysis#**
Crude HR (95% CI)*		0.61 (0.57-0.66)	0.49 (0.45-0.56)
Adjusted HR (95% CI)**	Model 1 [a]	0.75 (0.69-0.81)	0.64 (0.58-0.71)
	Model 2 [b]	0.73 (0.68-0.79)	0.63 (0.57-0.69)
	Model 3 [c]	0.72 (0.67-0.78)	0.57 (0.51-0.64)

*Of 32,906 eligible patients, 29,317 patients who survived at month 3 were included in the outcome models.
**32,906 eligible patients contributed to weight calculation. Of whom, 29,317 patients who survived at month 3 were included in the outcome model.
#Deviation from initial treatment during follow-up was artificially censored in all the models.
a. Model 1: adjusted for baseline characteristics and months of follow-up.
b. Model 2: adjusted for baseline selection plus Model 1.
c. Model 3: adjusted for loss to follow-up plus Model 2. For PP analysis, artificial censoring due to deviation from initial treatment during time-at-risk period was additionally accounted for.
Abbreviations: CI, confidence interval; HR, hazard ratio; ITT, intention-to-treat; PP, per-protocol.

Table A8-6. Sensitivity analysis excluding dementia occurring within the first 12 months after stroke

		ITT analysis	PP analysis#
Crude HR (95% CI)*		0.66 (0.61-0.72)	0.52 (0.47-0.58)
Adjusted HR (95% CI)**	Model 1 [a]	0.78 (0.72-0.85)	0.68 (0.61-0.76)
	Model 2 [b]	0.77 (0.71-0.84)	0.66 (0.59-0.74)
	Model 3 [c]	0.75 (0.69-0.82)	0.59 (0.52-0.68)

*Of 32,520 eligible patients, 28,931 patients who survived at month 3 were included in the outcome models.
**32,520 eligible patients contributed to weight calculation. Of whom, 28,931 patients who survived at month 3 were included in the outcome model.
#Deviation from initial treatment during follow-up was artificially censored in all the models.
a. Model 1: adjusted for baseline characteristics and months of follow-up.
b. Model 2: adjusted for baseline selection plus Model 1.
c. Model 3: adjusted for loss to follow-up plus Model 2. For PP analysis, artificial censoring due to deviation from initial treatment during time-at-risk period was additionally accounted for.
Abbreviations: CI, confidence interval; HR, hazard ratio; ITT, intention-to-treat; PP, per-protocol.

Table A8-7. Sensitivity analysis changing the time frame for defining initial treatment

Time window		ITT analysis		PP analysis#	
		1 month*	6 months**	1 month*	6 months**
cHR (95% CI)*		0.58 (0.54-0.62)	0.57 (0.53-0.62)	0.40 (0.36-0.44)	0.50 (0.45-0.54)
aHR (95% CI)**	Model 1 [a]	0.72 (0.67-0.78)	0.72 (0.66-0.78)	0.54 (0.49-0.60)	0.65 (0.59-0.72)
	Model 2 [b]	0.72 (0.67-0.77)	0.71 (0.66-0.77)	0.53 (0.48-0.59)	0.64 (0.58-0.71)
	Model 3 [c]	0.71 (0.66-0.77)	0.72 (0.66-0.78)	0.50 (0.45-0.56)	0.62 (0.55-0.69)

*33,449 eligible patients contributed to weight calculation. Of them, 31,117 patients who survived at month 1 were included in the outcome models.
**32,906 eligible patients contributed to weight calculation. Of them, 26,205 patients who survived at month 6 were included in the outcome models.
#Deviation from initial treatment during follow-up was artificially censored in all the models.
a. Model 1: adjusted for baseline characteristics and months of follow-up.
b. Model 2: adjusted for baseline selection plus Model 1.
c. Model 3: adjusted for loss to follow-up plus Model 2. For PP analysis, artificial censoring due to deviation from initial treatment during time-at-risk period was additionally accounted for.
Abbreviations: aHR, adjusted hazard ratio; cHR, crude hazard ratio; CI, confidence interval; ITT, intention-to-treat; PP, per-protocol.

Table A8-8. Sensitivity analysis varying the grace period for defining statin discontinuation

Grace period		One month	Five months
Crude HR (95% CI)*		0.52 (0.47-0.57)	0.48 (0.44-0.52)
Adjusted HR (95% CI)**	Model 1 [a]	0.59 (0.53-0.66)	0.64 (0.58-0.70)
	Model 2 [b]	0.58 (0.52-0.65)	0.62 (0.57-0.68)
	Model 3 [c]	0.51 (0.44-0.58)	0.58 (0.53-0.64)

*Of 33,190 eligible patients, 29,601 patients who survived at month 3 were included in the outcome models.
**33,190 eligible patients contributed to weight calculation, 29,601 patients who survived at month 3 were included in the outcome models.
#Deviation from initial treatment during follow-up was artificially censored in all the models.
a. Model 1: adjusted for baseline characteristics and months of follow-up.
b. Model 2: adjusted for baseline selection plus Model 1.
c. Model 3: adjusted for loss to follow-up plus Model 2. For PP analysis, artificial censoring due to deviation from initial treatment during time-at-risk period was additionally accounted for.
Abbreviations: CI, confidence interval; HR, hazard ratio.
